# Functionally oriented analysis of cardiometabolic traits in a trans-ethnic sample

**DOI:** 10.1093/hmg/ddy435

**Published:** 2019-01-08

**Authors:** Lauren E Petty, Heather M Highland, Eric R Gamazon, Hao Hu, Mandar Karhade, Hung-Hsin Chen, Paul S de Vries, Megan L Grove, David Aguilar, Graeme I Bell, Chad D Huff, Craig L Hanis, HarshaVardhan Doddapaneni, Donna M Munzy, Richard A Gibbs, Jianzhong Ma, Esteban J Parra, Miguel Cruz, Adan Valladares-Salgado, Dan E Arking, Alvaro Barbeira, Hae Kyung Im, Alanna C Morrison, Eric Boerwinkle, Jennifer E Below

**Affiliations:** 1 Vanderbilt Genetics Institute, Vanderbilt University Medical Center, Nashville, TN, USA; 2 Human Genetics Center, School of Public Health, The University of Texas Health Science Center at Houston, Houston, TX, USA; 3 Department of Epidemiology, University of North Carolina, Chapel Hill, NC, USA; 4 Clare Hall, University of Cambridge, Cambridge, UK; 5 Department of Epidemiology, MD Anderson Cancer Center, Houston, TX, USA; 6 Department of Cardiology, Baylor College of Medicine Houston, TX, USA; 7 Departments of Medicine and Human Genetics, The University of Chicago, Chicago, IL, USA; 8 Human Genome Sequencing Center, Baylor College of Medicine, Houston, TX, USA; 9 Department of Anthropology, University of Toronto at Mississauga, Mississauga, Ontario, Canada; 10 Unidad de Investigación Médica en Bioquímica, Hospital de Especialidades, Centro Médico Nacional Siglo XXI, IMSS, Mexico City, Mexico; 11 McKusick-Nathans Institute of Genetic Medicine, Johns Hopkins University School of Medicine, Baltimore, MD, USA; 12 Section of Genetic Medicine, Department of Medicine, University of Chicago, IL, USA

## Abstract

Interpretation of genetic association results is difficult because signals often lack biological context. To generate hypotheses of the functional genetic etiology of complex cardiometabolic traits, we estimated the genetically determined component of gene expression from common variants using PrediXcan (1) and determined genes with differential predicted expression by trait. PrediXcan imputes tissue-specific expression levels from genetic variation using variant-level effect on gene expression in transcriptome data. To explore the value of imputed genetically regulated gene expression (GReX) models across different ancestral populations, we evaluated imputed expression levels for predictive accuracy genome-wide in RNA sequence data in samples drawn from European-ancestry and African-ancestry populations and identified substantial predictive power using European-derived models in a non-European target population. We then tested the association of GReX on 15 cardiometabolic traits including blood lipid levels, body mass index, height, blood pressure, fasting glucose and insulin, RR interval, fibrinogen level, factor VII level and white blood cell and platelet counts in 15 755 individuals across three ancestry groups, resulting in 20 novel gene-phenotype associations reaching experiment-wide significance across ancestries. In addition, we identified 18 significant novel gene-phenotype associations in our ancestry-specific analyses. Top associations were assessed for additional support via query of S-PrediXcan (2) results derived from publicly available genome-wide association studies summary data. Collectively, these findings illustrate the utility of transcriptome-based imputation models for discovery of cardiometabolic effect genes in a diverse dataset.

## Introduction

Cardiometabolic traits are highly heritable, with heritability estimates ranging from ~0.3 to 0.8 (3–5). Through genome-wide association studies (GWAS) and exome-chip based association studies, genetic researchers have identified more than a thousand common and low-frequency variants contributing to the risk of cardiometabolic traits to date, yet only a small fraction of the heritability of these traits [e.g. 27% for height on the high end (6)], has been explained. Furthermore, the molecular mechanisms through which identified cardiometabolic variants confer risk are largely unknown. Some relevant molecular pathways have been identified via gene set enrichment and gene ontology analyses, often using the nearest (in linear distance) genes (6–11); however, signals often occur far from a protein coding region or functional element in the genome leading to weak assumptions about the relevant biology. More recently, interrogations of low-frequency coding variation via exome chip analysis identified a handful of novel loci, but minimal additional insight into the potential functionality of previously known common loci was gained from these findings (6,10).

Regulation of gene expression is influenced by many factors including tissue or cell type, developmental or life stage, environmental exposures, and genetic factors (12–16) such as promoter and enhancer sequence variation (17). Expression levels of certain genes may be a predictor of future disease or a biomarker of current disease (18,19), and many noncoding variants are associated with RNA abundance [i.e. expression quantitative trait loci (eQTLs)] and multiple eQTLs may jointly genetically regulate expression of a particular gene. Recent studies suggest that top hits in many GWAS for complex traits are enriched for eQTLs, allowing for interpretation of GWAS signals in the context of their role in gene expression regulation (20–23). By aggregating variant effects at the gene level, by design, results of genetically regulated gene expression (GReX)-based analyses are directly interpretable within a functional biological framework and also benefit from reduced multiple testing correction. As in traditional genome-wide association studies, GReX-based analyses are hypothesis generating rather than prescriptive, and are also susceptible to synthetic associations due to co-regulation of genes. However, in some instances distinct expression regulation at neighboring genes can be leveraged to target putatively functional genes in disease etiology, generating testable hypotheses of causality. Further, GReX-based genome-wide association tests may avoid some of the pitfalls of measured gene expression at a single time point, because genetically controlled traits often represent life-long influences, without the environmental, batch and temporal effects captured by measured gene expression (24).

Methodologically, two approaches to GReX estimation have been proposed: elastic net regression implemented in PrediXcan (1), and Bayesian Sparse Linear Mixed Models (BSLMM) implemented in TWAS, a transcriptome-wide association study approach (25). In PrediXcan, GReX panels are built with elastic net regression using cross-validation to prevent model overfitting (1); elastic net regression is an excellent variable selection method for `wide’ datasets with many predictors/features (26) and a sparse architecture, where a limited number of features have relatively large effect sizes. The TWAS-implemented BSLMM approach for GReX estimation assumes a mixture of both sparse and broad polygenic effects (25)—allowing for many small effect sizes distributed across many predictors. Comparisons of these two approaches indicate that on the tissue level, gene expression traits appear to be driven by a set of sparse, large effect variants (27). Additional analyses also illustrate that interactions among eQTL variants from lymphoblastoid cell lines likely do not influence gene expression traits to any strong degree (28), though this hypothesis remains to be tested in primary tissues. The expression imputation models we employ in PrediXcan were created by jointly estimating the effects of genomic variants on transcriptome data in large datasets such as the Genotype-Tissue Expression project (GTEx v6p) (29,30) and Depression Genes and Networks (DGN) (31) study. Using PrediXcan-estimated GReX on the large collection of cardiometabolic GWAS studies will achieve two simultaneous goals: (1) the aggregation of multiple SNP effects into a single functional unit increases statistical power for discovery and (2) the detection of a significant result generates a hypothesis that a specific gene (and potentially higher biological mechanisms) may be important for cardiometabolic risk.

## Results

### Model assessment

**Figure 1:**
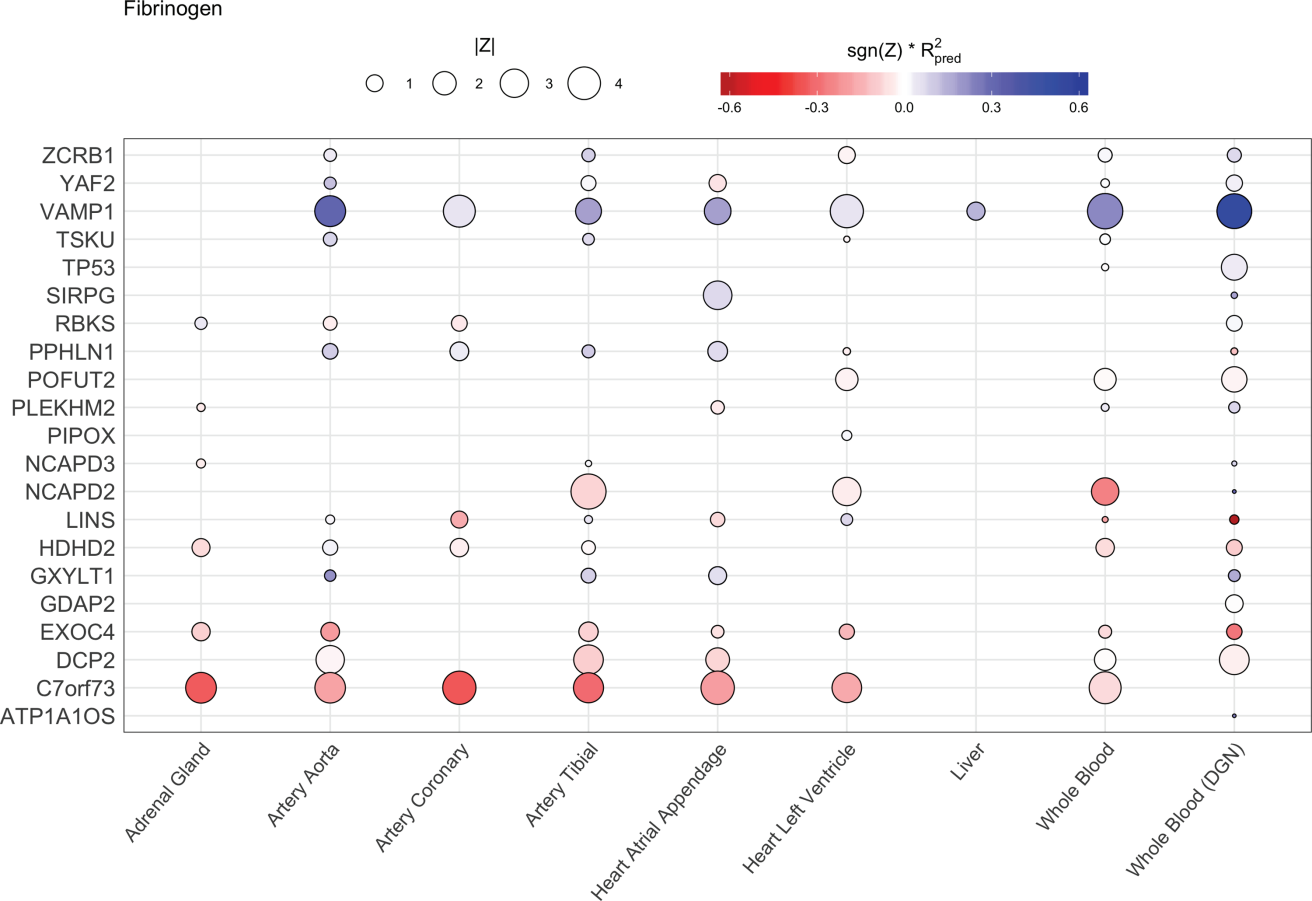
Z-scores for novel gene GReX-trait association across relevant tissues for fibrinogen. Comparison of Z-scores across all cardiovascular trait-assessed tissues for a single trait, fibrinogen, demonstrating concordance of effects for each gene. Size of the point represents magnitude, color indicates direction of effect (blue points are positively associated with the trait, red points are negatively associated with the trait), and shade indicates the R^2^ from the prediction model. Missing points indicate that there is no model available for the given gene in that tissue.

Existing work has confirmed the applicability of genotype-based gene expression prediction in diverse training or target populations (32); however, because we applied GReX models built from RNA-sequencing data generated from tissues collected from individuals of primarily European descent in datasets drawn from non-European populations, we performed a number of experiments to evaluate the quality of cross-ancestry expression prediction in available data. We directly assessed the accuracy of PrediXcan-estimated GReX in our study data by comparing imputed whole blood expression and measured WB RNA sequencing levels in a subset of 175 European-ancestry ARIC samples. Using whole blood DGN (DGN-WB) imputed GReX, the squared correlation coefficient of the directly measured RNA expression with PrediXcan-derived GReX deviated significantly from the expected R^2^ distribution at the tail end of the observed distribution, indicating the predictive utility of these externally-built models in our samples (Supplementary Material, Fig. S1). An additional comparison to the gene’s heritability, as assessed in the data on which the PrediXcan models were trained, is made in the same figure (1). Because our imputations are estimating GReX, not RNA abundance, the heritability of the expression for that gene sets an upper bound for how well we can impute its expression using genetic variation. Imputed expression in the ARIC samples captured up to 75% of the variance in the observed expression. Notably, we found a significant correlation between the cross-validation R^2^ from the model building step (trained in the European-ancestry DGN-WB samples) and the explained variance (in measured expression captured by GReX) in the ARIC samples (Spearman correlation = 0.70, *P* < 2.2e-16). Top results are annotated with the ARIC R^2^/h^2^ values (Table 1 and Supplementary Material, Table S1).

**Table 1:** Significant novel associations. Novel gene expression-trait associations significant at *P_BH_* < 0.05

**Trait**	**Gene name**	**Location**	**Tissue**	**Effect**	**StdErr**	**P**_**BH**_	***P*-value**	**Direction**	**N.SNPs**	**Gene2Pheno Validation**	**Qval**	***P*-value (EA)**	***P*-value (AA)**	***P*-value (HL)**	**Pearson’s r** ^**2**^ **/h** ^**2**^
**All Ancestry**															
DBP	*LINS*	15:101099574–101143435	Heart atrial appendage	−0.142	0.033	4.11E-02	1.27E-05	++−−−	55	TNA	9.44E-04	3.00E-05	8.01E-02	5.39E-01	1.047
DBP	*POFUT2*	21:46683843–46707813	Whole blood	5.372	1.215	3.30E-02	9.75E-06	+?+++	1	TNA	1.42E-02	3.35E-04	3.02E-03	2.46E-01	0.729
Fibrinogen	*VAMP1*	12:6571403–6580153	Whole blood (DGN)	0.066	0.015	2.14E-02	5.56E-06	++	27	TNA	5.90E-150	4.17E-04	1.37E-03	NA	1.431
Fibrinogen	*VAMP1*	12:6571403–6580153	Whole blood	0.119	0.025	1.11E-02	2.63E-06	++	22	TNA	6.53E-20	2.44E-04	1.36E-03	NA	1.431
Fibrinogen	*NCAPD2*	12:6602522–6641121	Artery tibial	−0.269	0.058	1.34E-02	3.32E-06	−	9	TNA	8.62E-07	2.20E-04	1.48E-03	NA	1.043
FVII	*EXOC4*	7:132937829–133751342	Whole blood (DGN)	0.101	0.021	4.16E-03	7.80E-07	++	122	TNA	1.72E-66	1.22E-04	2.66E-04	NA	0.711
FVII	*EXOC4*	7:132937829–133751342	Whole blood	0.278	0.056	3.05E-03	5.37E-07	++	61	TNA	7.18E-07	1.81E-04	1.13E-04	NA	0.711
FVII	*TSKU*	11:76493295–76509198	Artery aorta	−0.348	0.063	3.18E-04	3.90E-08	−	15	TNA	7.78E-05	1.74E-09	9.93E-01	NA	NA
FVII	*TSKU*	11:76493295–76509198	Artery tibial	−0.328	0.068	5.69E-03	1.21E-06	−	6	TNA	4.21E-06	8.63E-07	5.27E-01	NA	NA
FVII	*TSKU*	11:76493295–76509198	Heart left ventricle	−0.665	0.116	1.05E-04	1.07E-08	−	5	TNA	2.89E-02	1.84E-08	3.17E-01	NA	NA
FVII	*GXYLT1*	12:42475647–42538681	Whole blood (DGN)	0.142	0.028	1.71E-03	2.59E-07	++	35	TNA	2.06E-35	6.78E-07	7.55E-02	NA	0.983
FVII	*GXYLT1*	12:42475647–42538681	Artery aorta	0.158	0.028	1.15E-04	1.23E-08	++	27	TNA	4.75E-11	7.05E-08	5.01E-02	NA	0.983
FVII	*GXYLT1*	12:42475647–42538681	Artery tibial	0.205	0.040	2.12E-03	3.38E-07	++	24	TNA	4.43E-07	3.08E-06	2.32E-02	NA	0.983
FVII	*YAF2*	12:42550906–42632151	Whole blood	0.445	0.096	1.45E-02	3.63E-06	++	10	TNA	2.75E-02	8.44E-05	1.13E-02	NA	0.867
FVII	*PPHLN1*	12:42632249–42853517	Whole blood (DGN)	0.166	0.038	4.65E-02	1.49E-05	++	19	TNA	5.20E-27	9.23E-05	4.11E-02	NA	0.880
FVII	*PPHLN1*	12:42632249–42853517	Artery coronary	0.198	0.045	3.01E-02	8.52E-06	++	37	TNA	4.10E-02	1.27E-04	5.39E-03	NA	0.880
FVII	*PPHLN1*	12:42632249–42853517	Artery tibial	0.236	0.048	5.03E-03	1.03E-06	++	13	TNA	1.40E-07	2.44E-05	3.84E-03	NA	0.880
FVII	*ZCRB1*	12:42705880–42719920	Whole blood (DGN)	0.223	0.047	8.15E-03	1.81E-06	++	28	TNA	1.20E-15	3.80E-05	7.01E-03	NA	0.736
FVII	*ZCRB1*	12:42705880–42719920	Whole blood	0.320	0.071	2.55E-02	6.88E-06	++	20	TNA	5.05E-03	8.06E-05	2.13E-02	NA	0.736
height	*LGR6*	1:202163029–202288909	Whole blood (DGN)	0.124	0.026	7.15E-03	1.57E-06	+++++	38	4.80E-01	1.10E-37	1.39E-04	9.01E-02	3.16E-03	0.637
height	*GYPE*	4:144792020–144826716	Adipose visceral omentum	−0.129	0.029	2.83E-02	7.87E-06	−	40	2.60E-05	8.82E-06	2.51E-03	1.54E-01	7.46E-04	1.531
Insulin	*FAM175A*	4:84382092–84444501	Whole blood (DGN)	−0.201	0.046	4.37E-02	1.39E-05	−	27	4.80E-01	1.17E-15	4.67E-05	7.12E-02	NA	0.730
Insulin	*PHOSPHO1*	17:47300724–47308128	Muscle skeletal	0.257	0.053	5.66E-03	1.19E-06	+++	24	NR	5.18E-09	2.03E-06	2.26E-01	9.01E-01	0.411
LDL	*ZNF441*	19:11877815–11894893	Pancreas	0.391	0.072	4.39E-04	5.58E-08	+−+++	53	NR	1.47E-03	1.93E-06	1.42E-01	1.50E-02	NA
Triglycerides	*C2orf70*	2:26785450–26802400	Pancreas	−4.400	0.971	2.24E-02	5.89E-06	−?−−−	1	NR	3.55E-04	7.34E-05	8.52E-01	7.23E-04	NA
Triglycerides	*PCBP4*	3:51991470–52002426	Liver	−0.223	0.049	2.32E-02	6.19E-06	−?+−−	15	NR	2.98E-02	8.72E-07	6.02E-01	3.84E-01	NA
Triglycerides	*TAF6L*	11:62538775–62554814	Whole blood (DGN)	0.312	0.065	6.42E-03	1.40E-06	+++++	57	2.20E-08	6.48E-03	2.01E-06	6.73E-01	5.46E-02	0.232
Triglycerides	*APOL5*	22:36113919–36125530	Adipose visceral omentum	−0.202	0.043	1.01E-02	2.34E-06	−	46	9.40E-02	1.20E-02	1.62E-04	1.65E-02	3.85E-02	NA
WBC	*DCP2*	5:112312399–112356667	Artery aorta	−0.331	0.075	3.07E-02	8.78E-06	−	25	TNA	2.31E-02	3.69E-05	3.25E-02	NA	0.414
**European ancestry**															
BMI	*ZDHHC6*	10:114190058–114206672	Adipose subcutaneous	0.250	0.055	2.13E-02	5.11E-06	−−−+−	63	6.10E-01	8.40E-05	5.11E-06	2.22E-01	3.12E-01	0.712
BMI	*NUP107*	12:69080514–69136785	Adrenal gland	−0.198	0.044	3.20E-02	8.04E-06	−+−−+	27	1.60E-01	1.99E-03	8.04E-06	1.64E-01	2.56E-01	0.566
Cholesterol	*ALDH4A1*	1:19197926–19229275	Whole blood (DGN)	0.321	0.071	2.58E-02	6.37E-06	+++++	28	HDL: 0.57 LDL: 0.04	2.60E-11	6.37E-06	8.83E-01	2.25E-01	1.453
Cholesterol	*SNRNP48*	6:7590432–7612200	Whole blood	0.353	0.079	3.22E-02	8.14E-06	−++++	32	HDL: 0.83 LDL: 0.21	4.76E-03	8.14E-06	3.73E-01	6.05E-01	0.587
Cholesterol	*BTBD3*	20:11871371–11907257	Whole blood (DGN)	−0.432	0.079	3.99E-04	4.71E-08	+−−−+	16	HDL: 0.43 LDL: 0.81	4.73E-09	4.71E-08	4.23E-01	8.09E-01	0.780
Cholesterol	*BTBD3*	20:11871371–11907257	Whole blood	−1.039	0.204	2.40E-03	3.64E-07	−?+−+	4	NR	1.52E-03	3.64E-07	5.98E-01	9.93E-01	0.780
Fibrinogen	*C7orf73*	7:135347244–135378166	Whole blood	−0.223	0.051	3.88E-02	1.05E-05	−+	29	TNA	5.69E-07	1.05E-05	7.92E-01	NA	NA
height	*SH3BGRL2*	6:80341000–80413372	Adipose visceral omentum	−0.153	0.035	4.90E-02	1.40E-05	+?−−+	14	1.80E-03	2.61E-09	1.40E-05	7.65E-01	6.35E-01	0.508
LDL	*BTBD3*	20:11871371–11907257	Whole blood (DGN)	−0.388	0.080	6.65E-03	1.16E-06	+−−−+	16	8.10E-01	4.73E-09	1.16E-06	4.66E-01	5.68E-01	0.780
LDL	*BTBD3*	20:11871371–11907257	Whole blood	−0.969	0.206	1.23E-02	2.54E-06	−?+−+	4	NR	1.52E-03	2.54E-06	6.57E-01	9.74E-01	0.780
Platelet count	*TP53*	17:7565097–7590856	Whole blood	3.722	0.852	4.48E-02	1.27E-05	+−	10	TNA	2.38E-02	1.27E-05	8.93E-01	NA	0.638
Platelet count	*PIPOX*	17:27277531–27384234	Heart left ventricle	0.355	0.077	1.90E-02	4.41E-06	+−	23	TNA	3.44E-02	4.41E-06	2.07E-01	NA	NA
Triglycerides	*RBKS*	2:28004231–28113965	Muscle skeletal	−1.405	0.296	1.08E-02	2.14E-06	−?−−−	21	NR	3.86E-02	2.14E-06	2.82E-01	1.77E-01	0.362
WBC	*HDHD2*	18:44633774–44676891	Adrenal gland	−0.286	0.065	4.08E-02	1.14E-05	−+	7	TNA	4.11E-03	1.14E-05	9.75E-01	NA	1.074
WBC	*SIRPG*	20:1609798–1638425	Heart atrial appendage	0.251	0.054	1.63E-02	3.70E-06	+−	33	TNA	1.01E-03	3.70E-06	2.31E-02	NA	0.817
**African ancestry**															
height	*RCBTB1*	13:50106082–50159719	Adrenal gland	0.910	0.180	5.84E-03	4.55E-07	−?−++	13	4.20E-01	3.98E-02	9.14E-01	4.55E-07	1.48E-01	1.081
Platelet count	*PLEKHM2*	1:16010827–16061264	Whole blood (DGN)	−0.557	0.117	1.77E-02	1.93E-06	+−	13	TNA	5.62E-17	6.45E-01	1.93E-06	NA	1.024
Platelet count	*PLEKHM2*	1:16010827–16061264	TW whole blood	−0.884	0.189	2.72E-02	3.19E-06	+−	3	TNA	1.04E-04	6.05E-01	3.19E-06	NA	1.024
SBP	*NCAPD3*	11:134020014–134095348	TW adrenal gland	9.584	1.948	1.07E-02	9.77E-07	−?+−+	2	TNA	2.71E-02	9.12E-01	9.77E-07	8.78E-01	0.742
WBC	*ATP1A1OS*	1:116948131–116961158	Whole blood (DGN)	−0.353	0.065	9.65E-04	6.22E-08	+−	11	TNA	5.58E-55	4.14E-01	6.22E-08	NA	NA
WBC	*GDAP2*	1:118406107–118472253	Whole blood (DGN)	1.503	0.294	4.52E-03	3.32E-07	−+	24	TNA	4.70E-02	2.01E-02	3.32E-07	NA	0.230

Gene2Pheno GIANT BMI analysis included ARIC EA and AA;

Gene2Pheno MAGIC (Glucose and Insulin) analyses included ARIC EA;

Gene2Pheno GLGC (lipids) analyses included ARIC EA;

N.SNPs is the number of SNPs used to predict GREx;

P-value (MA) is the meta-analysis across all three ancestires;

P-value (EA) is the European specific GREx by trait association p-value;

P-value (AA) is the African American specific GREx by trait association p-value;

P-value (HL) is the meta-analysis p-value across the three Hispanic/Latino studies;

Qval is adjusted for false discovery rate, from PrediXcan model;

Pearson's r2/h2 is squared correlation coefficient between predicted and observed whole blood (DGN) expression / PrediXcan local heritability estimate ;

NR indicates no results were available for the gene-tissue combination in Gene2Pheno;

TNA indicates that the traits was not available in a Gene2Pheno database.

Using the same tissue (whole blood), we found significant correlations (*P* < 2.2e-16; Supplementary Material, Fig. S2) of z-score for DGN- and GTEx-trained models for each study phenotype, indicating that association results obtained from the application of models derived from the ancestrally heterogeneous GTEx-WB and the homogenous (European ancestry) DGN-WB showed a high degree of concordance. To verify the robustness of our trans-ethnic analysis, we conducted additional analyses to assess the utility of PrediXcan models developed in DGN and GTEx in non-European populations, using African ancestry YRI LCL samples. We find that imputation quality (cross-validation R^2^) in European ancestry DGN-WB was significantly associated (*P* = 0.007) with imputation performance in the African ancestry YRI samples, though that correlation between imputed and measured expression is lower, on average, in the African population (Supplementary Material, Fig. S3). We also observe that among models with high R^2^ (presumed to be well modeled based on the training data), we see highly significant correlation of imputed and measured expression, with a substantial number of genes with correlation *P* < 1e-3 (Supplementary Material, Fig. S4). In summary, although we detect that predictive power is slightly reduced, PrediXcan retains substantial predictive power when European-derived models are applied to non-European samples. Until more equal representation of non-European populations in publicly available gene expression datasets is attained and ancestry-specific expression prediction models generated for the subset of genes with ancestry-specific genetic regulation, application of GTEx derived models is likely to retain power to detect effects at most genes in most tissues in datasets drawn from non-European populations.

### Gene-trait associations

In 15755 samples comprised of African Americans, European Americans and Hispanics/Latinos from the USA and Mexico (Supplementary Material, Table S2), we assessed the association of imputed tissue-specific GReX level in biologically relevant tissues with 15 cardiometabolic traits (Table 1, Supplementary Material, Table S1). We detected many genes associated with white blood cell (WBC) count in the well-known *DARC* locus, however, due to extensive linkage disequilibrium, all further results exclude genes in this region (spanning 1q21.1 to 1q23.3). Across the 15 traits, GReX of 167 unique gene-phenotype associations (270 gene-phenotype-tissue associations) were significant (experiment-wide BH-adjusted *P* < 0.05) in at least one biologically relevant tissue in ancestry-specific analyses or trans-ancestry meta-analysis (Supplementary Material, Fig. S5). Thirty-eight of these are novel, significant gene-trait associations including 20 experiment-wide significant trans-ancestry results and 18 such ancestry-specific findings (Table 1). Of our 270 total significant tissue-specific gene-trait associations, Gene2Pheno results were available for 126 known and 13 novel gene-trait-tissue combinations; 122 known (96%) and 2 (13%) novel genes were associated with the same trait in the same tissue(s) (*P* < 3.60e-4, based on Bonferroni correction for the number of Gene2Pheno genes queried).

In height, we observed four novel gene-trait associations: *LGR6* in DGN-WB and *GYPE* in visceral omentum adipose tissue in the trans-ancestry meta-analysis, *SH3BGTL2* in visceral omentum adipose in European ancestry, and *RCBTB1* in adrenal gland in African Americans. *GYPE* was also significant in Gene2Pheno (*P* = 2.6e-05). We observe novel associations between fasting insulin levels and *PHOSPHO1* in skeletal muscle and DGN-WB (European ancestry), and *FAM175A* in DGN-WB. Five genes not previously associated in GWAS of serum lipid levels were identified by trans-ancestry meta-analysis: *ZNF441* (LDL, pancreas); *TAF6L* (triglycerides, WB); *APOL5* (triglycerides, visceral omentum adipose); *C2orf70* (triglycerides, pancreas); and *PCBP4* (triglycerides, liver). Additional European-specific associations were observed, including *BTBD3* for LDL and cholesterol in WB and DGN-WB; and *RBKS* for triglycerides in skeletal muscle. *TAF6L* reached significance in Gene2Pheno; results for *C2orf70* and *PCBP4* were not available in Gene2Pheno. Despite being a compelling candidate, *APOL5* was not significant in Gene2Pheno results for association with triglycerides in our discovery tissue (visceral omentum adipose, *P* = 0.094).

Novel findings among blood and vascular traits were discovered in trans-ancestry analyses of factor VII, DBP, fibrinogen, platelet count and WBC count; African–American-specific analyses for SBP, platelet count and WBC count (Table 1). Results for WBC count were strongly enriched in the *DARC* locus, with 57 unique gene-phenotype associations meeting our significance threshold, primarily driven by African Americans (Supplementary Material, Table S1) (33). Three regions had significant GReX associations with factor VII level in the trans-ancestry meta-analysis and were not observed in prior GWAS, all associated in multiple tissues. *TSKU* was significantly associated in aortic artery, tibial artery and left ventricle tissue, genes in 12.q12 (including *GXYLT1*) in aortic artery, tibial artery and DGN-WB, and *EXOC4* in DGN-WB and WB. *TSKU* was previously implicated in a sub-analysis of a recent GWAS, when analyses were restricted to only factor VII activity (excluding factor VII antigen) (34).

### Pathway analysis

In total, 50 annotation terms were significantly over-represented, most of which were identified for factor VII and WBC count (Supplementary Material, Table S3). Total cholesterol, HDL cholesterol, LDL cholesterol and triglycerides also each had at least one term over-represented in the corresponding candidate gene sets. The most significant terms for these traits are early endosome (cholesterol), alcohol dehydrogenase activity (FVII), very-low-density lipoprotein particle remodeling (HDL), lipid metabolism (LDL), lipoprotein metabolic process (triglycerides) and immunoglobulin domain (WBC).

Among the novel genes identified for any trait, there were four that showed evidence of protein–protein interaction with known genes for that trait in STRING (Supplementary Material, Table S4). STRING considers evidence from seven sources in establishing a functional association score. For platelet count, *TP53* is predicted to interact with known-gene *BAK1* with a combined score of 0.993, with strong evidence of interaction from the expertly curated database score and being frequently co-mentioned in PubMed abstracts. For body mass index (BMI), *NUP107* shows evidence of interaction with known-gene *NUP160* with a score of 0.999, based on experimental biochemical evidence and correlation of gene expression, as well as the databased and co-mentioned scores. For factor VII, *GXYLT1* has a combined interaction score of 0.762 with *F7*, with most evidence due to being co-mentioned. Finally, for height, *LGR6* shows modest evidence of interaction with *GNA12*, with a score of 0.557 based on experimental evidence and being co-mentioned in PubMed.

## Discussion

Approaches that leverage genetically regulated expression prediction, although still fairly new, have been the subject of appreciable criticism. In brief, the primary concerns are that these approaches do not imply causality, are vulnerable to identifying synthetic associations due to co-regulation of genes, can be sensitive to the tissue or expression panel that is used, and ultimately only amount to a weighted burden test. These concerns are valid, but also apply to the most commonly used approach in the past era of complex disease genetics: the genome-wide association study. Just as SNPs identified by GWAS may not in fact be causal, GReX-based approaches are intended to be hypothesis generating, identifying gene candidates whose expression may impact a trait under study; similarly, SNPs in GWAS may be identified due to synthetic associations. GReX-based association analyses are indeed analogous to a weighted burden test—one that uses functional impact on expression as a weight—and so it is logical that they would be sensitive to the same confounding factors as the single variant association statistics that are being essentially combined. As SNPs identified in GWAS must be functionally validated to establish causality, so must genes implicated by PrediXcan.

Despite these methodological challenges, for cardiometabolic traits we have identified 38 novel genes in our trans-ancestry and ancestry specific analyses, and replicated known signals at 289 genes. These results represent novel hypotheses of the causal genes underlying new and known GWAS signals. In addition, we explored the utility of GReX prediction models derived from Caucasian datasets in non-Caucasian ancestral populations, and determined that substantial power for GReX estimation is maintained at many genes; further scientific investment in increasing tissue and ancestral diversity of transcriptome datasets will be necessary to capture ancestry-specific eQTL effects.

Among our most biologically interesting novel associations were four genes on chromosome associated with FVII levels. Imputed expression levels of *PPHLN1*, *ZCRB1*, *YAF2* and *GXYLT1* were strongly correlated with each other in whole blood (minimum r^2^ = 0.46 and maximum r^2^ = 0.83, respectively). *GXYLT1* adds the first xylose to O-glucose-modified residues in the epidermal growth factor repeats of proteins. One of these proteins may be factor VII: factor VII does have epidermal growth factor repeats, and is known to be O-linked glycosylated (35). Prior functional work has demonstrated that inducing a mutation affecting the amino acid that is glycosylated reduces the activity of FVII (36).

Other novel associations with compelling functional evidence include *VAMP1* predicted expression with fibrinogen levels and *PHOSPHO1* predicted expression with fasting insulin. Members of the VAMP family of proteins are involved in vesicle secretion, including alpha granules, which contain fibrinogen (37,38). The precise function of VAMP1 has not been previously described, but our finding, that GReX is positively associated fibrinogen levels, suggests that like other members of this protein family it may be involved in platelet secretion. Increased *PHOSPHO1*-imputed expression was associated with higher insulin levels; previous literature reports that decreased methylation status in the body of these gene increases risk of developing type 2 diabetes (39). Additional work shows that methylation is decreased in skeletal muscle samples from T2D cases in comparison to controls (40).

We also provide new support for some genes within known regions, but with weak or population-specific prior evidence. For example, the association between DGN-WB GReX of platelet-activating factor *PAFAH1B2* with triglyceride level had strong additional support in Gene2Pheno (replication *P* = 1.1 × 10^-17^, Table 1). Previous GWAS evidence comes from a Japanese study, in which the identified variant has a MAF of 11% (41). Further, a single rare coding variant in this gene was recently found to have a large effect on triglyceride and HDL levels in European Americans (42). Our findings complement this discovery, expanding the evidence that this gene impacts triglyceride level via common eQTL effects as well as rare functional variation. Because triglyceride level is a causal factor for cardiovascular disease, we explored Gene2Pheno results for *PAFAH1B2* in the additive analysis of coronary artery disease in CARDIoGRAM C4D. In DGN-WB, we observe *P =* 0.014 in Gene2Pheno, with the same magnitude and direction of effect on triglycerides as we observed from *PAFAH1B2* in the same tissue, for the first time suggesting that this gene may impact cardiovascular disease risk via effects on triglyceride levels.

For significant known genes, an additional analysis conditional on the known, sentinel variant in the gene or locus was undertaken, when available, to determine independence of the identified GReX signal with the previously identified signal. For 41 tissue- and trait-specific gene associations, including 25 unique genes, the genetically determined expression shows evidence of independent association with the trait of interest, beyond the known single variant signal (conditional *P* < 0.05). Additionally, three genes, *F10* for Factor VII, and *SORT1* and *SYPL2* for LDL retained *P* < 2.5e-6 after accounting for the sentinel SNP, demonstrating an independent signal. These results highlight the utility of eQTL evidence to clarify the biology underlying genome-wide association signals, both through identification of novel, independent signals in the same locus and through the refinement of mapping effects. Rather than attributing results simply to the nearest neighbor gene, instead we identify genes where predicted expression is significantly influenced by eQTLs in LD with the association signal.

There are several reasons a particular known locus would not be detected in our analyses: (1) our total sample size is substantially smaller than the large-scale meta-analyses available; therefore, we have less power, despite our reduction in tests. (2) The biological mechanism underlying previous associations may not be well represented through PrediXcan. For example, changes in amino acid sequence may result in changes in protein function but not expression. (3) Changes in expression may occur in one tissue or cell type that is not captured in the GTEx data. (4) Ancestry-specific associations may act through gene expression changes that are not captured in the predominantly European expression datasets. Despite these limitations, we replicated hundreds of known loci for the 15 cardiometabolic traits under study.

Most (289 of 339) of our associations fall within 250 kilobases (kb; or larger in regions of extended LD) of variants previously associated with the same trait; however, only a small fraction of all known loci is detected (e.g. 22 of 157 identified for lipids traits) (11). On the whole, we observe an excess of association signals at genes within 250 kb of previously reported SNPs (Supplementary Material, Fig. S6). Just as traditional SNP-based GWAS narrows, often considerably, a disease association to (and thus proposes) a disease-relevant locus (which must subsequently undergo fine-mapping to identify the causal variants(s)), our approach proposes a disease-relevant gene expression mechanism (whose tissue or indeed cell-type specificity must be further resolved). Therefore, genes identified at known loci in these analyses represent a proposed refinement of the causal biological factor underlying the previously described single variant association signal.

We note that many of our results are observed across several tissues, consistent with some level of shared regulation across tissues (43). To visualize the concordance of significance across tissues for each novel gene, we plotted Z-scores for GReX-trait association across all relevant tissues for each trait (Fig. 1 and Supplementary Material, Fig. S7). This suggests that genetically regulated expression associated with disease risk may implicate multiple tissues, some or all of which may be relevant to disease pathogenesis. Future *in vitro* experimentation will be required to understand which tissues’ altered expression is responsible for the increased risk.

The Database for Annotation, Visualization and Integrated Discovery (DAVID) analyses provide additional support for novel genes identified by our study, some in regions previously implicated in GWAS, where historically the signals have been attributed to other genes (often through physical proximity rather than through any functional support) (44). Two novel genes identified herein comprise 13 overrepresented pathway results, contributing to the observed overrepresentation of annotation terms (Supplementary Material, Table S3; Benjamini FDR < 0.20). For WBC count, Signal Regulatory Protein Gamma (*SIRPG*, a transmembrane glycoprotein) was annotated with nine overrepresented annotation terms, including several related to immunoglobulin, disulfide bond, signal peptide and extracellular domain. *FCRL1*, a known gene for WBC count, was highly significant (*p_BH_* = 4.83E-10) in our African ancestry sample and annotated with similar annotation terms (Supplementary Material, Table S3), illustrating novel trait-associated genes (each in a distinct locus) with highly convergent function. One novel gene not previously implicated in GWAS of lipids traits (despite the much larger sample size (11)), *APOL5* (from the meta-analysis of triglycerides), is a biologically compelling member of the high-density apolipoprotein L family which is known to play a major role in cholesterol transport, though is known to have a distinct origin from the *APOL1*-*APOL4* cluster and has not been previously observed in genetic studies (45,46). *APOL5* contributed to the overrepresentation of genes involved in lipid and lipoprotein metabolic processes (Supplementary Material, Table S3), which are implicated in known genes for lipids traits. These examples suggest that our findings, collectively, may challenge existing understanding of the relevant genes in known GWAS loci.

## Conclusions

Functionally orientating analyses of common variants around gene expression improves biological interpretation and power through reduced multiple testing correction. This approach focuses on *cis*-acting variants that alter gene expression without the burden of obtaining tissue samples and directly measuring RNA abundance genome-wide. Our imputed-GReX analysis of 15 cardiometabolic traits in 15755 individuals across three ancestry groups has revealed 38 novel gene-trait associations in new regions, 18 of which were identified in ancestry-specific analyses. Our findings provide potential function-based refinement or support of an additional 289 genes previously implicated in GWAS. Most significant associations reached the Bonferroni-corrected significance threshold in Gene2Pheno results, where available, indicating strong replication rate. We found that directly measured WB RNA abundance strongly correlates with imputed expression level at genes with low FDR and that models developed in mostly European ancestry retain predictive power in African samples, providing justification for our trans-ancestry approach. These findings support the validity of the known and novel associations we describe, while highlighting the need for further investment in RNA sequencing data from diverse samples. Furthermore, many of these findings are supported by gene ontology pathway and protein–protein interaction analyses, including novel findings and genes that have not previously been mapped to known GWAS signals. Natural extensions of these methods to include *trans*-acting eQTLs and other functionally annotated regions of the genome such as those described by ENCODE may provide further power to detect new genes impacting complex disease traits and refine GWAS signals to identify candidates for functional validation.

## Materials and Methods

### Subjects by parent study

ARIC: The Atherosclerosis Risk in Communities (ARIC) study is a prospective epidemiological study designed to investigate the etiology and predictors of cardiovascular disease. The ARIC study enrolled 15 792 individuals in total, primarily of European and African ancestry, aged 45–64 years from four US communities (Forsyth County, NC; Jackson, MS; suburbs of Minneapolis, MN; and Washington County, MD) in 1987–89 (baseline) and followed for four completed visits in 1990–92, 1993–95, 1996–98 and 2011–13 with a sixth visit ongoing. 9131 European Americans and 2652 African Americans were included in the present analysis. A detailed description of the ARIC study design and methods was previously published (47).

Starr County: Phenotypic measures for 1006 Mexican Americans from Starr County, Texas included in this work were generated in a survey of a representative population of the county. Details of the collection are previously described (48).

Mexico City: Two datasets were available from Mexico City. Both datasets were collected for studies aimed at identifying type 2 diabetes risk factors using genome-wide approaches. The first study includes 967 individuals with type 2 diabetes and 343 controls. The second study comprises 898 individuals with type 2 diabetes and 889 controls. All individuals were included in the present study. Detailed information about the Mexico City datasets has been previously published (21,49).

Additional demographic details for each contributing study are given in the Supplementary Material, Table S2.

### Expression imputation

Array-based genotype data was generated for each contributing study following standard laboratory protocols and imputed to 1000 Genomes Project phase 1 or 3 reference data separately using IMPUTE2. The study-specific GWAS array data details, pre-imputation quality control filters and imputation details are given in the Supplementary Material, Table S5.

SNP imputation data for each study group was filtered prior to imputing expression to retain only SNPs with imputation info scores >0.8 and MAF > 5%. Tissue-specific imputed expression levels were obtained for each individual by applying PrediXcan imputation models (8/18/16 version) built using elastic net (α = 0.5) on the 1000 Genomes phase 1 SNP set in GTEx v6p and DGN consortium whole blood RNA sequencing data. Imputed GReX levels for PrediXcan models with *q*-value < 0.05 were carried forward for further analysis. For all reported gene-tissue pairs, we verified that they were not flagged as prone to false positives based on updates to the modeling process used in computing GTEx v7 models (list provided by Im *et al.* and publicly available on predictdb.org); one gene-tissue pair was removed.

### Phenotype definitions

Subjects taking lipid-lowering medications were excluded from lipid phenotype association analyses. For subjects from Starr County and ARIC, LDL was calculated from the Friedewald equation (50), with missing values assigned for samples with triglyceride levels >400 mg/dL. For both Mexico City studies, LDL was directly measured (21). Subjects taking medication for hypertension were excluded from blood pressure analyses. Subjects diagnosed with T2D were excluded from fasting glucose and fasting insulin levels; in Starr County individuals with incident T2D based on an oral glucose tolerance the same day were included. All other analyses included all samples with phenotype data available. Raw phenotype values for each study were regressed against age, sex and ancestry using the first and second principal components as covariates and residuals were ranked and inverse-normal transformed. Summary statistics for each phenotype are presented in the Supplementary Material, Table S2.

### Tissue/trait selections

Phenotypes were grouped into two sets of related traits—metabolic, including BMI, fasting glucose, fasting insulin, height and lipids traits (HDL, LDL, total cholesterol and triglycerides), and blood and vascular traits, including blood counts (platelets and WBC), blood pressure (DBP and SBP), clotting factor levels (factor VII and fibrinogen) and RR interval. These traits have shared underlying biology and pleiotropic loci have been reported (51,52). To limit multiple testing burden, tissues that have previously shown relevance to multiple traits in each class were selected. Predicted expressions derived from whole blood (GTEx and DGN) and the adrenal gland were used for all traits. For metabolic traits, subcutaneous and visceral omentum adipose tissues, liver, pancreas and skeletal muscle were considered (8,9,21,53). For blood and vascular traits, aortic, coronary, and tibial arteries, and atrial appendage and left ventricle of the heart were used (54–57). For height, pituitary gland expression was considered; liver expression was considered for fibrinogen and factor VII; and hypothalamus expression was considered for BMI (8,9,56).

### Association tests

Association testing of transformed phenotypes with imputed expression levels (GReX) in each study group was performed using generalized linear models in R. Ancestry-specific association results were classified by whether the gene or nearby SNP had previously been implicated in a published GWAS of the trait for each significant and suggestive gene–trait pair. Previously-observed status was initially determined by a gene start site within 250 kb of any SNP associated genome-wide with the same trait in the NHGRI-EBI catalog (58). Any potentially novel association was followed-up with searches in PubMed and Google Scholar for any prior evidence of association. To control for false discovery rate given the numerous hypothesis tests used, we employed a Benjamini–Hochberg multiple testing correction for all traits and all tissues, resulting in an experiment-wide significance threshold (59). We note that while less conservative than a Bonferroni approach to multiple test correction, this approach is likely overly conservative due to the invalid assumption of independence of tests; for example, many genes tested do not exhibit tissue-specific expression patterns, and within tissues, many genes are co-expressed.

### Trans-ancestry meta-analysis

Within each trait-tissue pairing, we meta-analyzed GReX association results for the three Hispanic studies using an inverse-variance approach, as implemented in METAL (60). We also used this approach to meta-analyze all five studies in a trans-ancestry meta-analysis. Significant and suggestive results were first selected from the trans-ancestry meta-analysis findings; any gene-trait-tissue groupings that did not reach these thresholds within the trans-ancestry results were evaluated for single ancestry association significance. QQ plots for the trans-ancestry meta-analysis results for each trait are shown in the Supplementary Material, Fig. S8, including lambda values calculated by taking the median of the chi-squared test statistics over the expected mean for each tissue (range: 0.90–1.12). Meta-analysis results were filtered to determine novelty following the same process used for ancestry-specific results.

### Additional support from Gene2Pheno

Where available, we sought additional support via replication of findings in the S-PrediXcan Gene2Pheno databases (2,61,62). S-PrediXcan utilizes SNP-level association summary statistics, in an analytical approach analogous to PrediXcan, to identify gene-level trait associations. Results derived from large, previously published GWAS (2) for several included traits (BMI, height, HDL, LDL, triglycerides, DBP and SBP) are publicly available through Gene2Pheno. Because the ARIC study contributed data to several of the large-scale association studies (accounting for at most 8.1% of the total sample size of these studies) included in Gene2Pheno, this does not represent true independent replication for all traits (noted in Table 1 and Supplementary Material, Table S1). Gene2Pheno results were not available for all tissue- and trait-specific gene associations. Many of the large GWAS in Gene2Pheno were imputed to reference datasets with smaller SNP sets, i.e. HapMap instead of 1000 Genomes; in some cases, the prediction model for a gene/tissue combination was not available in HapMap models (denoted as NR in Table 1 and Supplementary Material, Table S1). For some tissue- and trait-specific gene associations, differences in models used for Gene2Pheno resulted in model quality differences and models with high S-PrediXcan *q*-values (*q* > 0.05) were not included in Gene2Pheno. Thus, overall, we were able to attempt replication for 139 gene–trait associations.

### Gene ontology and protein–protein interaction analysis

We used DAVID Functional Annotation Tool v6.8 (63) to identify annotations, including Gene Ontology (GO) terms over-represented (Benjamini FDR < 0.20) in genes with association Benjamini–Hochberg adjusted *P* < 0.05 in the present study, stratified by trait. We used STRING (64) to search for protein–protein interactions between known and novel genes for each trait (Supplementary Material, Table S4). Scores can range between 0 and 1, with a higher score indicating more confidence that there is an interaction.

### Prediction validation by RNA sequencing

RNA sequencing data for 175 European ancestry individuals from the ARIC study were available as a test study to explore correlation of imputed expression with measured expression in whole blood (using both GTEx and DGN as reference panels). See the Supplementary Note for RNA quantification methods. GReX levels for 12 081 genes were imputed in whole blood using the PrediXcan framework. PrediXcan models with *q*-values > 0.05 (i.e. genes that were not reliably imputed) were removed, leaving a final set of 7915 genes for DGN-WB and 5632 genes for WB. The observed distribution of Pearson squared correlation coefficients was compared with that expected by chance (Supplementary Material, Fig. S1).

We imputed gene expression in the 89 African ancestry 1000 Genomes YRI (Yoruba in Ibadan, Nigeria) samples with available expression measurements (RNA-Seq) in LCLs using GTEx LCLs as the reference panel. We compared this to measured expression using previously described RNA sequencing data (65). For genes with significant Spearman correlation (*P* < 0.05) of imputed and measured gene expression, we compared the variance in expression explained with the *P*-value of the correlation. Finally, to assess the effect of differing ancestry in the training population, we compared association results for models trained in an ancestrally homogenous sample (DGN-WB) and those for models trained in GTEx, a sample for which 15% of donors are African Americans (43).

## Supplementary Material

Supplementary Data

## References

[1] Gamazon E.R., Wheeler H.E., Shah K.P., Mozaffari S.V., Aquino-Michaels K., Carroll R.J., Eyler A.E., Denny J.C., Consortium G.T., Nicolae D.L. et al. (2015) A gene-based association method for mapping traits using reference transcriptome data. *Nat. Genet.*, 47, 1091–1098. 26258848 10.1038/ng.3367 PMC4552594

[2] Barbeira A.N., Dickinson S.P., Bonazzola R., Zheng J., Wheeler H.E., Torres J.M., Torstenson E.S., Shah K.P., Garcia T., Edwards T.L. et al. (2018) Exploring the phenotypic consequences of tissue specific gene expression variation inferred from GWAS summary statistics. *Nat. Commun*., 9, 1825. 29739930 10.1038/s41467-018-03621-1 PMC5940825

[3] Visscher P.M., Medland S.E., Ferreira M.A., Morley K.I., Zhu G., Cornes B.K., Montgomery G.W. and Martin N.G. (2006) Assumption-free estimation of heritability from genome-wide identity-by-descent sharing between full siblings. *PLoS Genet.*, 2, e41. 16565746 10.1371/journal.pgen.0020041 PMC1413498

[4] Simonis-Bik A.M., Eekhoff E.M., Diamant M., Boomsma D.I., Heine R.J., Dekker J.M., Willemsen G., Leeuwen M. van and Geus E.J. de (2008) The heritability of HbA1c and fasting blood glucose in different measurement settings. *Twin Res. Hum. Genet.*, 11, 597–602. 19016616 10.1375/twin.11.6.597

[5] Tada H., Won H.H., Melander O., Yang J., Peloso G.M. and Kathiresan S. (2014) Multiple associated variants increase the heritability explained for plasma lipids and coronary artery disease. *Circ. Cardiovasc. Genet.*, 7, 583–587. 25170055 10.1161/CIRCGENETICS.113.000420 PMC4341828

[6] Marouli E., Graff M., Medina-Gomez C., Lo K.S., Wood A.R., Kjaer T.R., Fine R.S., Lu Y., Schurmann C., Highland H.M. et al. (2017) Rare and low-frequency coding variants alter human adult height. *Nature*, 542, 186–190. 28146470 10.1038/nature21039 PMC5302847

[7] Shungin D., Winkler T.W., Croteau-Chonka D.C., Ferreira T., Locke A.E., Magi R., Strawbridge R.J., Pers T.H., Fischer K., Justice A.E. et al. (2015) New genetic loci link adipose and insulin biology to body fat distribution. *Nature*, 518, 187–196. 25673412 10.1038/nature14132 PMC4338562

[8] Locke A.E., Kahali B., Berndt S.I., Justice A.E., Pers T.H., Day F.R., Powell C., Vedantam S., Buchkovich M.L., Yang J. et al. (2015) Genetic studies of body mass index yield new insights for obesity biology. *Nature*, 518, 197–206. 25673413 10.1038/nature14177 PMC4382211

[9] Wood A.R., Esko T., Yang J., Vedantam S., Pers T.H., Gustafsson S., Chu A.Y., Estrada K., Luan J., Kutalik Z. et al. (2014) Defining the role of common variation in the genomic and biological architecture of adult human height. *Nat. Genet.*, 46, 1173–1186. 25282103 10.1038/ng.3097 PMC4250049

[10] Turcot V., Lu Y., Highland H.M., Schurmann C., Justice A.E., Fine R.S., Bradfield J.P., Esko T., Giri A., Graff M. et al. (2018) Protein-altering variants associated with body mass index implicate pathways that control energy intake and expenditure in obesity. *Nat. Genet.*, 50, 26–41. 29273807 10.1038/s41588-017-0011-x PMC5945951

[11] Willer C.J., Schmidt E.M., Sengupta S., Peloso G.M., Gustafsson S., Kanoni S., Ganna A., Chen J., Buchkovich M.L., Mora S. et al. (2013) Discovery and refinement of loci associated with lipid levels. *Nat. Genet.*, 45, 1274–1283. 24097068 10.1038/ng.2797 PMC3838666

[12] Koh W., Pan W., Gawad C., Fan H.C., Kerchner G.A., Wyss-Coray T., Blumenfeld Y.J., El-Sayed Y.Y. and Quake S.R. (2014) Noninvasive in vivo monitoring of tissue-specific global gene expression in humans. *Proc. Nat. Acad. Sci. USA*, 111, 7361–7366. 24799715 10.1073/pnas.1405528111 PMC4034220

[13] Cheung V.G. and Spielman R.S. (2009) Genetics of human gene expression: mapping DNA variants that influence gene expression. *Nat. Rev. Genet.*, 10, 595–604. 19636342 10.1038/nrg2630 PMC2989458

[14] Consortium G. (2015) Human genomics. The Genotype-Tissue Expression (GTEx) pilot analysis: multitissue gene regulation in humans. *Science*, 348, 648–660. 25954001 10.1126/science.1262110 PMC4547484

[15] Xu J., Shao Z., Glass K., Bauer D.E., Pinello L., Van Handel B., Hou S., Stamatoyannopoulos J.A., Mikkola H.K., Yuan G.C. et al. (2012) Combinatorial assembly of developmental stage-specific enhancers controls gene expression programs during human erythropoiesis. *Dev. Cell*, 23, 796–811. 23041383 10.1016/j.devcel.2012.09.003 PMC3477283

[16] Cortessis V.K., Thomas D.C., Levine A.J., Breton C.V., Mack T.M., Siegmund K.D., Haile R.W. and Laird P.W. (2012) Environmental epigenetics: prospects for studying epigenetic mediation of exposure-response relationships. *Hum. Genet.*, 131, 1565–1589. 22740325 10.1007/s00439-012-1189-8 PMC3432200

[17] Sanyal A., Lajoie B.R., Jain G. and Dekker J. (2012) The long-range interaction landscape of gene promoters. *Nature*, 489, 109–113. 22955621 10.1038/nature11279 PMC3555147

[18] Berchtold N.C., Sabbagh M.N., Beach T.G., Kim R.C., Cribbs D.H. and Cotman C.W. (2014) Brain gene expression patterns differentiate mild cognitive impairment from normal aged and Alzheimer's disease. *Neurobiol. Aging*, 35, 1961–1972. 24786631 10.1016/j.neurobiolaging.2014.03.031 PMC4067010

[19] Herazo-Maya J.D., Noth I., Duncan S.R., Kim S., Ma S.F., Tseng G.C., Feingold E., Juan-Guardela B.M., Richards T.J., Lussier Y. et al. (2013) Peripheral blood mononuclear cell gene expression profiles predict poor outcome in idiopathic pulmonary fibrosis. *Sci. Transl. Med.*, 5, 205ra136. 10.1126/scitranslmed.3005964 PMC4175518 24089408

[20] Nicolae D.L., Gamazon E., Zhang W., Duan S., Dolan M.E. and Cox N.J. (2010) Trait-associated SNPs are more likely to be eQTLs: annotation to enhance discovery from GWAS. *PLoS Genet.*, 6, 1 0 e1000888. 10.1371/journal.pgen.1000888 PMC2848547 20369019

[21] Below J.E., Parra E.J., Gamazon E.R., Torres J., Krithika S., Candille S., Lu Y., Manichakul A., Peralta-Romero J., Duan Q. et al. (2016) Meta-analysis of lipid-traits in Hispanics identifies novel loci, population-specific effects, and tissue-specific enrichment of eQTLs. *Sci. Rep.*, 6, 19429. 26780889 10.1038/srep19429 PMC4726092

[22] Schizophrenia Working Group of the Psychiatric Genomics Consortium (2014) Biological insights from 108 schizophrenia-associated genetic loci. *Nature*, 511, 421–427. 25056061 10.1038/nature13595 PMC4112379

[23] Torres J.M., Gamazon E.R., Parra E.J., Below J.E., Valladares-Salgado A., Wacher N., Cruz M., Hanis C.L. and Cox N.J. (2014) Cross-tissue and tissue-specific eQTLs: partitioning the heritability of a complex trait. *Am. J. Hum. Genet.*, 95, 521–534. 25439722 10.1016/j.ajhg.2014.10.001 PMC4225593

[24] Huang C.C., Fornage M., Lloyd-Jones D.M., Wei G.S., Boerwinkle E. and Liu K. (2009) Longitudinal association of PCSK9 sequence variations with low-density lipoprotein cholesterol levels: the Coronary Artery Risk Development in Young Adults Study. *Circ. Cardiovasc. Genet.*, 2, 354–361. 20031607 10.1161/CIRCGENETICS.108.828467 PMC2810147

[25] Gusev A., Ko A., Shi H., Bhatia G., Chung W., Penninx B.W., Jansen R., Geus E.J. de, Boomsma D.I., Wright F.A. et al. (2016) Integrative approaches for large-scale transcriptome-wide association studies. *Nat. Genet.*, 48, 245–252. 26854917 10.1038/ng.3506 PMC4767558

[26] Friedman J., Hastie T. and Tibshirani R. (2010) Regularization paths for generalized linear models via coordinate descent. *J. Stat. Softw.*, 33,–22. PMC2929880 20808728

[27] Wheeler H.E., Shah K.P., Brenner J., Garcia T., Aquino-Michaels K., Consortium G.T., Cox N.J., Nicolae D.L. and Im H.K. (2016) Survey of the heritability and sparse architecture of gene expression traits across human tissues. *PLoS Genet.*, 12, e1006423. 27835642 10.1371/journal.pgen.1006423 PMC5106030

[28] Fish A.E., Capra J.A. and Bush W.S. (2016) Are interactions between cis-regulatory variants evidence for biological epistasis or statistical artifacts? *Am. J. Hum. Genet.*, 99, 817–830. 27640306 10.1016/j.ajhg.2016.07.022 PMC5065654

[29] Carithers L.J., Ardlie K., Barcus M., Branton P.A., Britton A., Buia S.A., Compton C.C., Deluca D.S., Peter-Demchok J., Gelfand E.T. et al. (2015) A novel approach to high-quality postmortem tissue procurement: The GTEx Project. *Biopreserv. Biobank*, 13, 311–319. 26484571 10.1089/bio.2015.0032 PMC4675181

[30] Carithers L.J. and Moore H.M. (2015) The Genotype-Tissue Expression (GTEx) Project. *Biopreserv. Biobank*, 13, 307–308. 26484569 10.1089/bio.2015.29031.hmm PMC4692118

[31] Battle A., Mostafavi S., Zhu X., Potash J.B., Weissman M.M., McCormick C., Haudenschild C.D., Beckman K.B., Shi J., Mei R. et al. (2014) Characterizing the genetic basis of transcriptome diversity through RNA-sequencing of 922 individuals. *Genome Res.*, 24, 14–24. 24092820 10.1101/gr.155192.113 PMC3875855

[32] Manor O. and Segal E. (2013) Robust prediction of expression differences among human individuals using only genotype information. *PLoS Genet.*, 9, e1003396. 23555302 10.1371/journal.pgen.1003396 PMC3610805

[33] Reich D., Nalls M.A., Kao W.H., Akylbekova E.L., Tandon A., Patterson N., Mullikin J., Hsueh W.C., Cheng C.Y., Coresh J. et al. (2009) Reduced neutrophil count in people of African descent is due to a regulatory variant in the Duffy antigen receptor for chemokines gene. *PLoS Genet.*, 5, e1000360. 19180233 10.1371/journal.pgen.1000360 PMC2628742

[34] Smith N.L., Chen M.H., Dehghan A., Strachan D.P., Basu S., Soranzo N., Hayward C., Rudan I., Sabater-Lleal M., Bis J.C. et al. (2010) Novel associations of multiple genetic loci with plasma levels of factor VII, factor VIII, and von Willebrand factor: The CHARGE (Cohorts for Heart and Aging Research in Genome Epidemiology) Consortium. *Circulation*, 121, 1382–1392. 20231535 10.1161/CIRCULATIONAHA.109.869156 PMC2861278

[35] Sethi M.K., Buettner F.F., Krylov V.B., Takeuchi H., Nifantiev N.E., Haltiwanger R.S., Gerardy-Schahn R. and Bakker H. (2010) Identification of glycosyltransferase 8 family members as xylosyltransferases acting on O-glucosylated notch epidermal growth factor repeats. J. Biol. Chem., 285, 1582–1586. 19940119 10.1074/jbc.C109.065409 PMC2804315

[36] Bjoern S., Foster D.C., Thim L., Wiberg F.C., Christensen M., Komiyama Y., Pedersen A.H. and Kisiel W. (1991) Human plasma and recombinant factor VII. Characterization of O-glycosylations at serine residues 52 and 60 and effects of site-directed mutagenesis of serine 52 to alanine. *J. Biol. Chem.*, 266, 11051–11057. 1904059

[37] Ren Q., Barber H.K., Crawford G.L., Karim Z.A., Zhao C., Choi W., Wang C.C., Hong W. and Whiteheart S.W. (2007) Endobrevin/VAMP-8 is the primary v-SNARE for the platelet release reaction. *Mol. Biol. Cell*, 18, 24–33. 17065550 10.1091/mbc.E06-09-0785 PMC1751319

[38] Heijnen H. and Sluijs P. van der (2015) Platelet secretory behaviour: as diverse as the granules ... or not? *J. Thromb. Haemost.*, 13, 2141–2151. 26391322 10.1111/jth.13147

[39] Chambers J.C., Loh M., Lehne B., Drong A., Kriebel J., Motta V., Wahl S., Elliott H.R., Rota F., Scott W.R. et al. (2015) Epigenome-wide association of DNA methylation markers in peripheral blood from Indian Asians and Europeans with incident type 2 diabetes: a nested case-control study. *Lancet Diabetes Endocrinol.*, 3, 526–534. 26095709 10.1016/S2213-8587(15)00127-8 PMC4724884

[40] Dayeh T., Tuomi T., Almgren P., Perfilyev A., Jansson P.A., Mello V.D. de, Pihlajamaki J., Vaag A., Groop L., Nilsson E. et al. (2016) DNA methylation of loci within ABCG1 and PHOSPHO1 in blood DNA is associated with future type 2 diabetes risk. *Epigenetics*, 11, 482–488. 27148772 10.1080/15592294.2016.1178418 PMC4939923

[41] Kurano M., Tsukamoto K., Kamitsuji S., Kamatani N., Hara M., Ishikawa T., Kim B.J., Moon S., Jin Kim Y. and Teramoto T. (2016) Genome-wide association study of serum lipids confirms previously reported associations as well as new associations of common SNPs within PCSK7 gene with triglyceride. *J. Hum. Genet.*, 61, 427–433. 26763881 10.1038/jhg.2015.170

[42] Peloso G.M., Auer P.L., Bis J.C., Voorman A., Morrison A.C., Stitziel N.O., Brody J.A., Khetarpal S.A., Crosby J.R., Fornage M. et al. (2014) Association of low-frequency and rare coding-sequence variants with blood lipids and coronary heart disease in 56,000 whites and blacks. *Am. J. Hum. Genet.*, 94, 223–232. 24507774 10.1016/j.ajhg.2014.01.009 PMC3928662

[43] GTEx Consortium, Laboratory, Data Analysis & Coordinating Center—Analysis Working Group, Statistical Methods groups–Analysis Working Group, Enhancing GTEx groups, N.I.H. Common Fund, NIH/NCI, NIH/NHGRI, NIH/NIMH, NIH/NIDA, Biospecimen Collection Source Site—NDRI *et al.* (2017) Genetic effects on gene expression across human tissues. *Nature*, 550, 204–213. 29022597 10.1038/nature24277 PMC5776756

[44] Consortium G.T.E. (2015) The Genotype-Tissue Expression (GTEx) pilot analysis: multitissue gene regulation in humans. *Science*, 348, 648–660. 25954001 10.1126/science.1262110 PMC4547484

[45] Monajemi H., Fontijn R.D., Pannekoek H. and Horrevoets A.J. (2002) The apolipoprotein L gene cluster has emerged recently in evolution and is expressed in human vascular tissue. *Genomics*, 79, 539–546. 11944986 10.1006/geno.2002.6729

[46] Page N.M., Butlin D.J., Lomthaisong K. and Lowry P.J. (2001) The human apolipoprotein L gene cluster: identification, classification, and sites of distribution. *Genomics*, 74, 71–78. 11374903 10.1006/geno.2001.6534

[47] Investigators T.A.R.I.C. (1989) The Atherosclerosis Risk in Communities (ARIC) Study: design and objectives. The ARIC investigators. *Am. J. Epidemiol.*, 129, 687–702. 2646917

[48] Hanis C.L., Redline S., Cade B.E., Bell G.I., Cox N.J., Below J.E., Brown E.L. and Aguilar D. (2016) Beyond type 2 diabetes, obesity and hypertension: an axis including sleep apnea, left ventricular hypertrophy, endothelial dysfunction, and aortic stiffness among Mexican Americans in Starr County, Texas. *Cardiovasc. Diabetol.*, 15, 86. 27266869 10.1186/s12933-016-0405-6 PMC4897940

[49] Parra E.J., Below J.E., Krithika S., Valladares A., Barta J.L., Cox N.J., Hanis C.L., Wacher N., Garcia-Mena J., Hu P. et al. (2011) Genome-wide association study of type 2 diabetes in a sample from Mexico City and a meta-analysis of a Mexican-American sample from Starr County, Texas. *Diabetologia*, 54, 2038–2046. 21573907 10.1007/s00125-011-2172-y PMC3818640

[50] Friedewald W.T., Levy R.I. and Fredrickson D.S. (1972) Estimation of the concentration of low-density lipoprotein cholesterol in plasma, without use of the preparative ultracentrifuge. *Clin. Chem.*, 18, 499–502. 4337382

[51] Gratten J. and Visscher P.M. (2016) Genetic pleiotropy in complex traits and diseases: implications for genomic medicine. *Genome Med.*, 8, 78. 27435222 10.1186/s13073-016-0332-x PMC4952057

[52] Solovieff N., Cotsapas C., Lee P.H., Purcell S.M. and Smoller J.W. (2013) Pleiotropy in complex traits: challenges and strategies. *Nat. Rev. Genet.*, 14, 483–495. 23752797 10.1038/nrg3461 PMC4104202

[53] Spracklen C.N., Shi J., Vadlamudi S., Wu Y., Zou M., Raulerson C.K., Davis J.P., Zeynalzadeh M., Jackson K., Yuan W. et al. (2018) Identification and functional analysis of glycemic trait loci in the China Health and Nutrition Survey. *PLoS Genet.*, 14, e1007275. 29621232 10.1371/journal.pgen.1007275 PMC5886383

[54] Astle W.J., Elding H., Jiang T., Allen D., Ruklisa D., Mann A.L., Mead D., Bouman H., Riveros-McKay F., Kostadima M.A. et al. (2016) The allelic landscape of human blood cell trait variation and links to common complex disease. *Cell*, 167, 1415–1429. 27863252 10.1016/j.cell.2016.10.042 PMC5300907

[55] Surendran P., Drenos F., Young R., Warren H., Cook J.P., Manning A.K., Grarup N., Sim X., Barnes D.R., Witkowska K. et al. (2016) Trans-ancestry meta-analyses identify rare and common variants associated with blood pressure and hypertension. *Nat. Genet.*, 48, 1151–1161. 27618447 10.1038/ng.3654 PMC5056636

[56] Vries P.S. de, Chasman D.I., Sabater-Lleal M., Chen M.H., Huffman J.E., Steri M., Tang W., Teumer A., Marioni R.E., Grossmann V. et al. (2016) A meta-analysis of 120,246 individuals identifies 18 new loci for fibrinogen concentration. *Hum. Mol. Genet.*, 25, 358–370. 26561523 10.1093/hmg/ddv454 PMC4715256

[57] Ramirez J., Duijvenboden S.V., Ntalla I., Mifsud B., Warren H.R., Tzanis E., Orini M., Tinker A., Lambiase P.D. and Munroe P.B. (2018) Thirty loci identified for heart rate response to exercise and recovery implicate autonomic nervous system. *Nat. Commun.*, 9, 1947. 29769521 10.1038/s41467-018-04148-1 PMC5955978

[58] MacArthur J., Bowler E., Cerezo M., Gil L., Hall P., Hastings E., Junkins H., McMahon A., Milano A., Morales J. et al. (2017) The new NHGRI-EBI Catalog of published genome-wide association studies (GWAS Catalog). *Nucleic Acids Res.*, 45, D896–D901. 27899670 10.1093/nar/gkw1133 PMC5210590

[59] Benjamini Y. and Hochberg Y. (1995) Controlling the false discovery rate: a practical and powerful approach to multiple testing. *J. R. Stat. Soc. Ser. B Methodol.*, 57, 289–300.

[60] Willer C.J., Li Y. and Abecasis G.R. (2010) METAL: fast and efficient meta-analysis of genomewide association scans. *Bioinformatics*, 26, 2190–2191. 20616382 10.1093/bioinformatics/btq340 PMC2922887

[61] So H.C., Chau C.K., Chiu W.T., Ho K.S., Lo C.P., Yim S.H. and Sham P.C. (2017) Analysis of genome-wide association data highlights candidates for drug repositioning in psychiatry. *Nat. Neurosci.*, 20, 1342–1349. 28805813 10.1038/nn.4618

[62] Warrier V., Grasby K.L., Uzefovsky F., Toro R., Smith P., Chakrabarti B., Khadake J., Mawbey-Adamson E., Litterman N., Hottenga J.J. et al. (2018) Genome-wide meta-analysis of cognitive empathy: heritability, and correlates with sex, neuropsychiatric conditions and cognition. *Mol. Psychiatry*, 23, 1402–1409. 28584286 10.1038/mp.2017.122 PMC5656177

[63] Huang d., W., Sherman B.T. and Lempicki R.A. (2009) Bioinformatics enrichment tools: paths toward the comprehensive functional analysis of large gene lists. *Nucleic Acids Res.*, 37,–13. 10.1093/nar/gkn923 PMC2615629 19033363

[64] Szklarczyk D., Franceschini A., Wyder S., Forslund K., Heller D., Huerta-Cepas J., Simonovic M., Roth A., Santos A., Tsafou K.P. et al. (2015) STRING v10: protein-protein interaction networks, integrated over the tree of life. *Nucleic Acids Res.*, 43, D447–D452. 25352553 10.1093/nar/gku1003 PMC4383874

[65] Lappalainen T., Sammeth M., Friedlander M.R., t Hoen P.A., Monlong J., Rivas M.A., Gonzalez-Porta M., Kurbatova N., Griebel T., Ferreira P.G. et al. (2013) Transcriptome and genome sequencing uncovers functional variation in humans. *Nature*, 501, 506–511. 24037378 10.1038/nature12531 PMC3918453

