# Supplementary material for: Functionally oriented analysis of cardiometabolic traits in a trans-ethnic sample
Source: Hum Mol Genet. 2019 Jan 8;28(7):1212–24. doi: 10.1093/hmg/ddy435 (PMC6423424; doi:10.1093/hmg/ddy435)
Supplement: Supplementary Data [file ddy435_supp.zip › Supplementary_Figure_Legends.docx]

**Supplementary Figure Legends**

**Supplementary Figure 1:** ARIC RNA sequencing comparisons

a) QQ plot for expected versus observed predictive R^2^, i.e., squared correlation coefficient of the ARIC RNA sequencing data with estimated PrediXcan GReX. Note a significant deviation of observed R^2^ from the distribution of R^2^ expected by chance.

b) In order of increasing h^2^, each gene’s h^2^, shown in black with 95% confidence interval shown in grey, is compared with its R^2^, shown in red, indicating a trend of increased R^2^ with higher h^2^ genes.

**Supplementary Figure 2:** Comparison of Z scores in trans-ethnic study for models developed in DGN whole blood and GTEx whole blood

Comparison of Z scores in our trans-ethnic data for each gene resulting from imputing expression using models developed in a mostly European-ancestry reference dataset (DGN whole blood) with those from models developed in a more ancestrally diverse dataset (GTEx whole blood).

**Supplementary Figure 3:** Comparison of explained variance in African- and European-ancestry samples

Distribution of explained variance in the directly measured gene expression using the genetically determined expression for YRI (orange) and ARIC (blue). Note that for a large number of genes, the African YRI samples could be imputed with the same performance quality as the European-ancestry ARIC, but that ARIC had a thicker tail-end distribution (for example, more genes with R^2^>0.10) and higher explained variance given the shared (European) ancestry with the imputation training set.

**Supplementary Figure 4:** Comparison of variance explained (R^2^) and *p*-value of the correlation in YRI LCLs

For genes with significant (p<0.05) Spearman correlation between imputed expression and measured expression in YRI LCLs, variance explained by predicted expression is plotted against the *p*-value of the correlation of predicted and measured expression.

**Supplementary Figure 5:** Flow chart of methods and results classification

Workflow for all analysis methods, including filtering and classification of results.

**Supplementary Figure 6:** Quantile-quantile plots for each trait-tissue combination separated by proximity to GWAS-associated

Comparison of expected and observed distribution of *p*-values of GReX-trait association for each gene in each assessed tissue. Genes within 250kb of GWAS variants are colored blue, genes outside of these regions are red, and all genes combined are in black.

**Supplementary Figure 7:** Z-scores for novel gene GReX-trait association across relevant tissues for a) BMI, b) fasting glucose, c) fasting insulin, d) height, e) HDL cholesterol, f) LDL cholesterol.

g) total cholesterol, h) triglycerides, i) platelet count, j) WBC count, k) DBP, l) SBP, m) factor VII, n) fibrinogen, and o) RR interval.

Comparison of Z-scores across all assessed tissues for each trait. Size of the point represents magnitude, color indicates direction of effect (blue points are positively associated with the trait, red points are negatively associated with the trait), and shade indicates the R^2^ from the prediction model. Missing points indicate that there is no model available for the given gene in that tissue.

**Supplementary Figure 8:** Quantile-quantile plots of results for each trait

Quantile-quantile plots comparing expected and observed distribution of *p*-values for all tissues, separated by trait: a) BMI, b) fasting glucose, c) fasting insulin, d) height, e) HDL cholesterol, f) LDL cholesterol, g) total cholesterol, h) triglycerides i) platelet count j) WBC count k) DPB, l) SBP, m) factor VII, n) fibrinogen, and o) RR interval. Tissues are indicated by color legend on each plot.
