# Supplementary material for: Functionally oriented analysis of cardiometabolic traits in a trans-ethnic sample
Source: Hum Mol Genet. 2019 Jan 8;28(7):1212–24. doi: 10.1093/hmg/ddy435 (PMC6423424; doi:10.1093/hmg/ddy435)

Supplementary Figure 1: ARIC RNA sequencing comparisons

a)


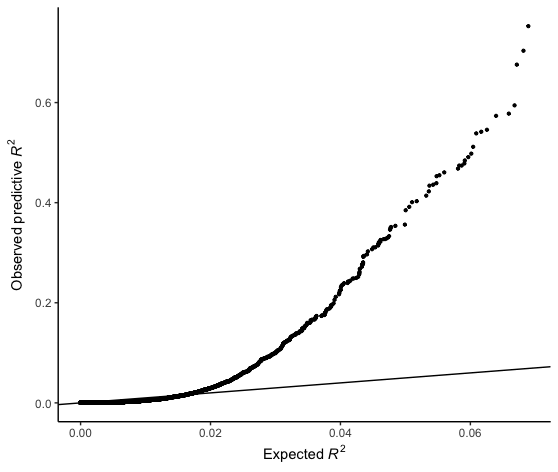


b)


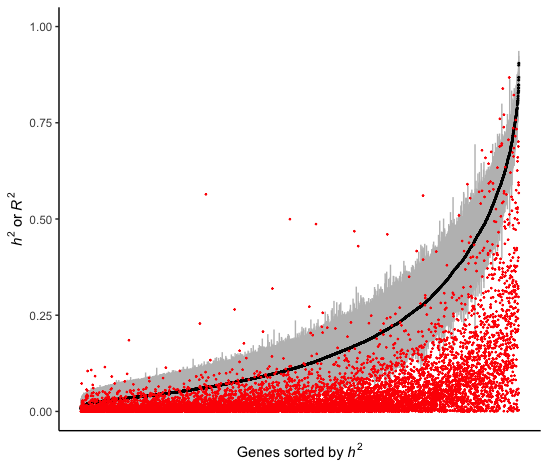


Supplementary Figure 2: Comparison of Z scores in trans-ethnic study for models developed in DGN whole blood and GTEx whole blood

a) Body mass index


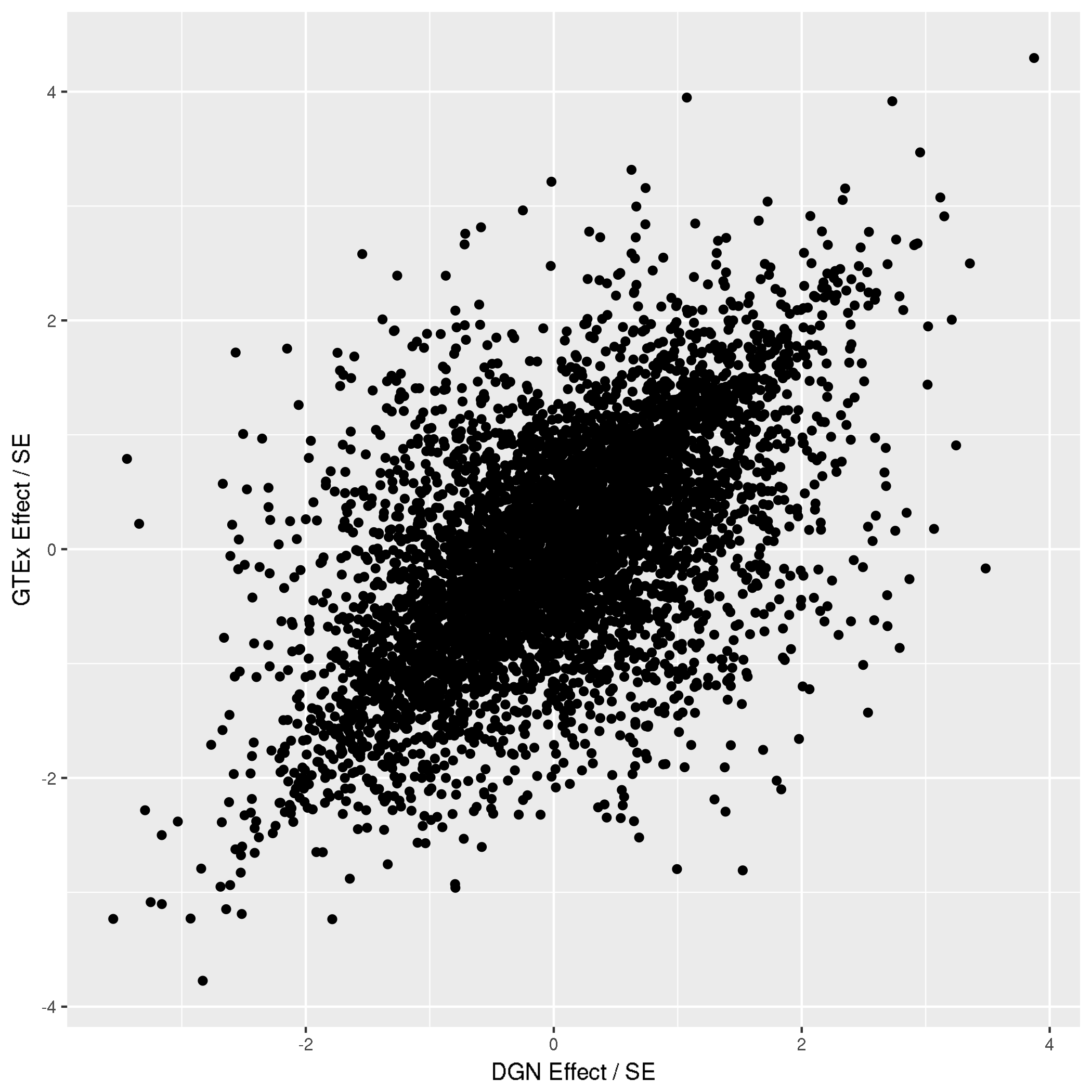


b) Diastolic blood pressure


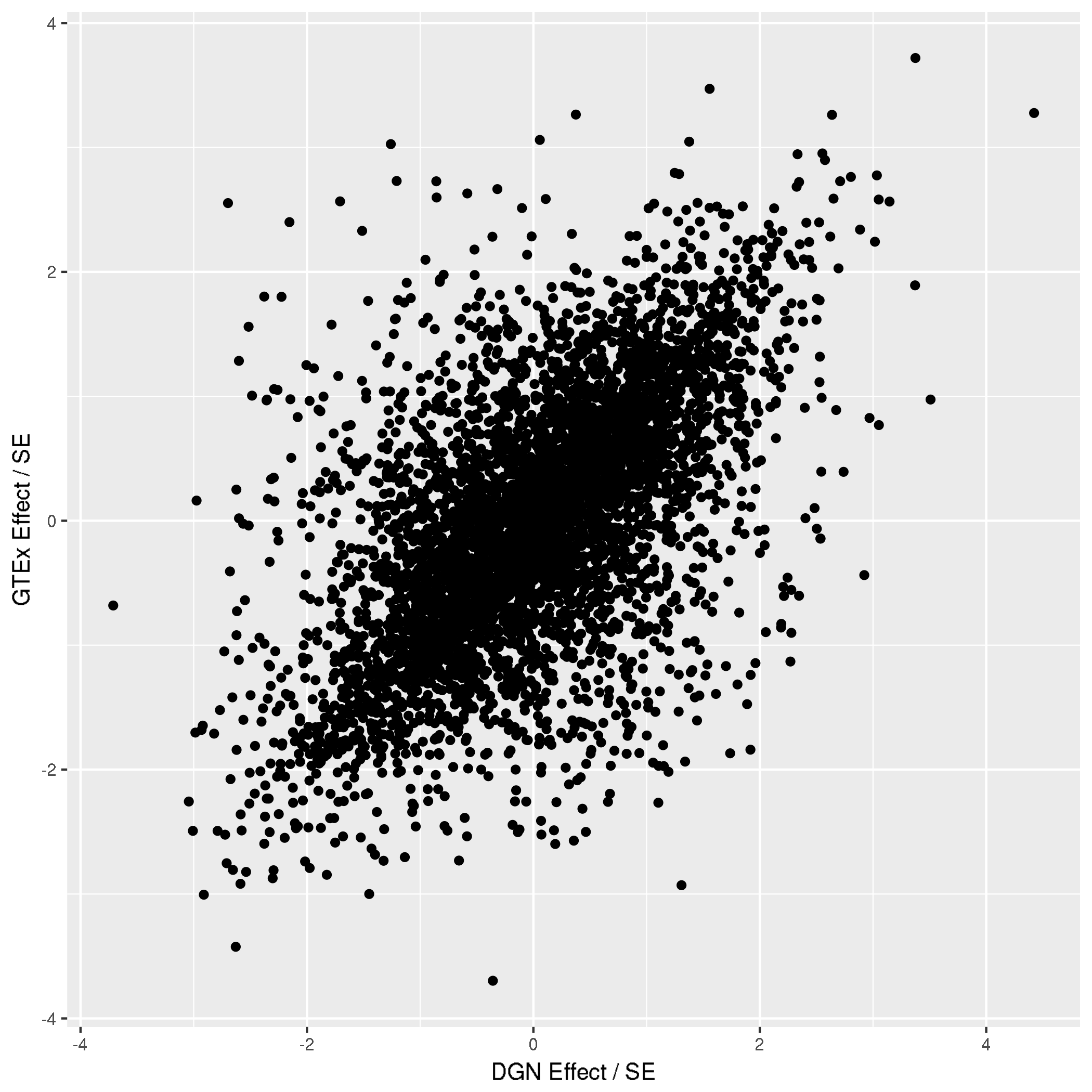


c) Fasting insulin


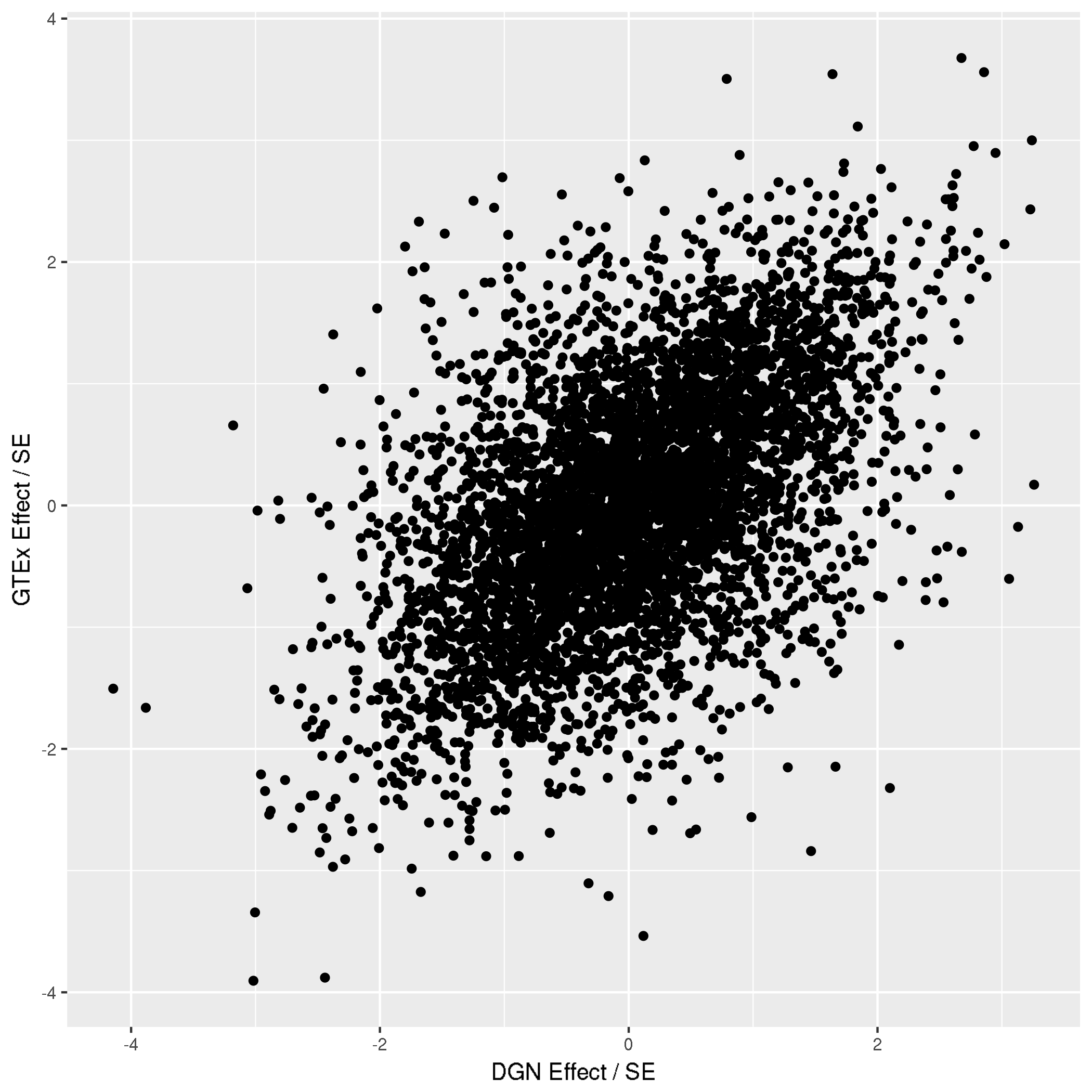


d) Fibrinogen


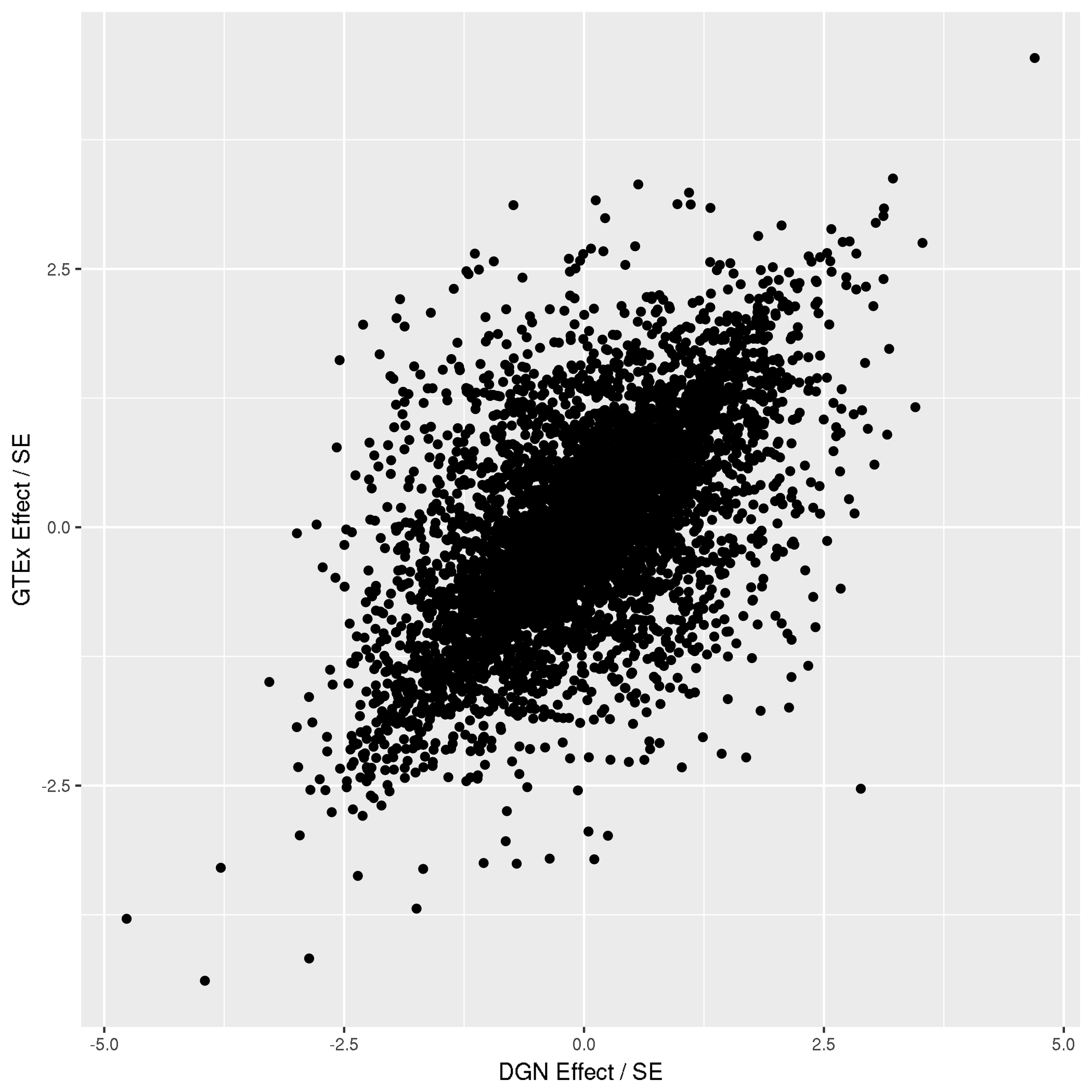


e) Factor VII
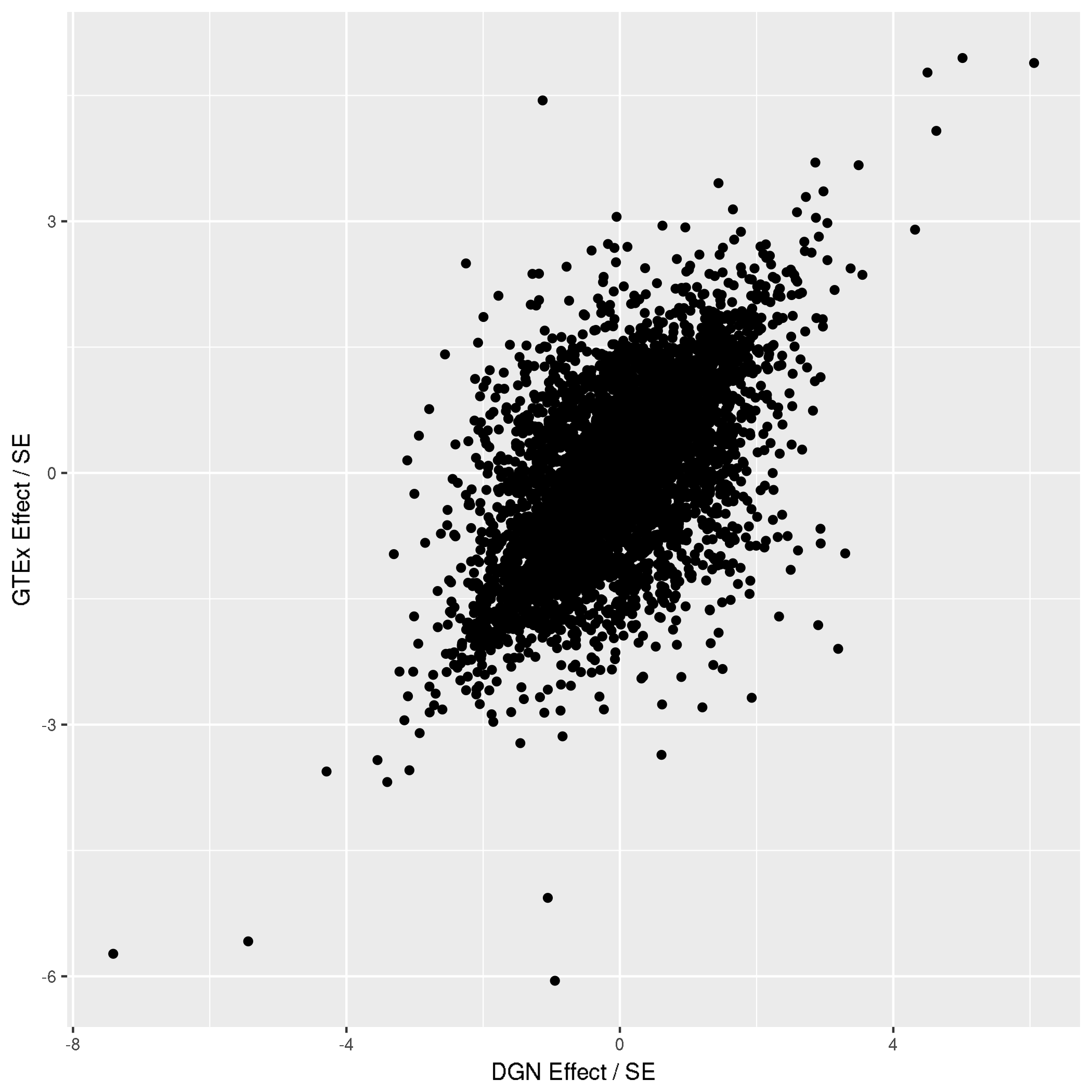


f) Fasting glucose


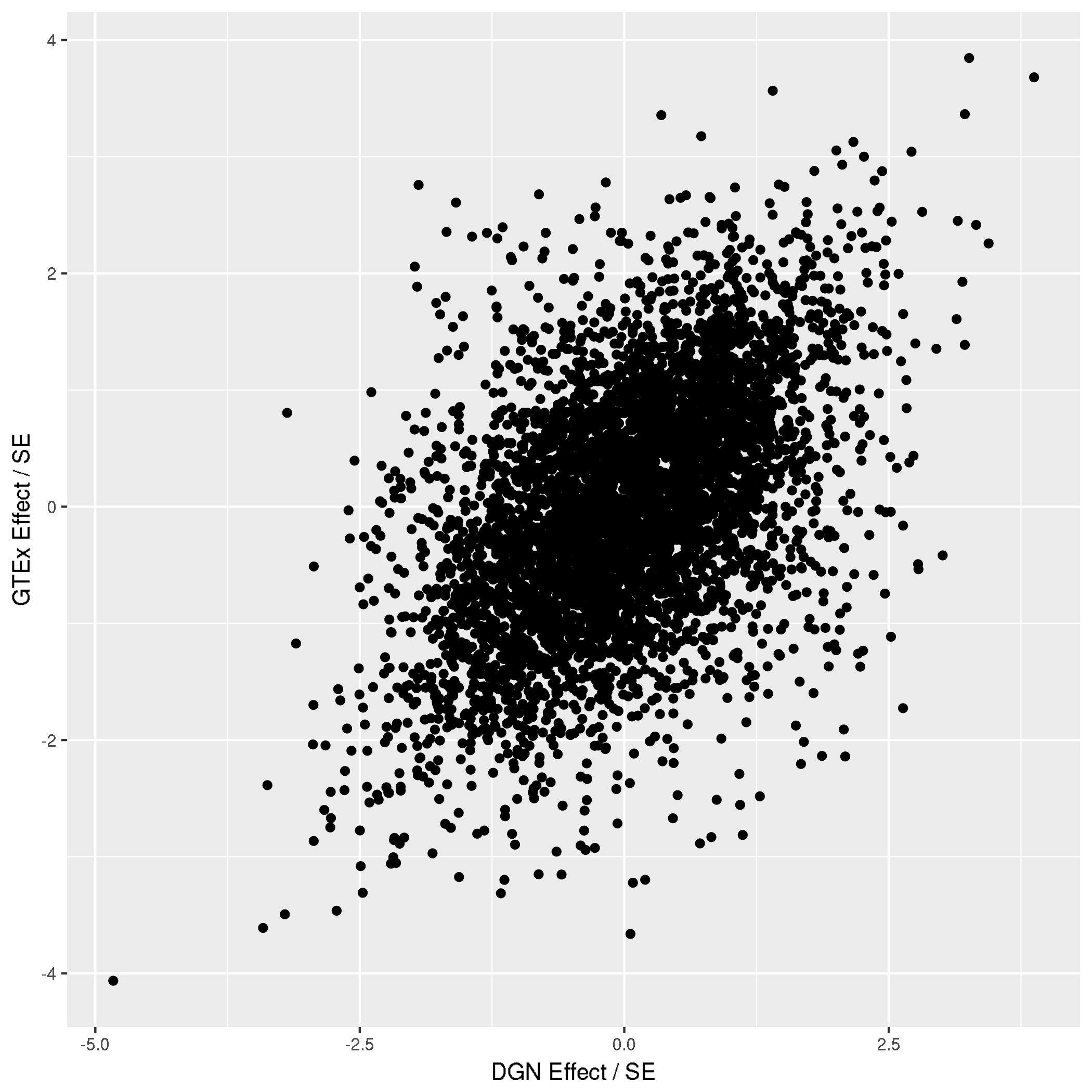


g) HDL cholesterol


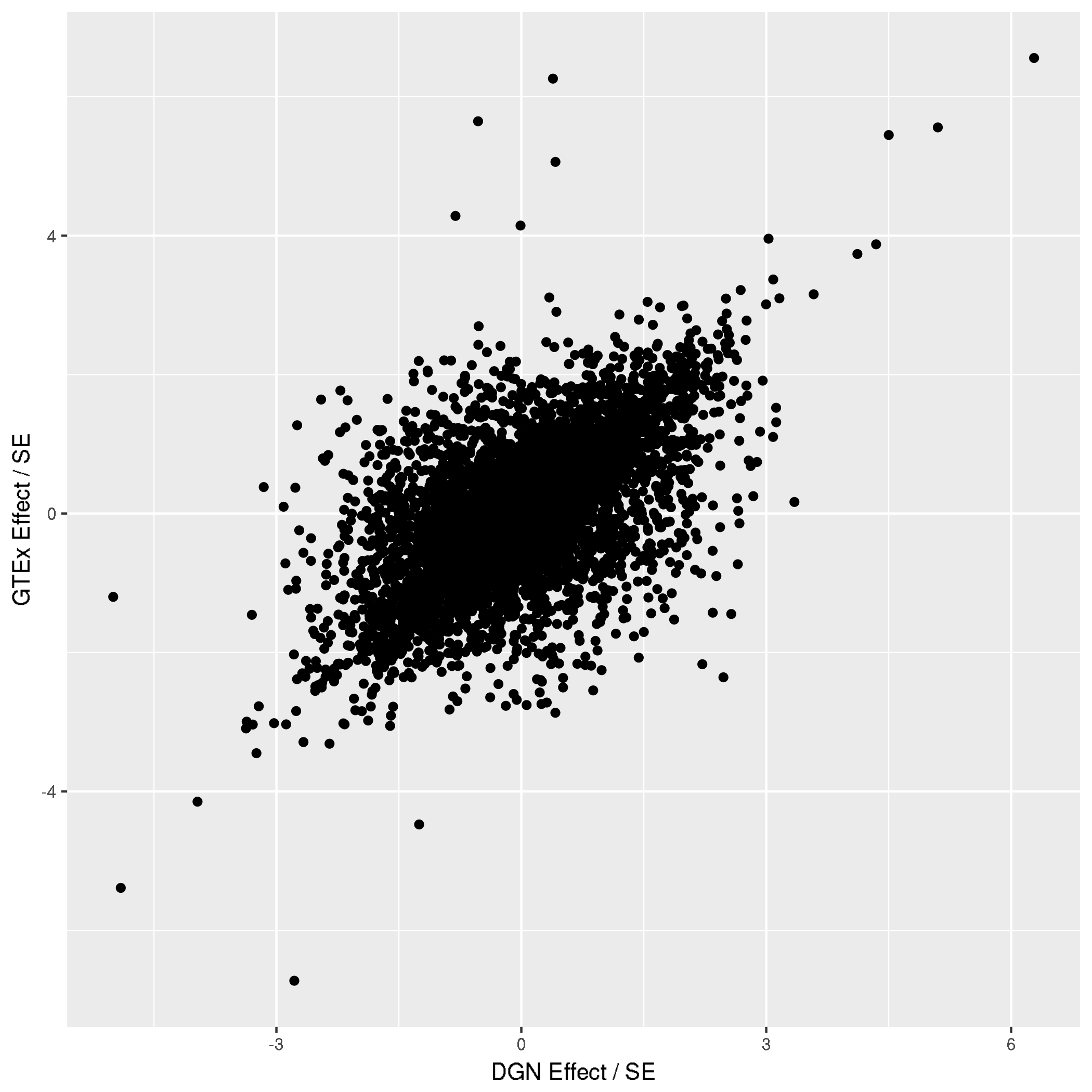


h) Height


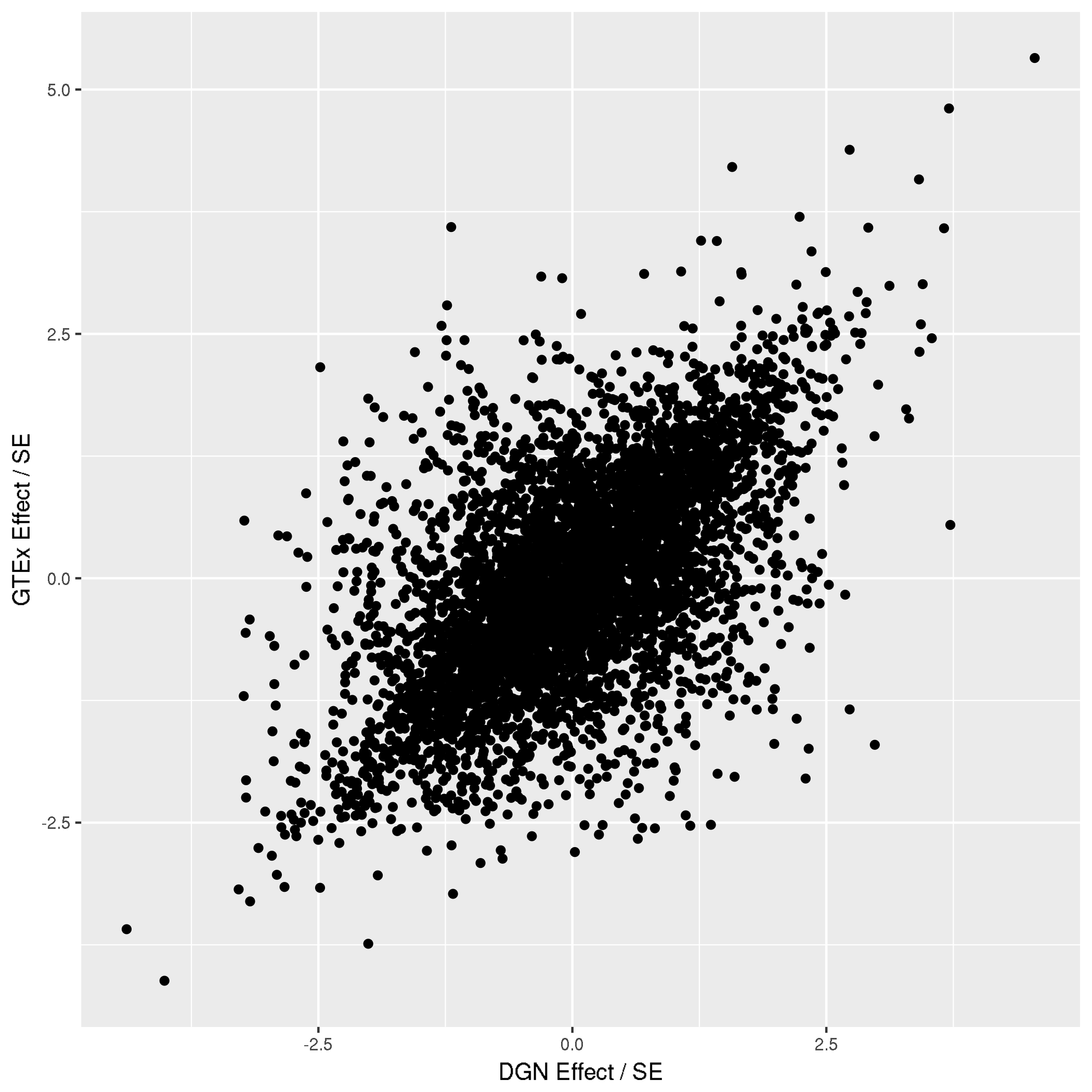


i) LDL cholesterol


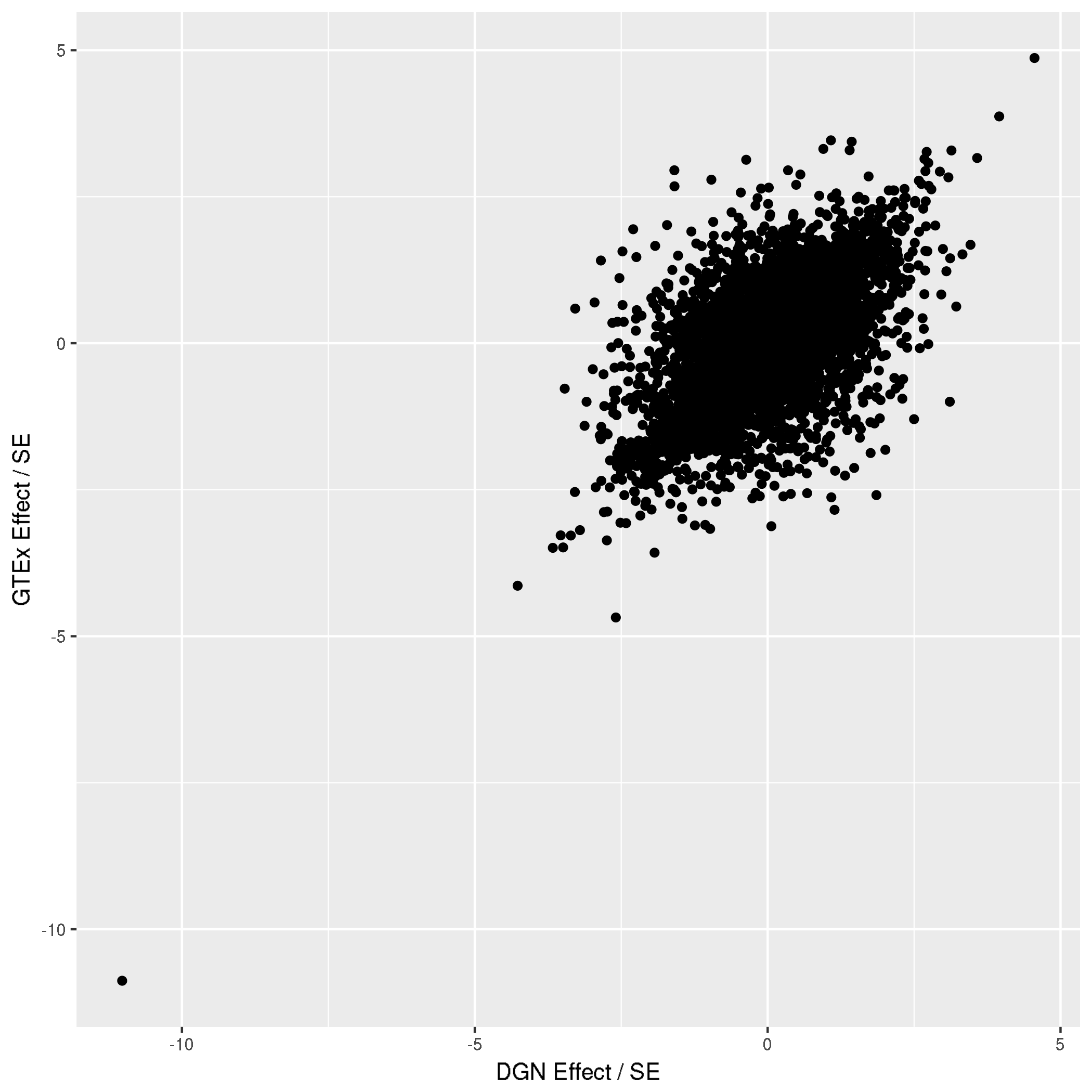


j) Platelet count


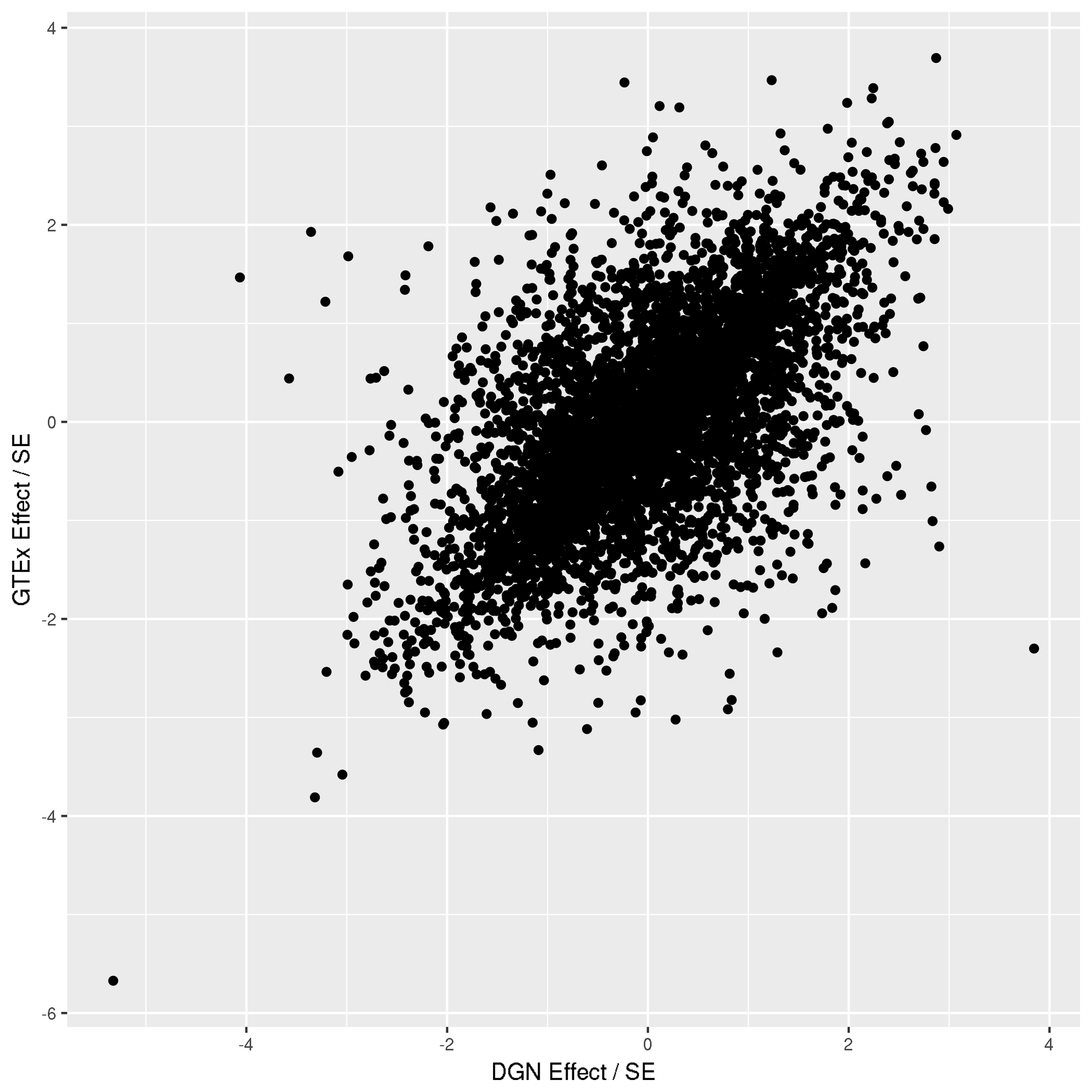


k) RR interval


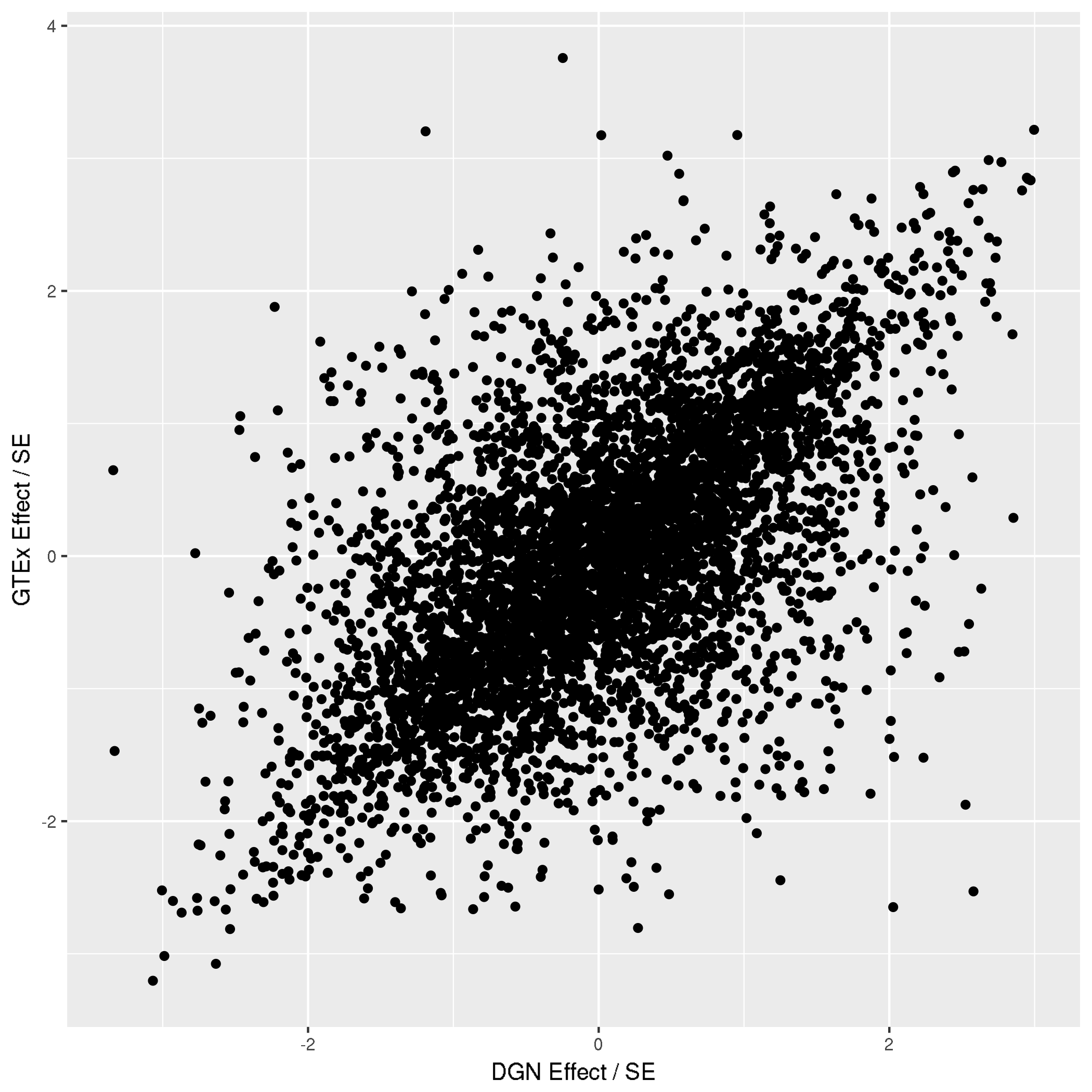


l) Systolic blood pressure


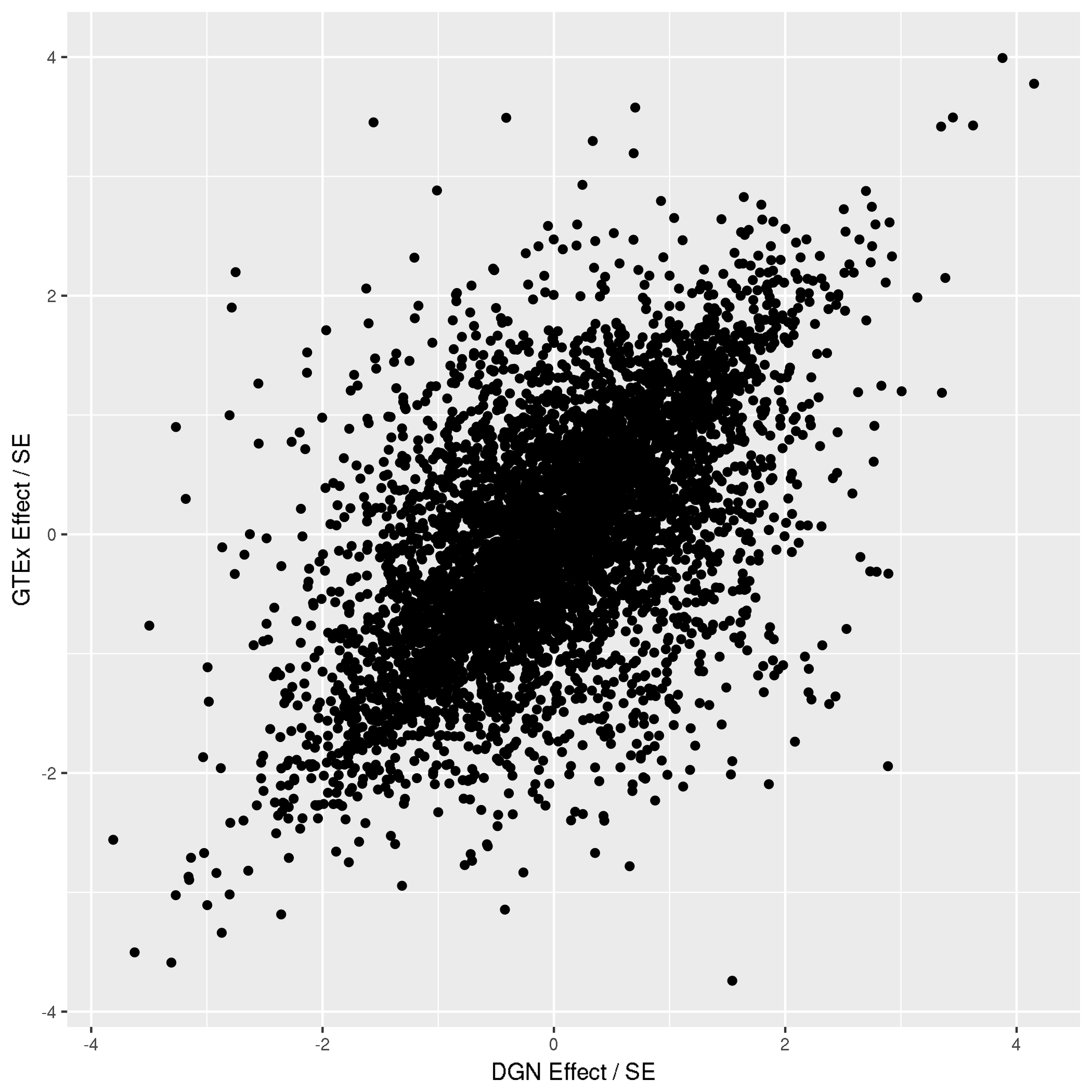


m) Total cholesterol


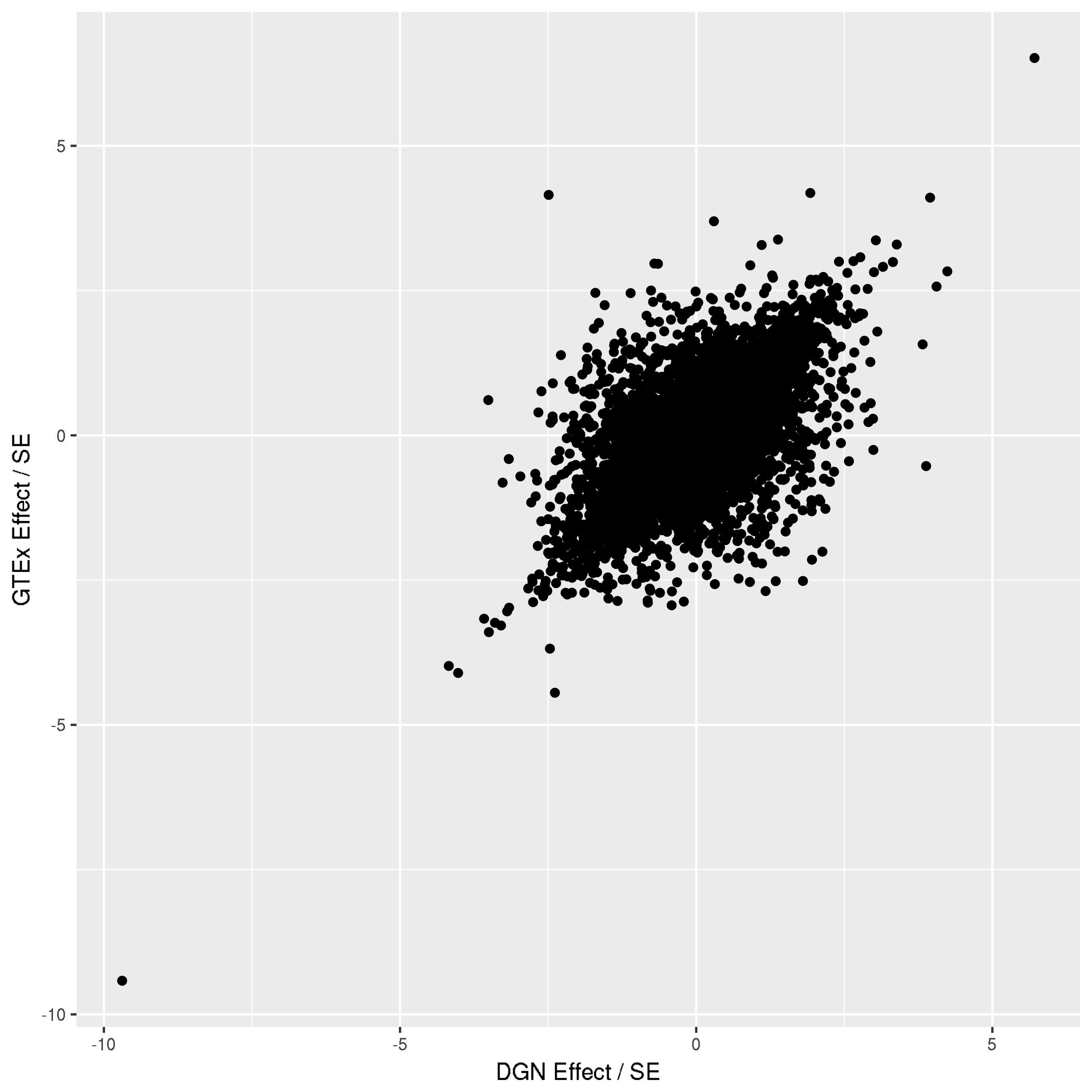


n) Triglycerides


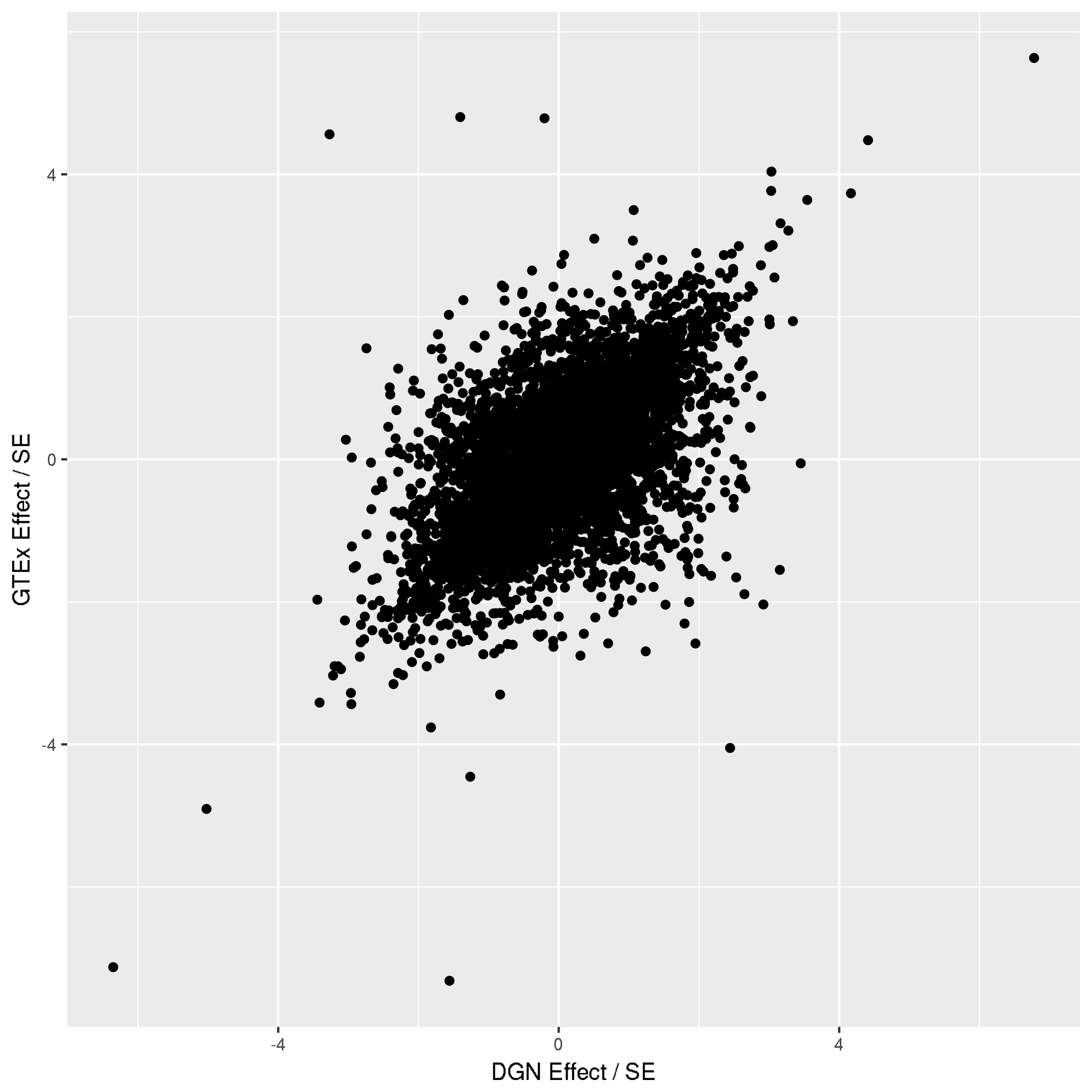


o) White blood cell count


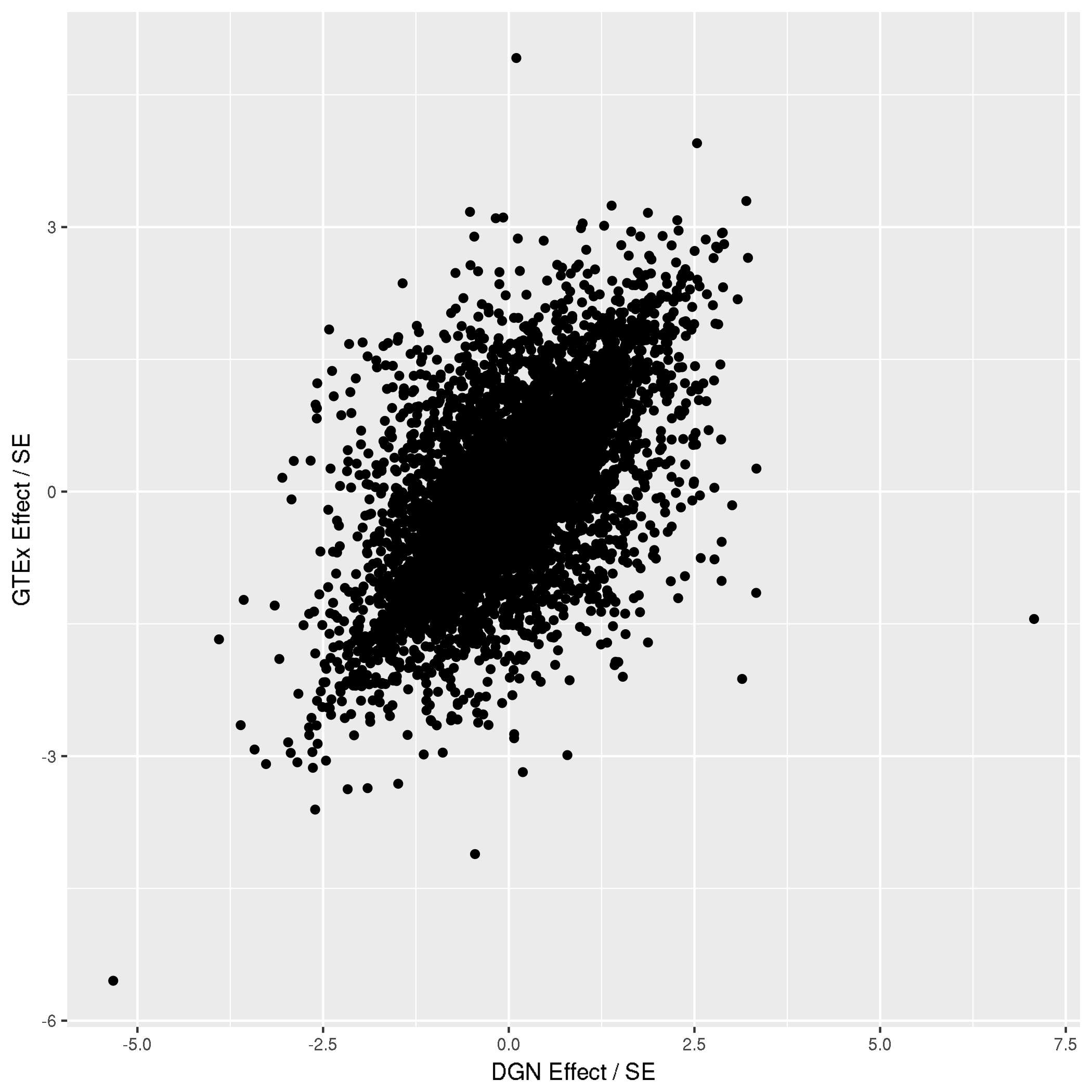


Supplementary Figure 3: Comparison of explained variance in African- and European-ancestry samples

Supplementary Figure 4: Comparison of variance explained (R^2^) and *p*-value of the correlation between imputed GReX and measured expression in YRI LCLs


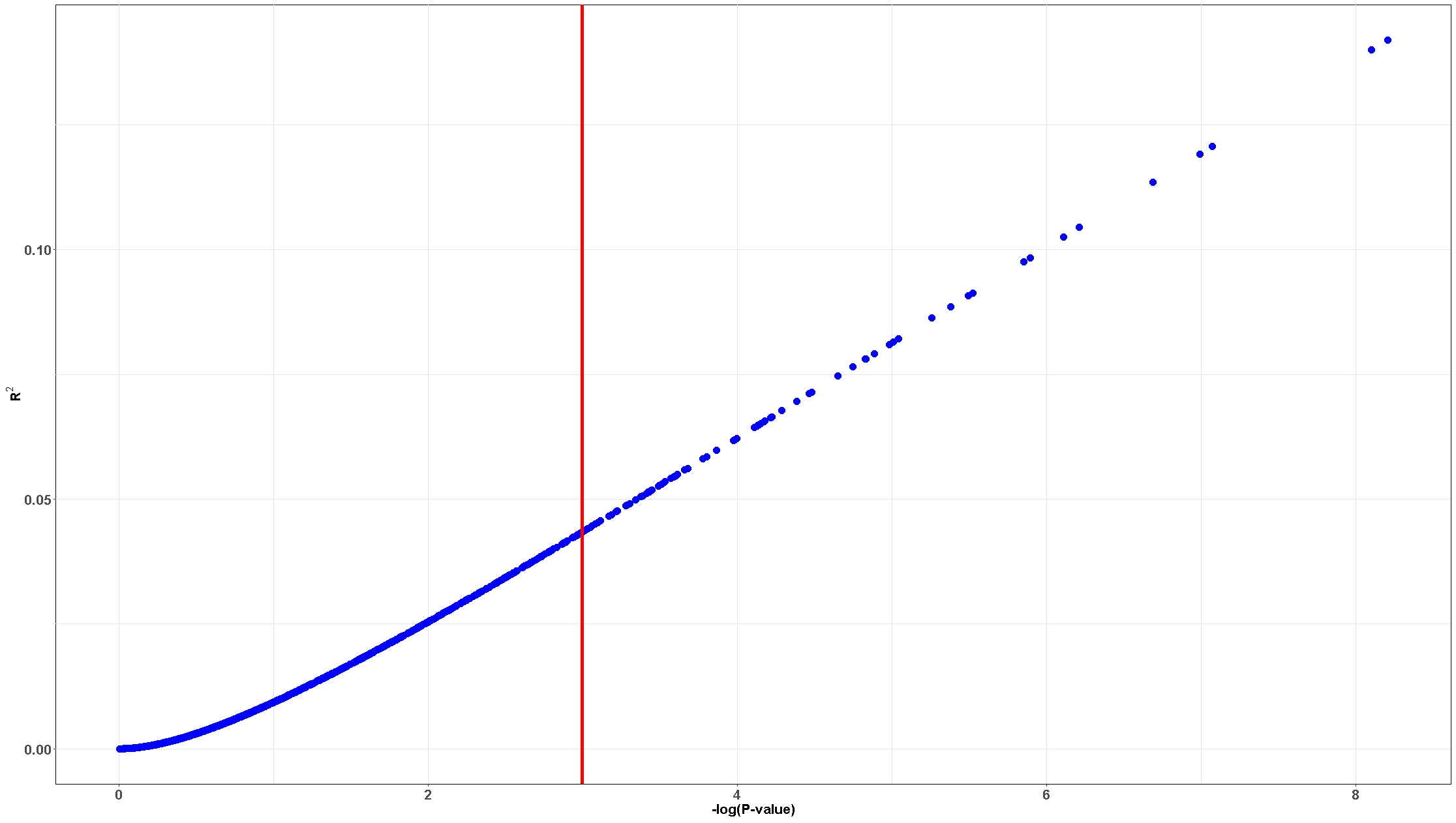


Supplementary Figure 5: Flow chart of methods and results classification


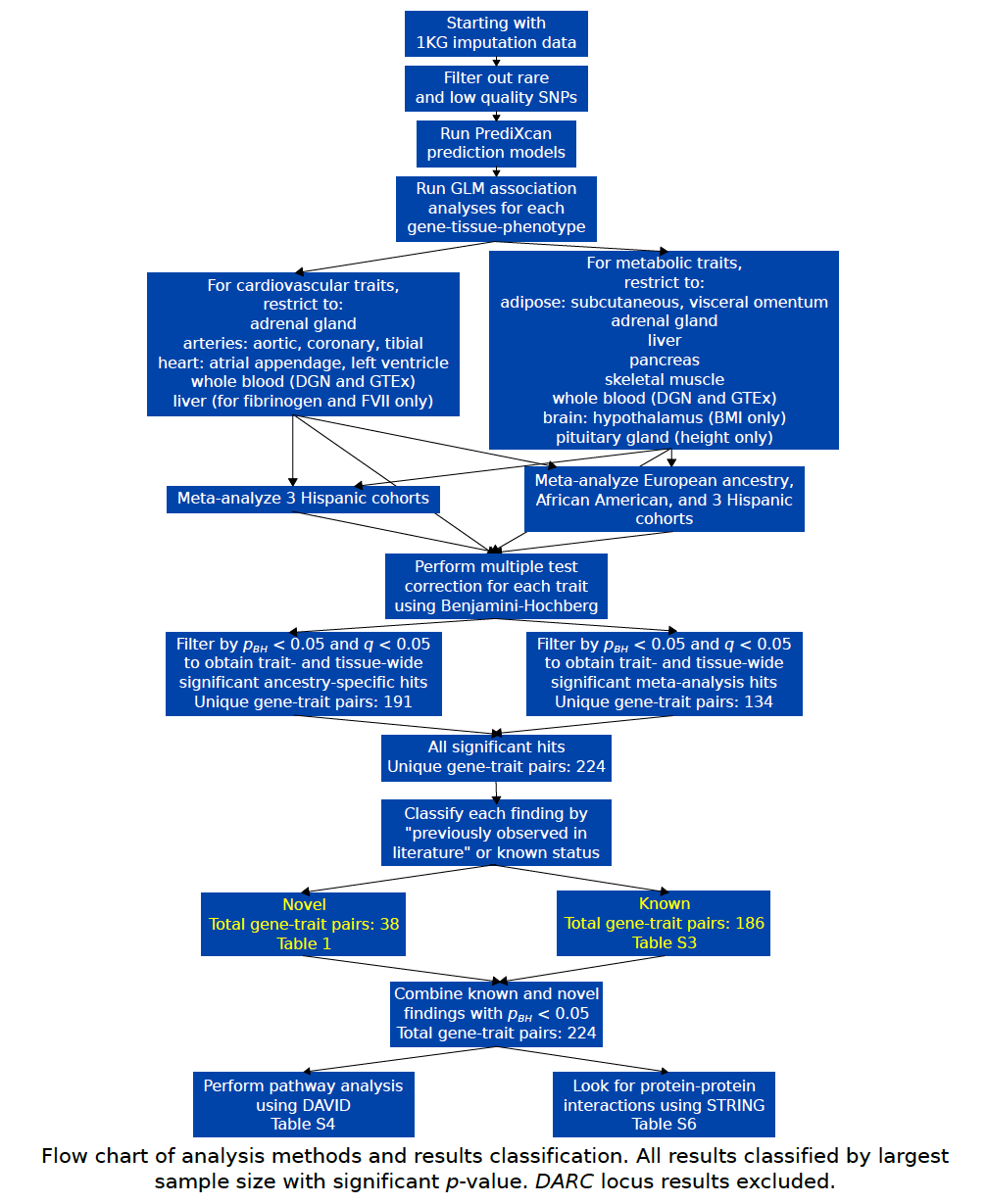


Supplementary Figure 6: Quantile-quantile plots for each trait-tissue combination separated by proximity to GWAS-associated variants. All genes are in black; genes within 250kb of a known variant are in blue, not previously associated genes are in red.

a) BMI


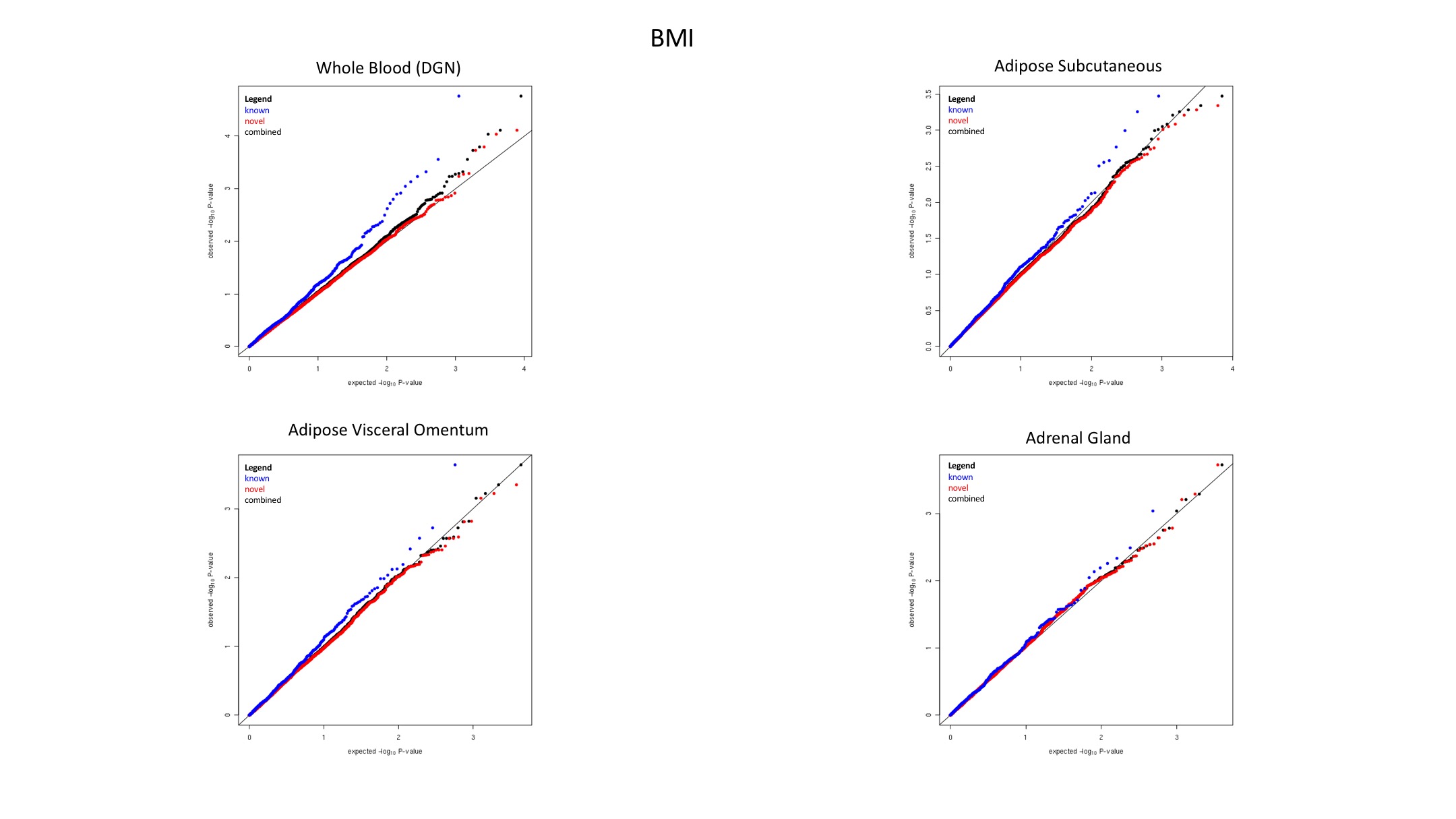


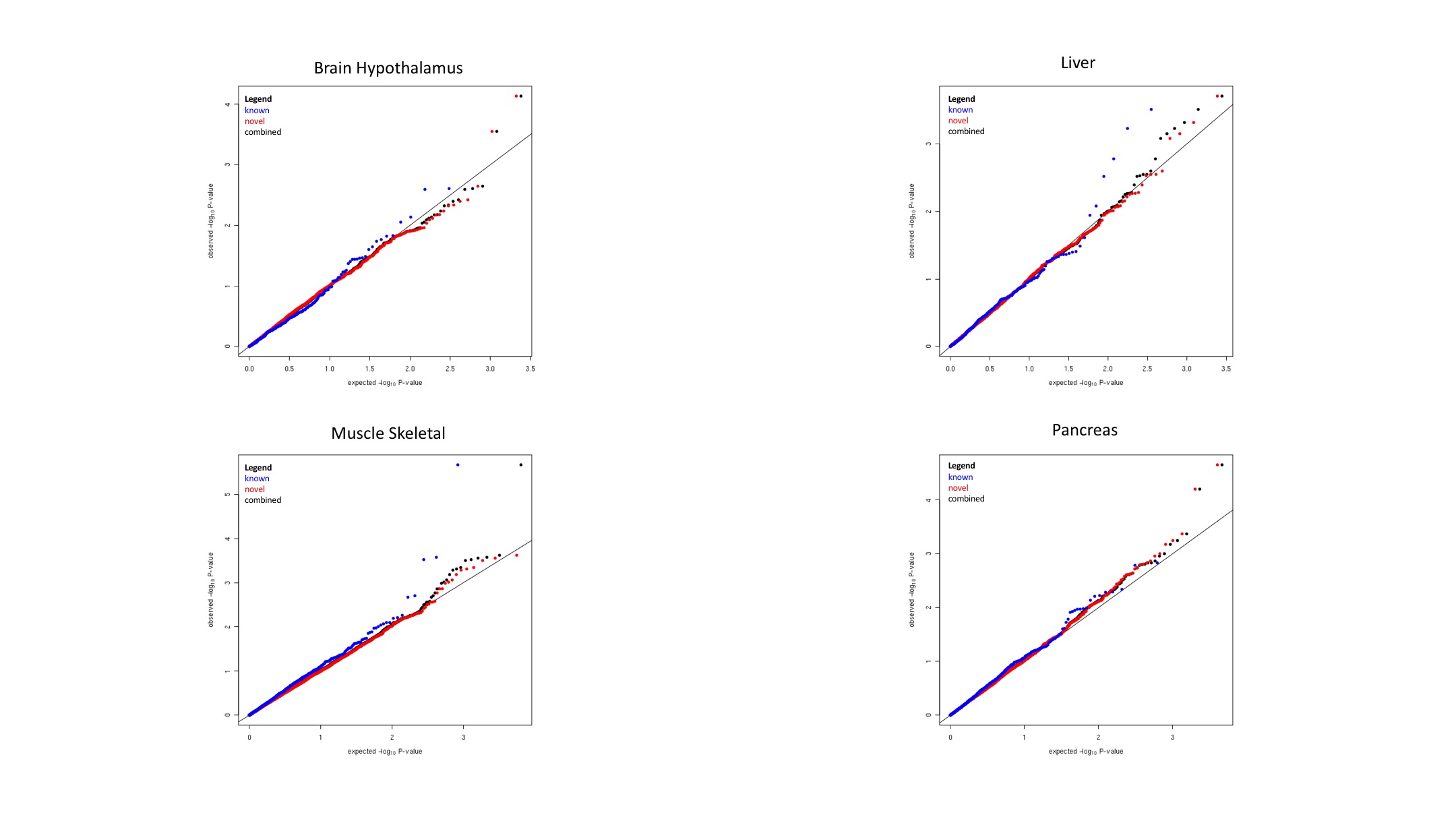

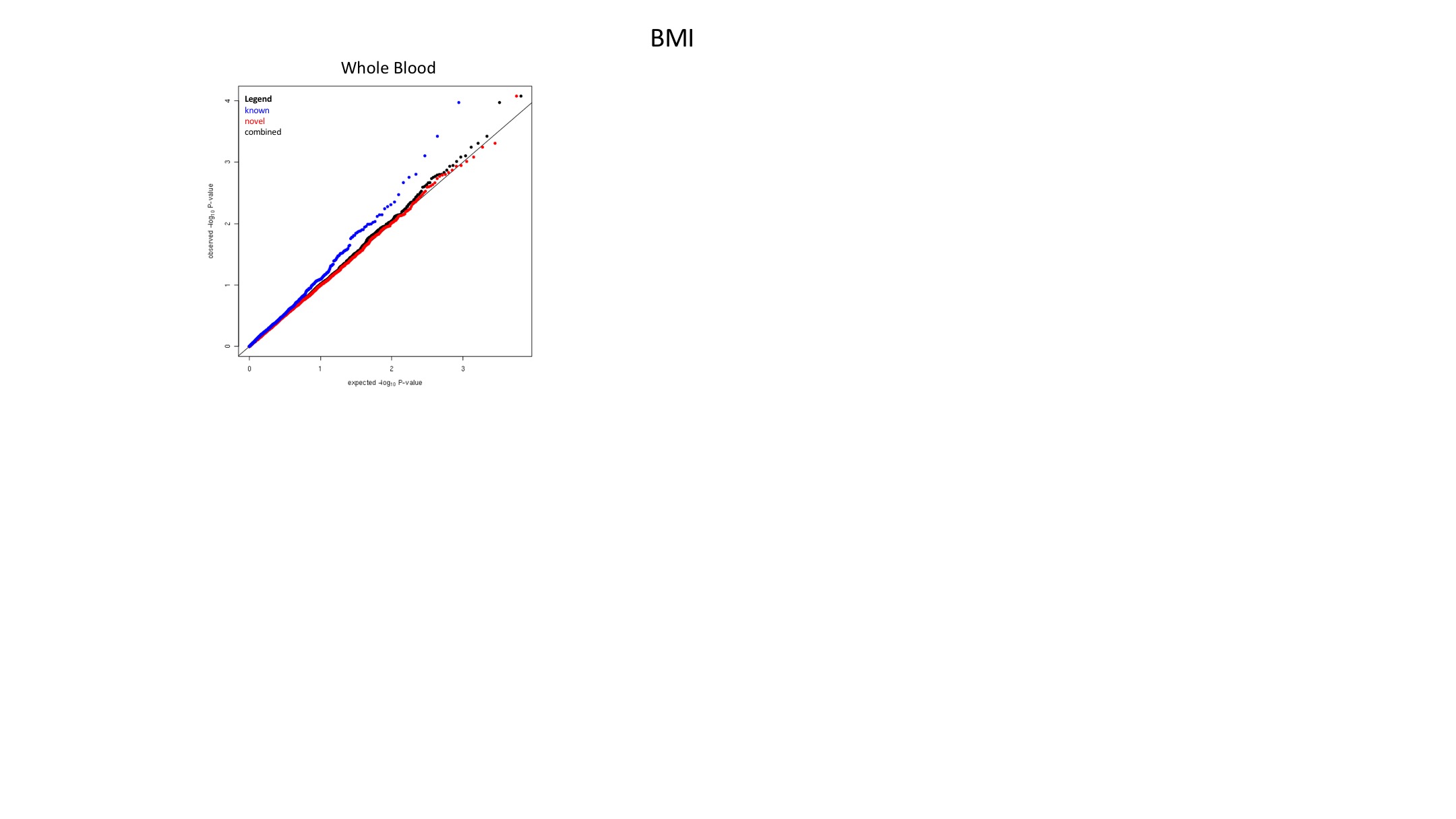


b) Cholesterol


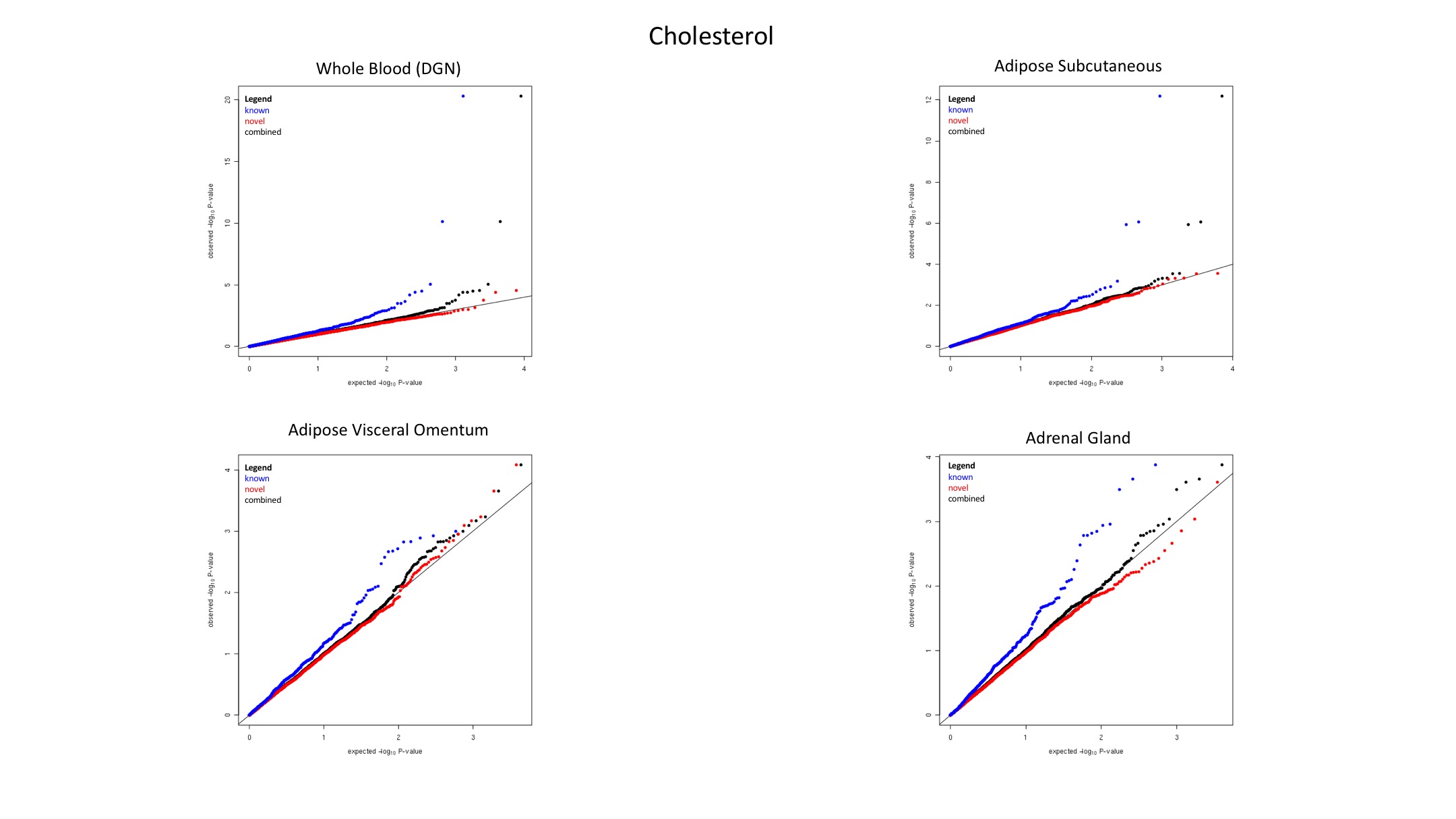


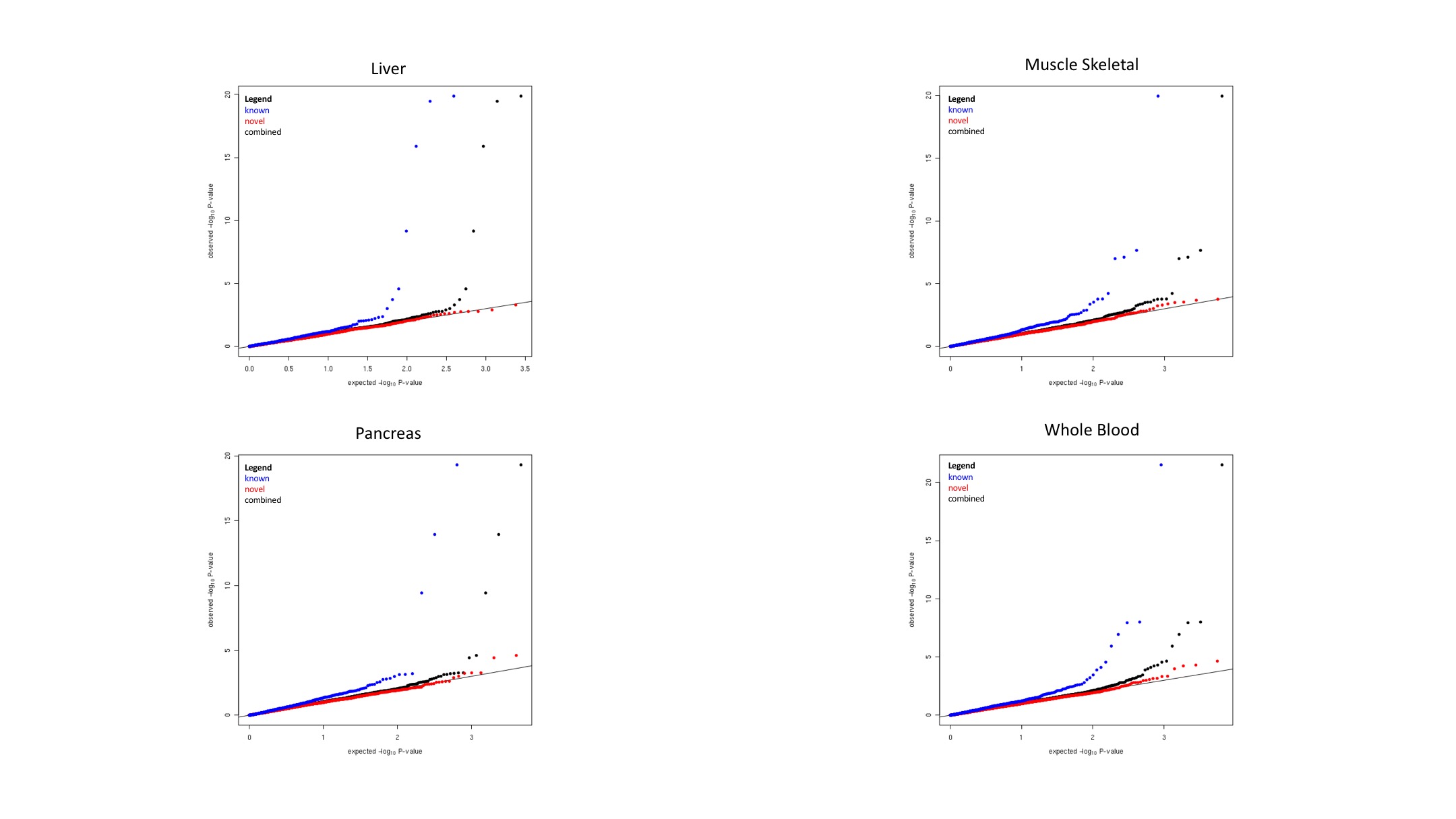


c) Diastolic Blood Pressure


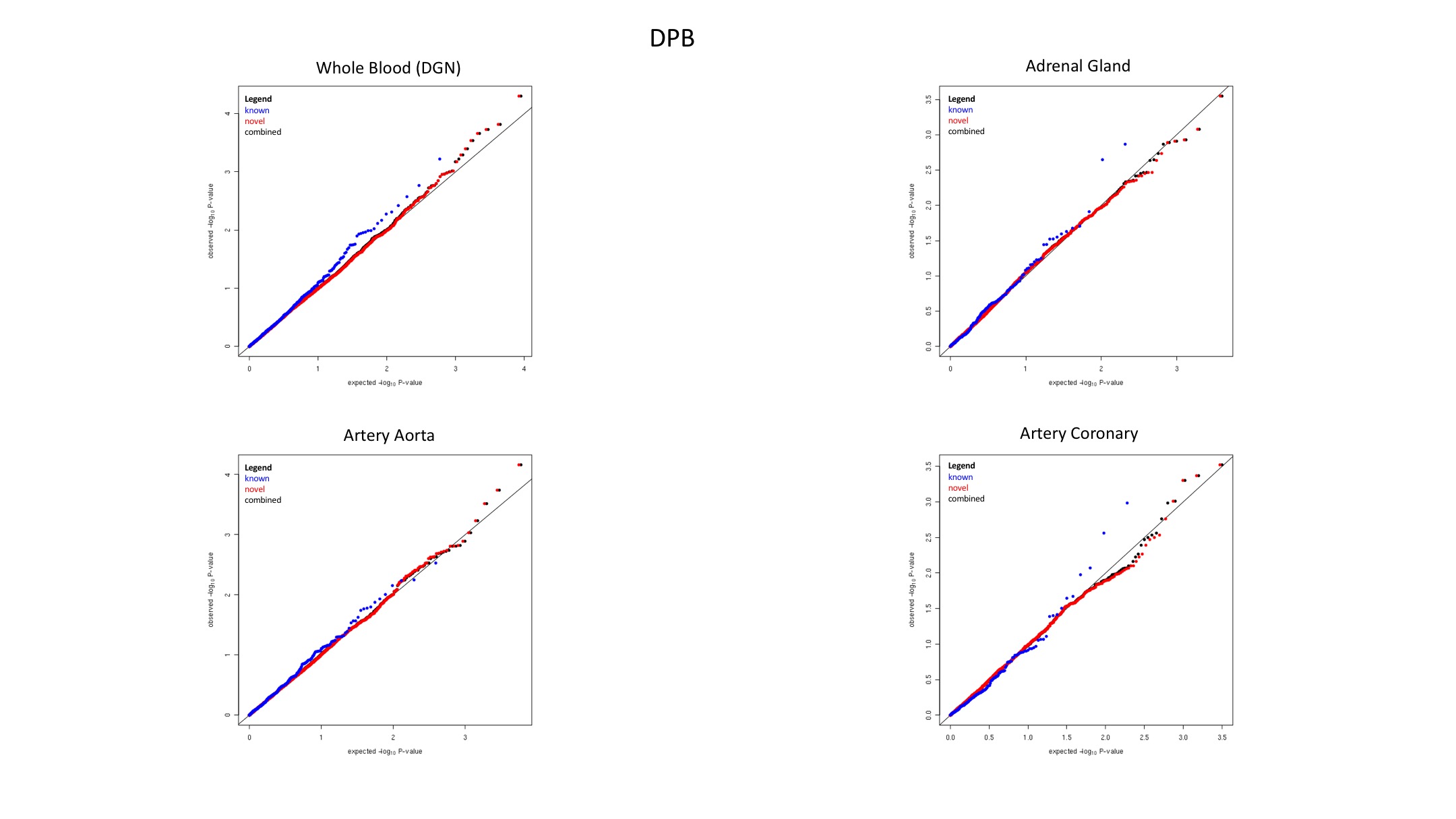


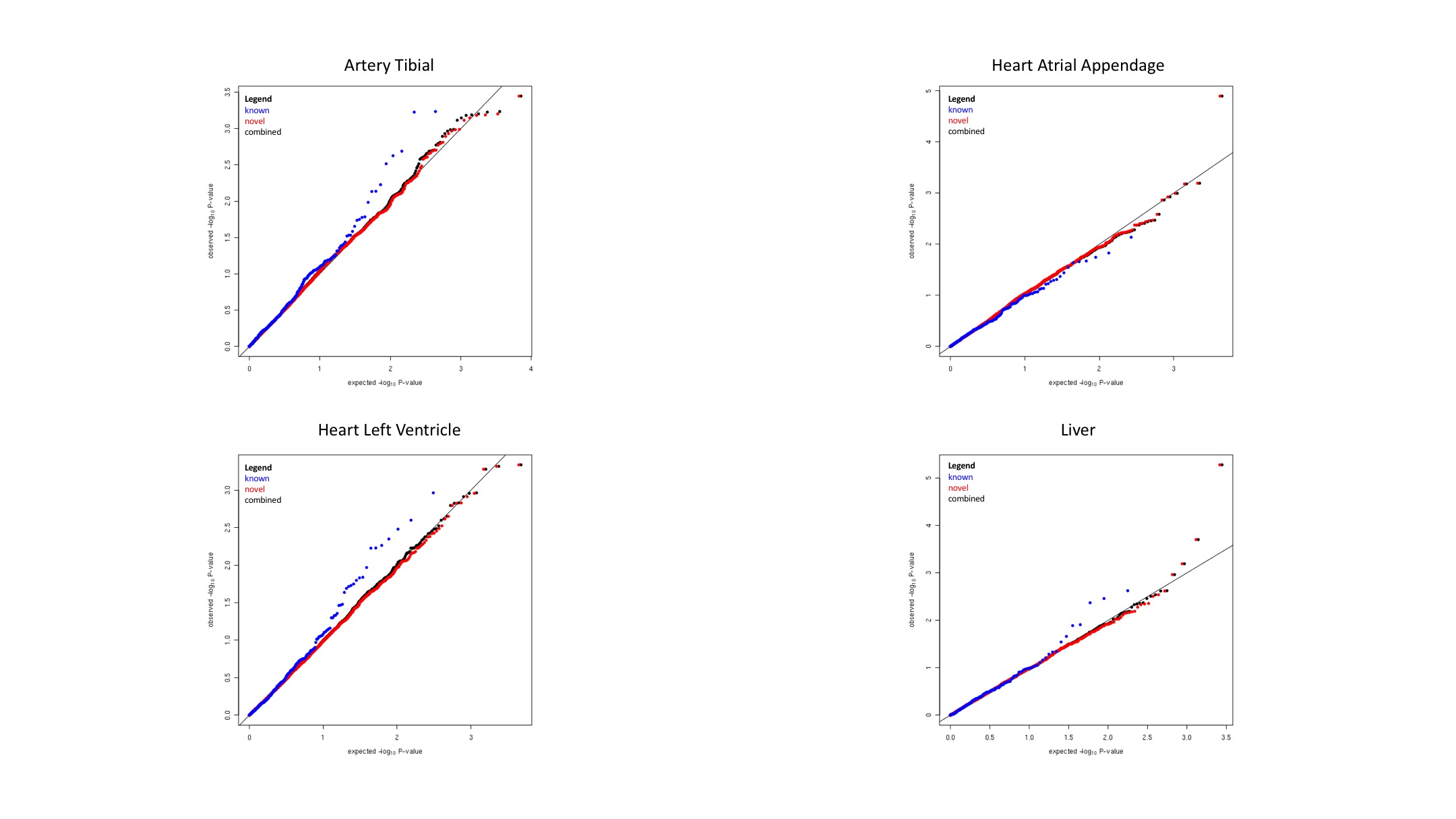


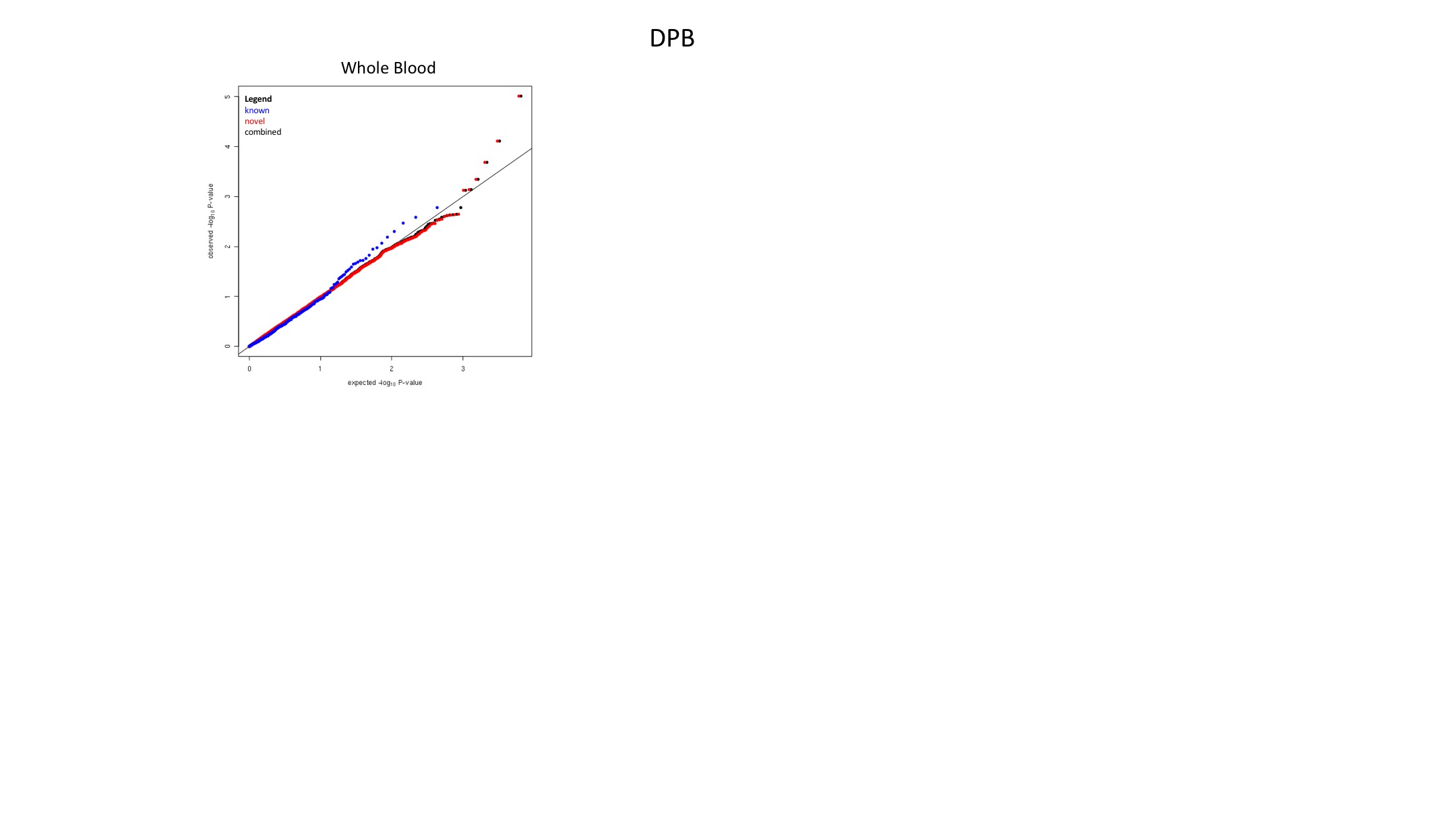


d) Fibrinogen


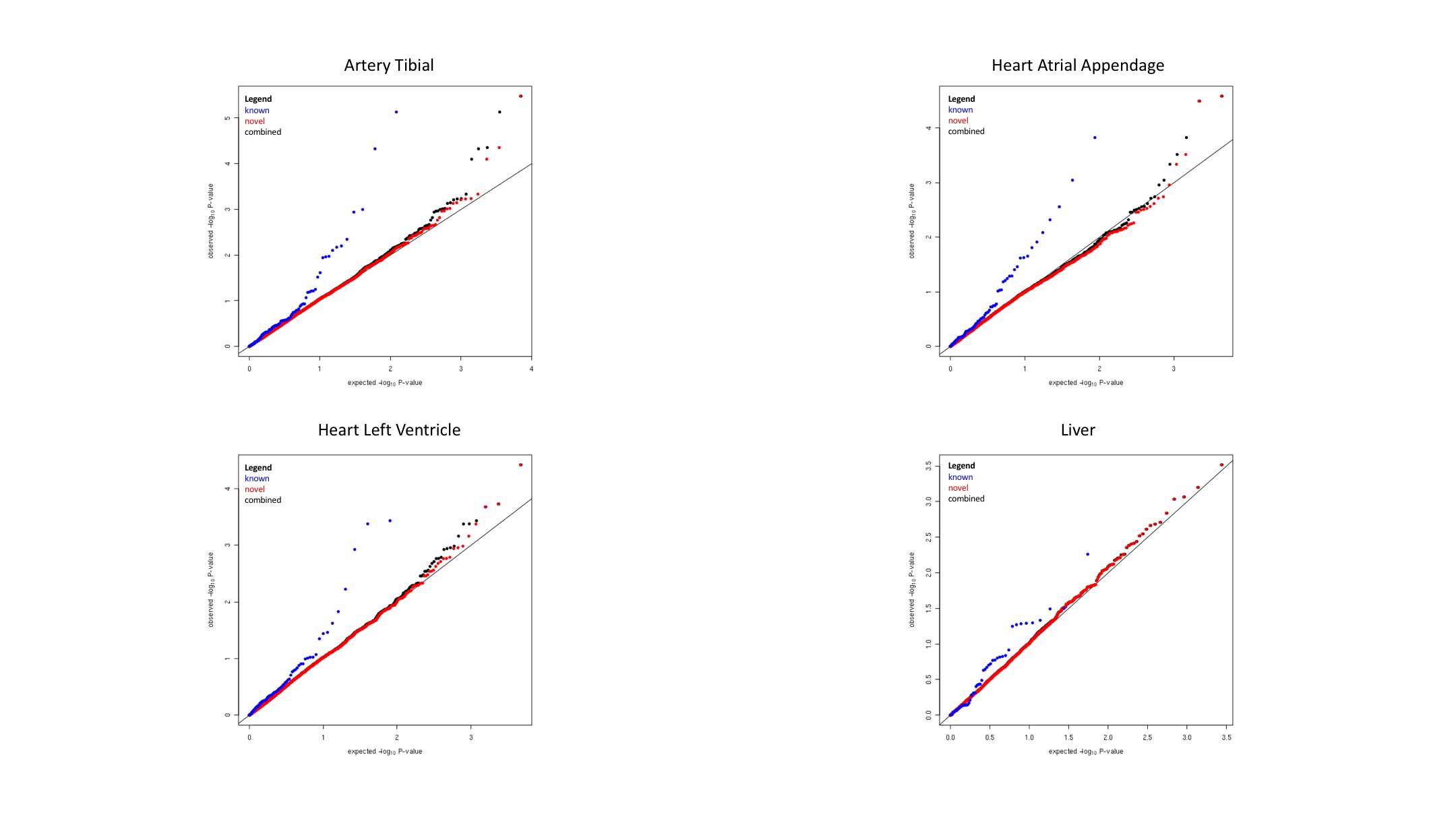

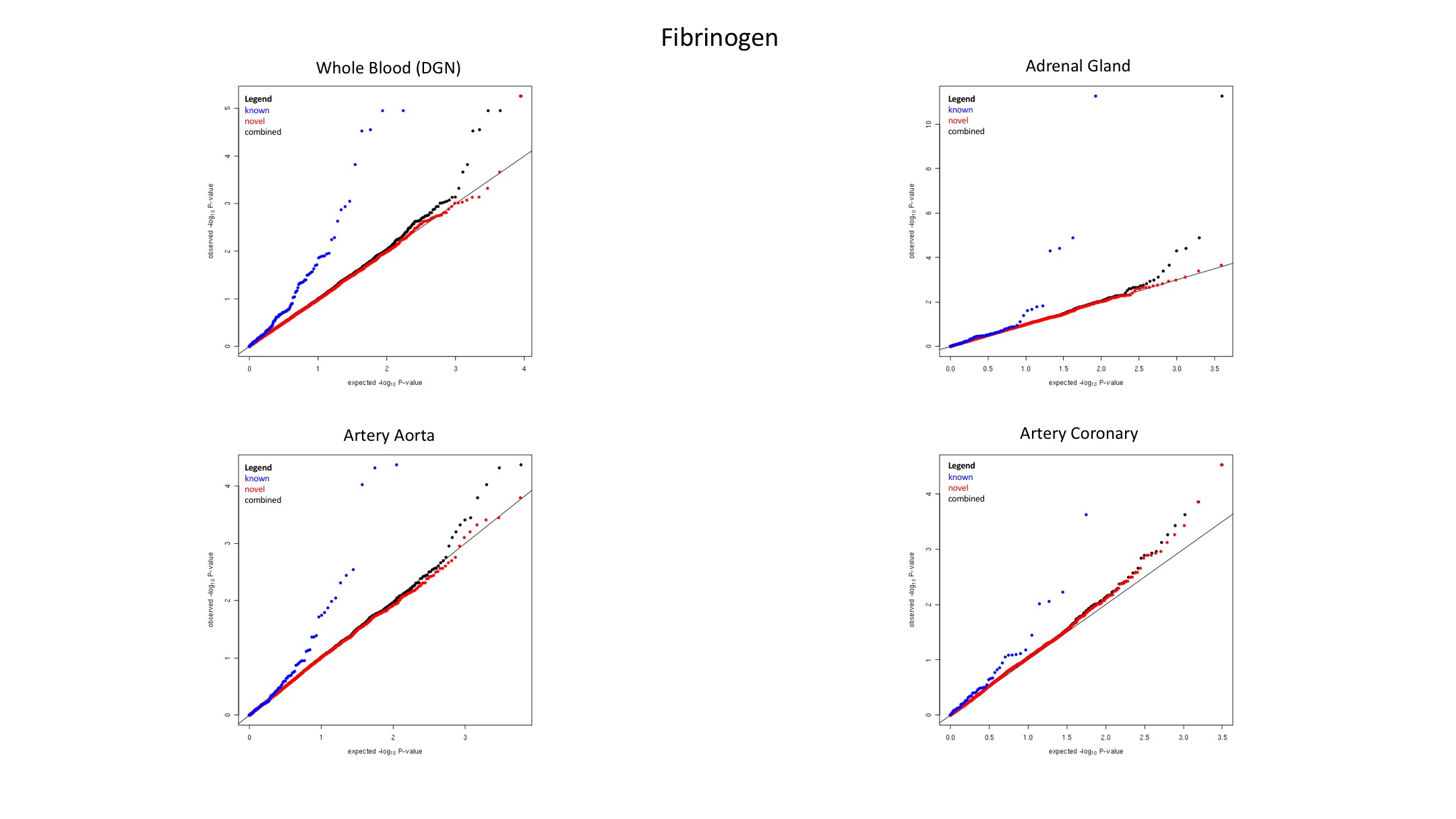


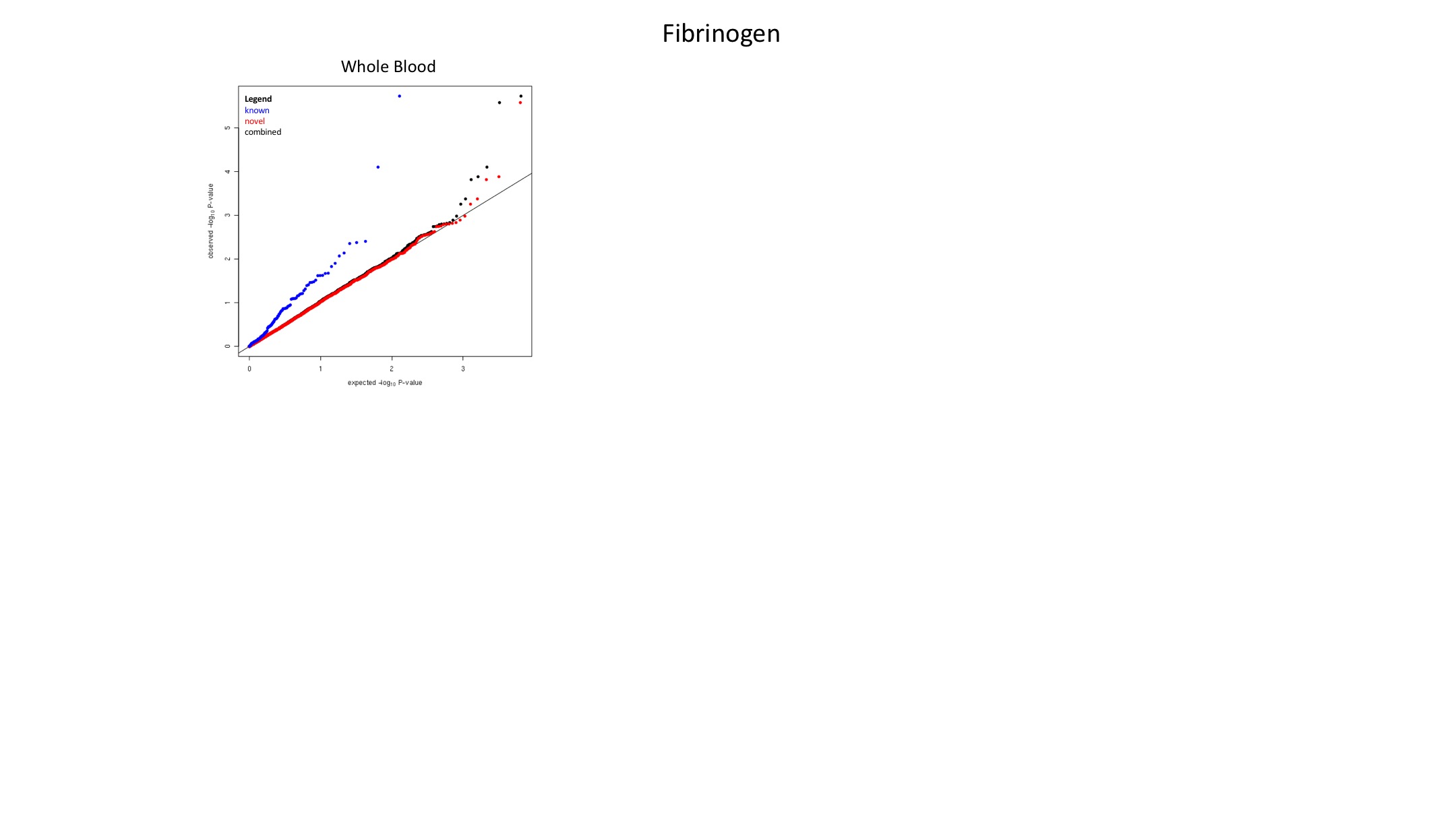


e) Factor VII


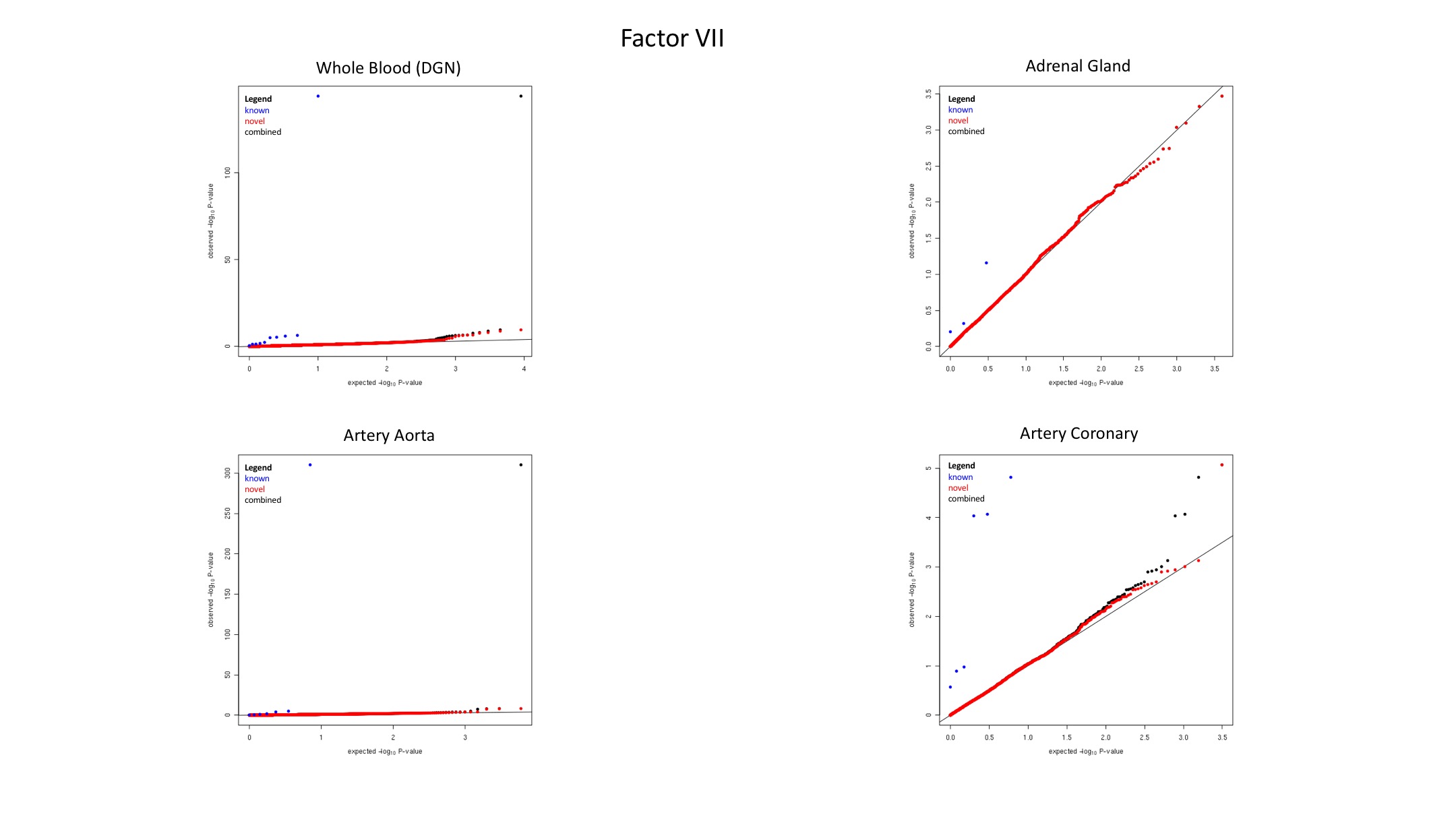

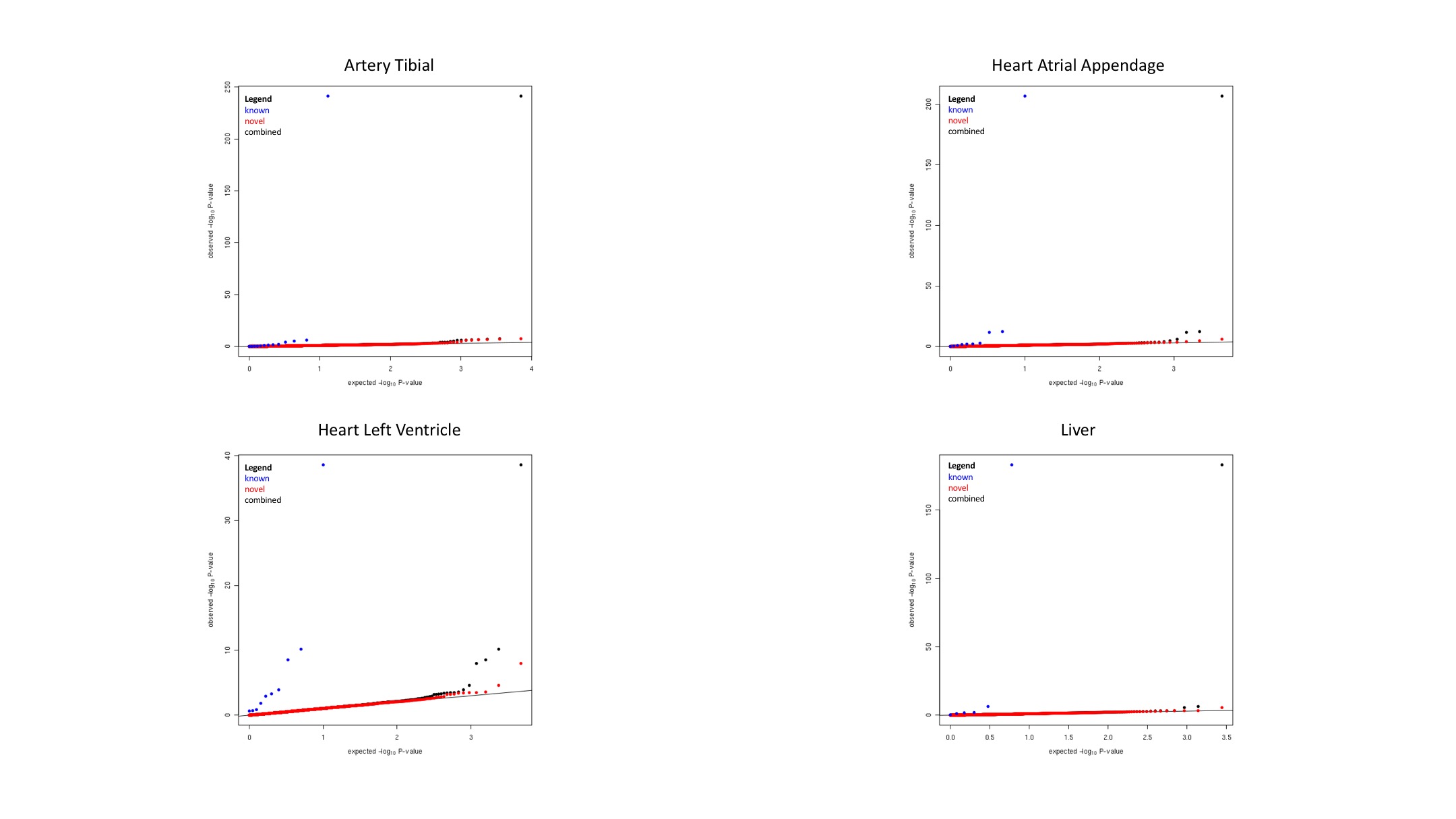


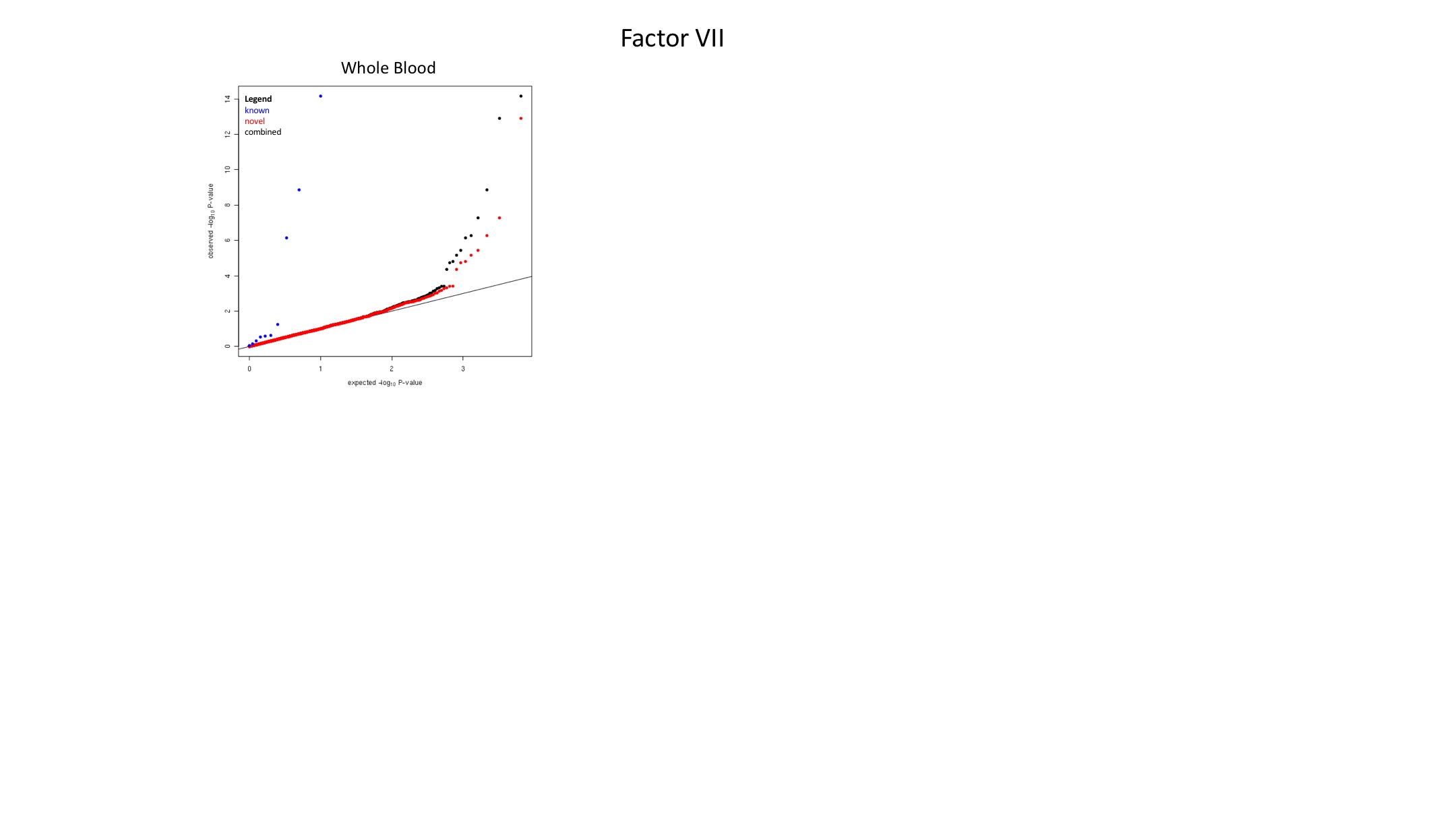


f) Glucose


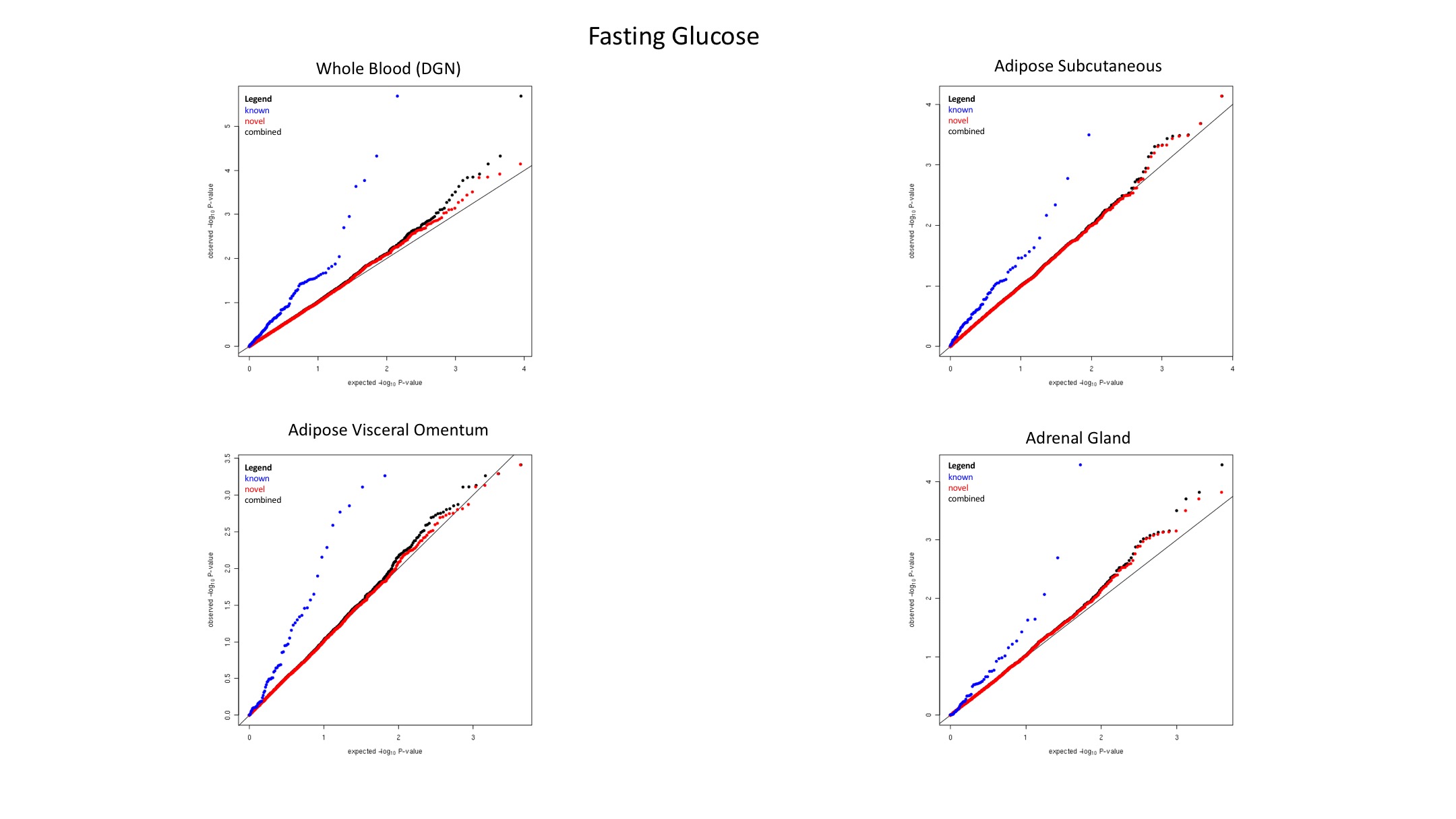


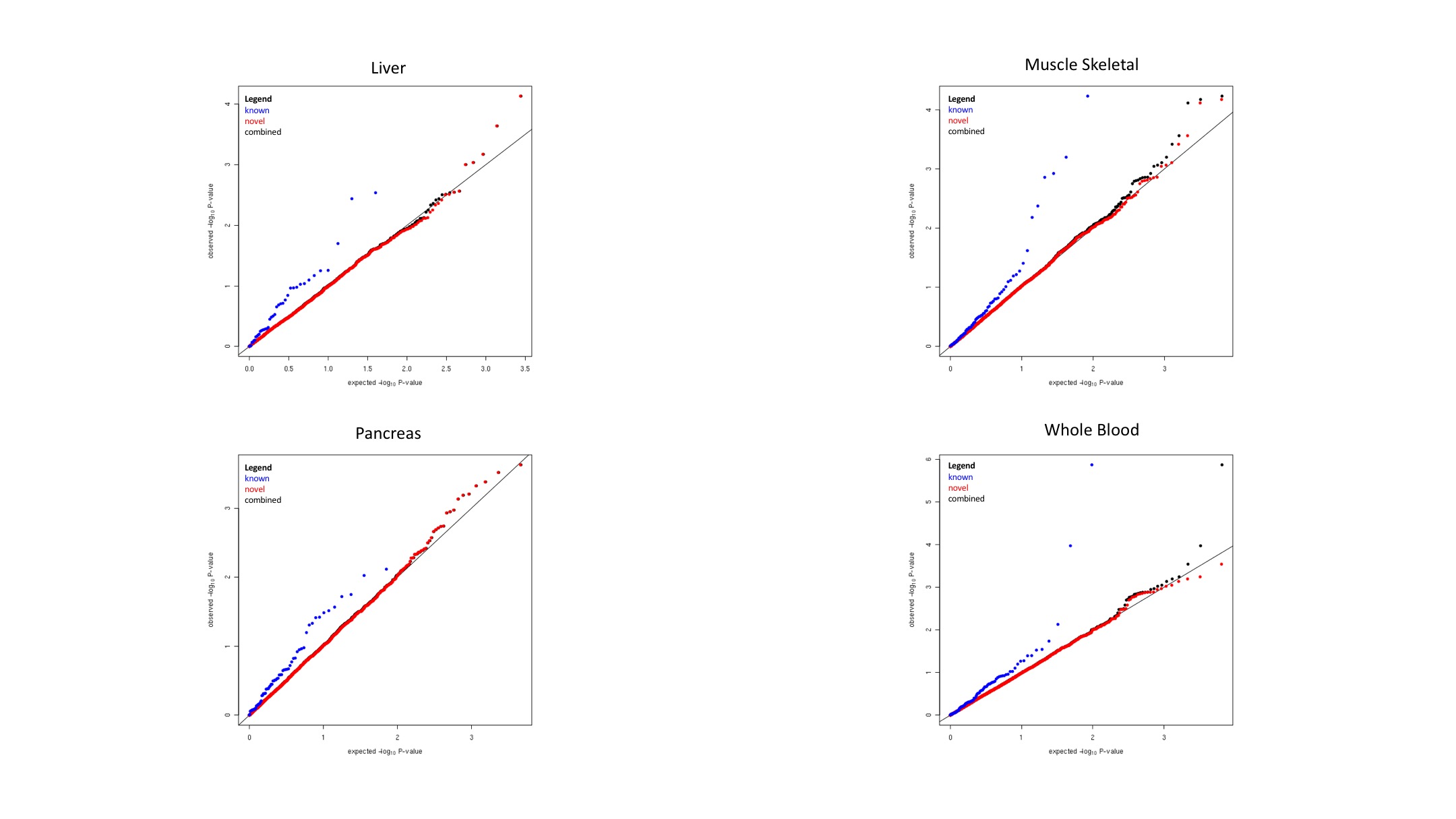


g) HDL


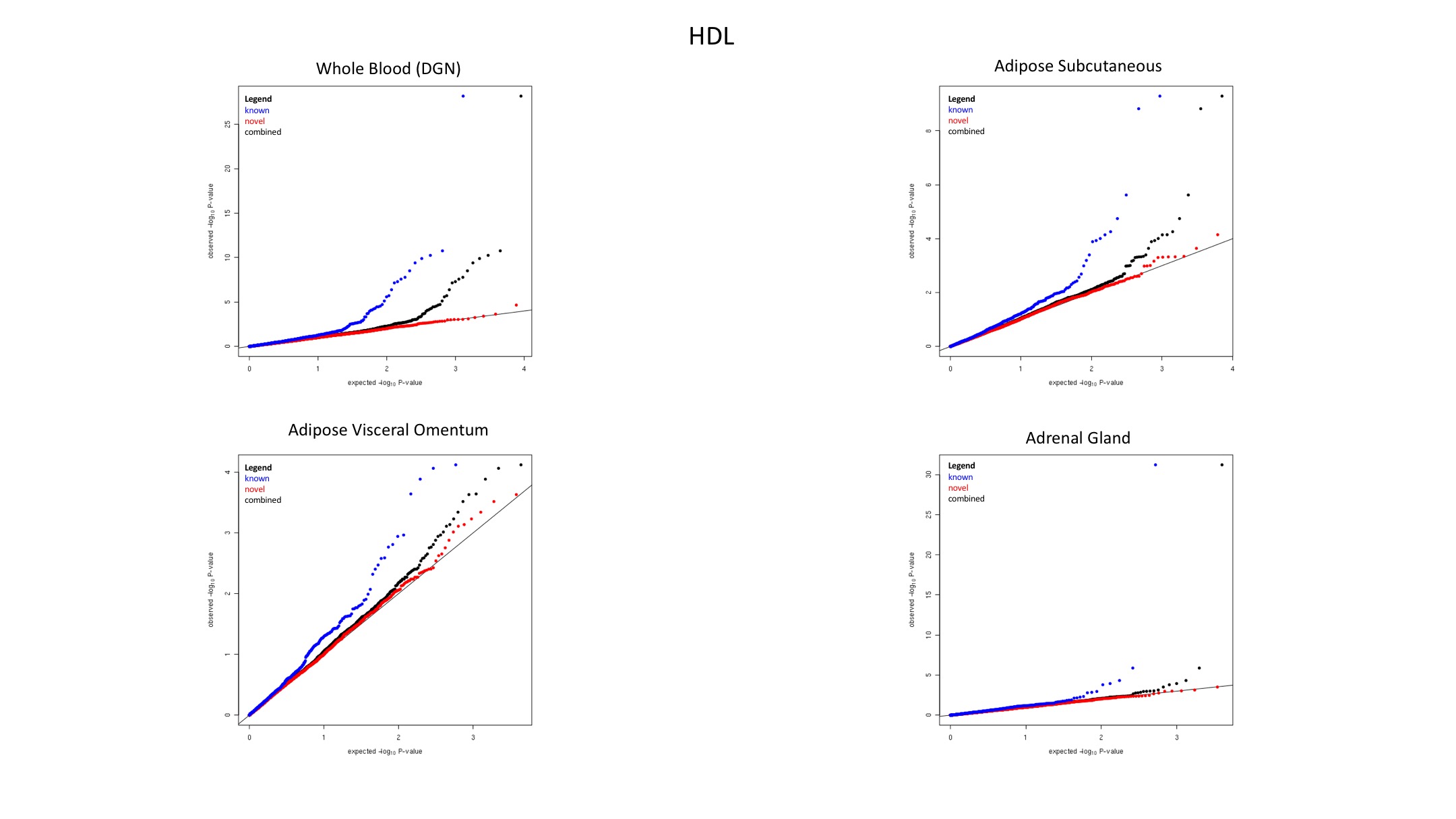

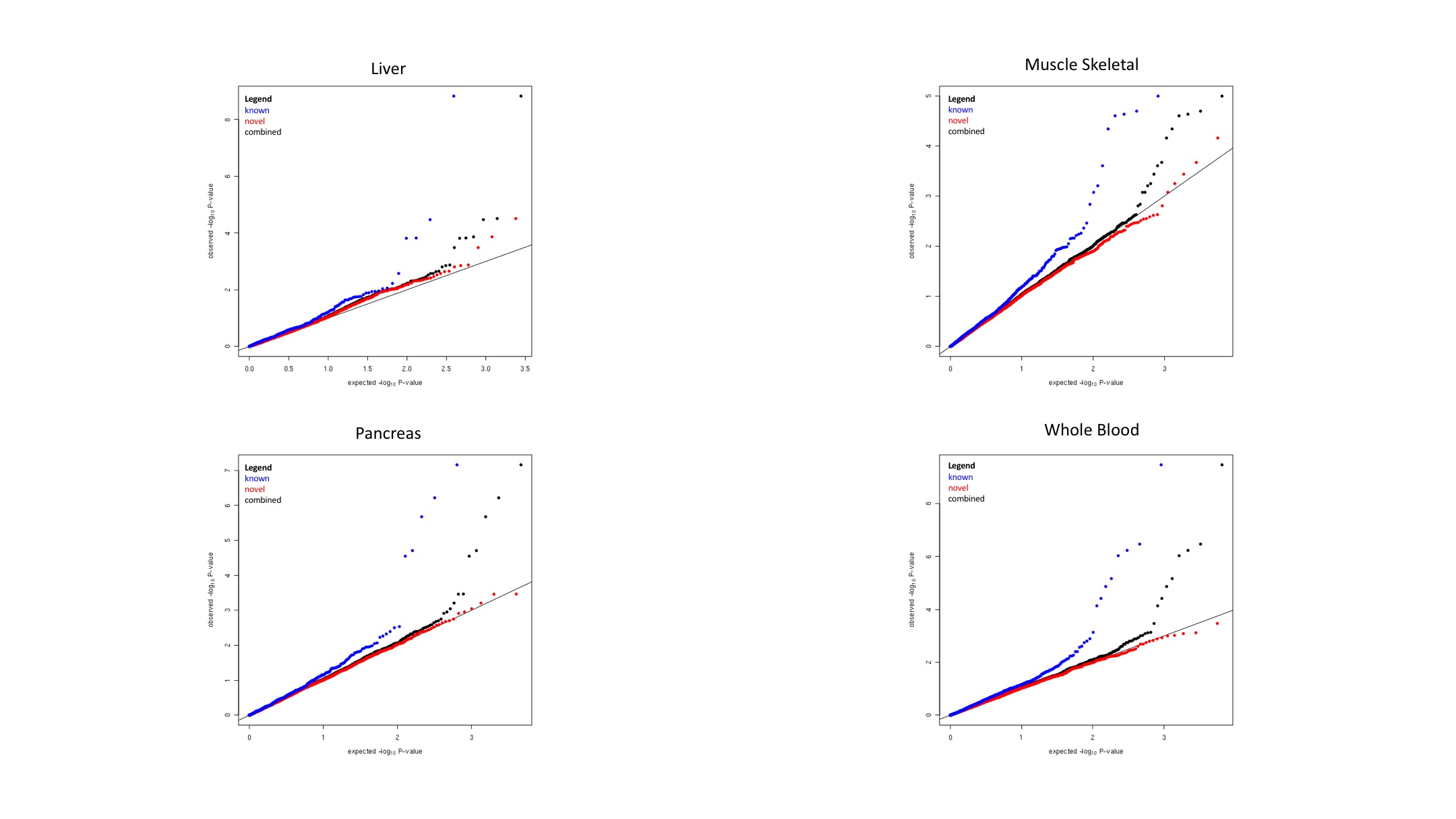


h) Height


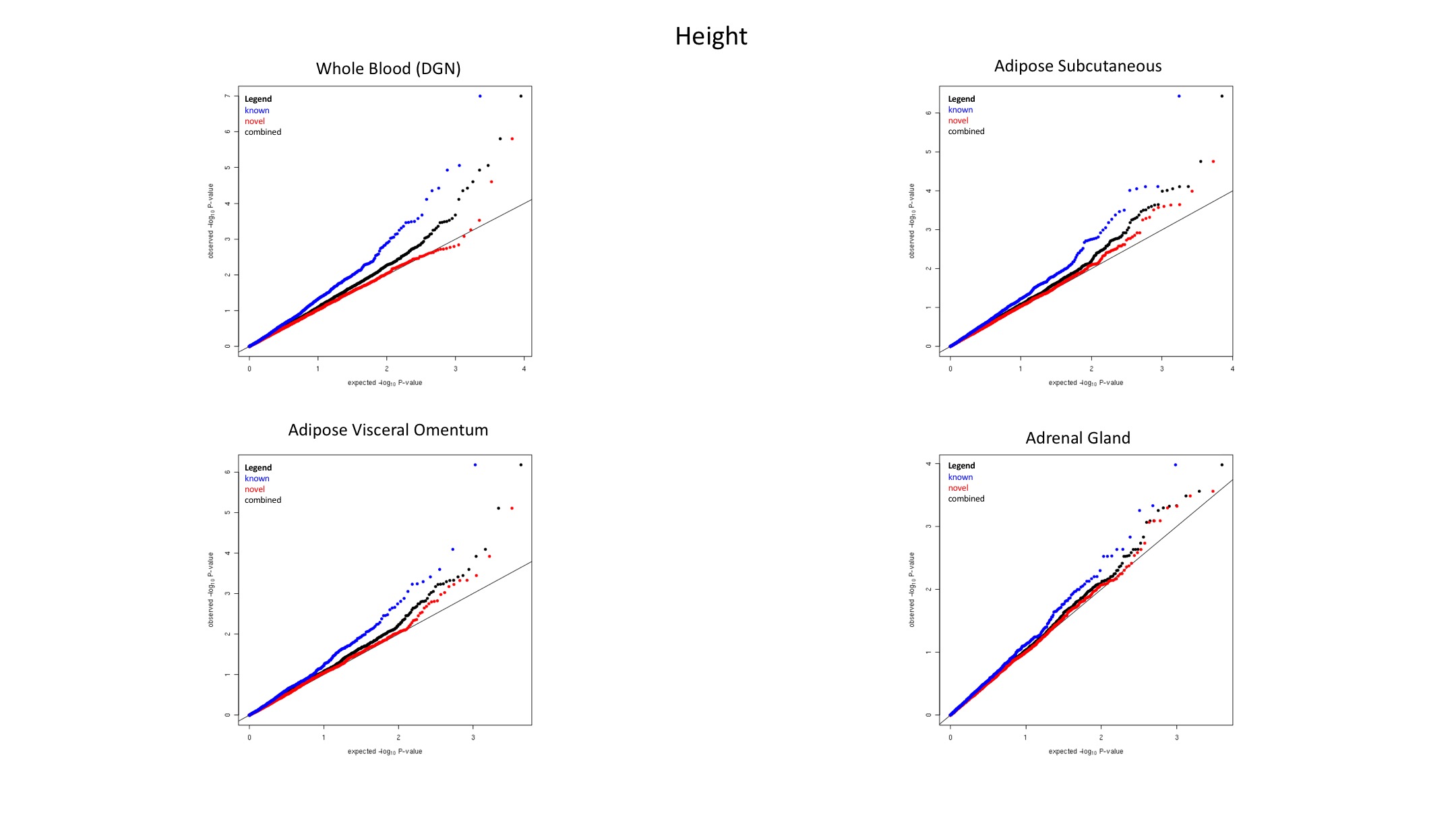


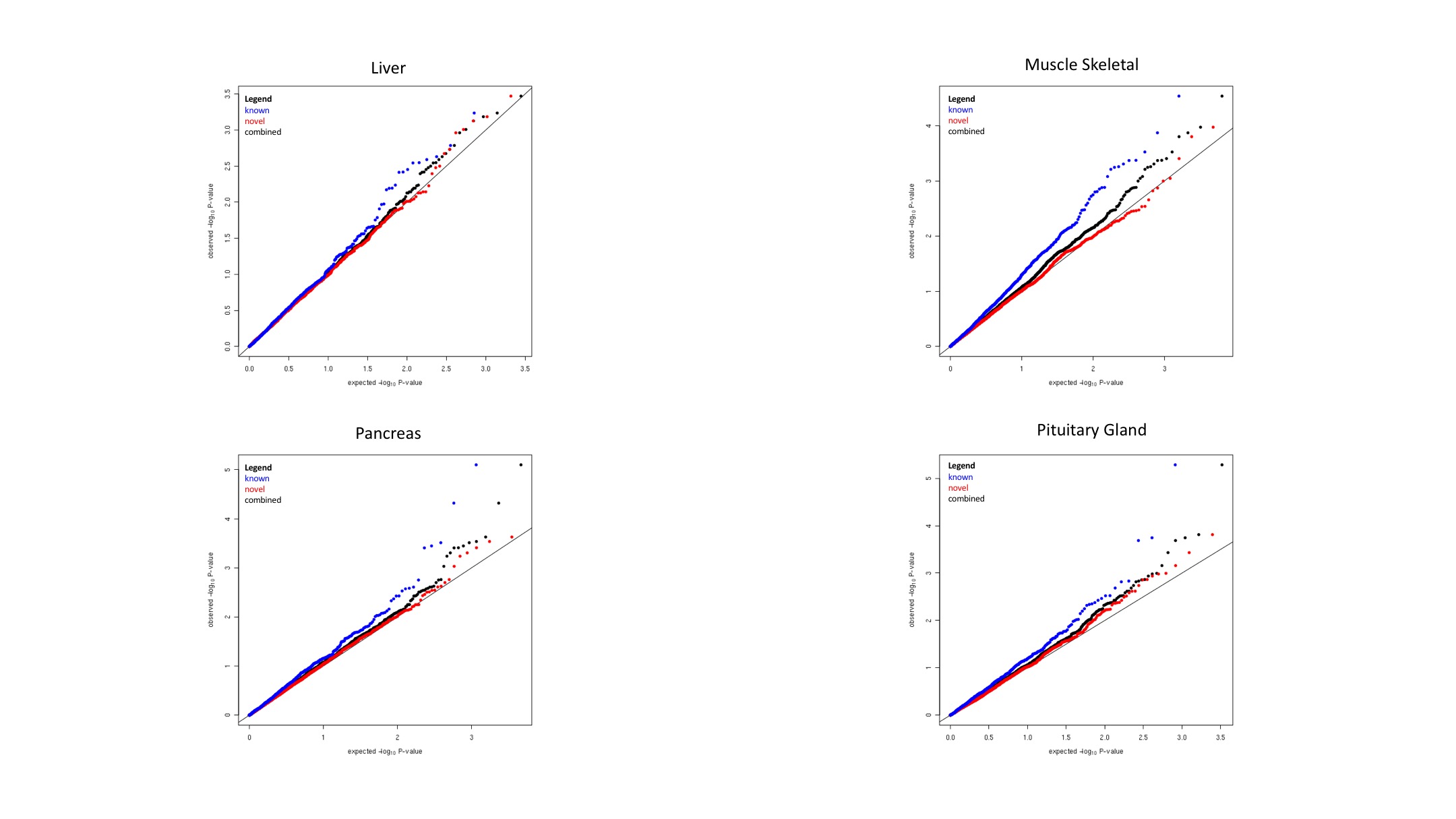


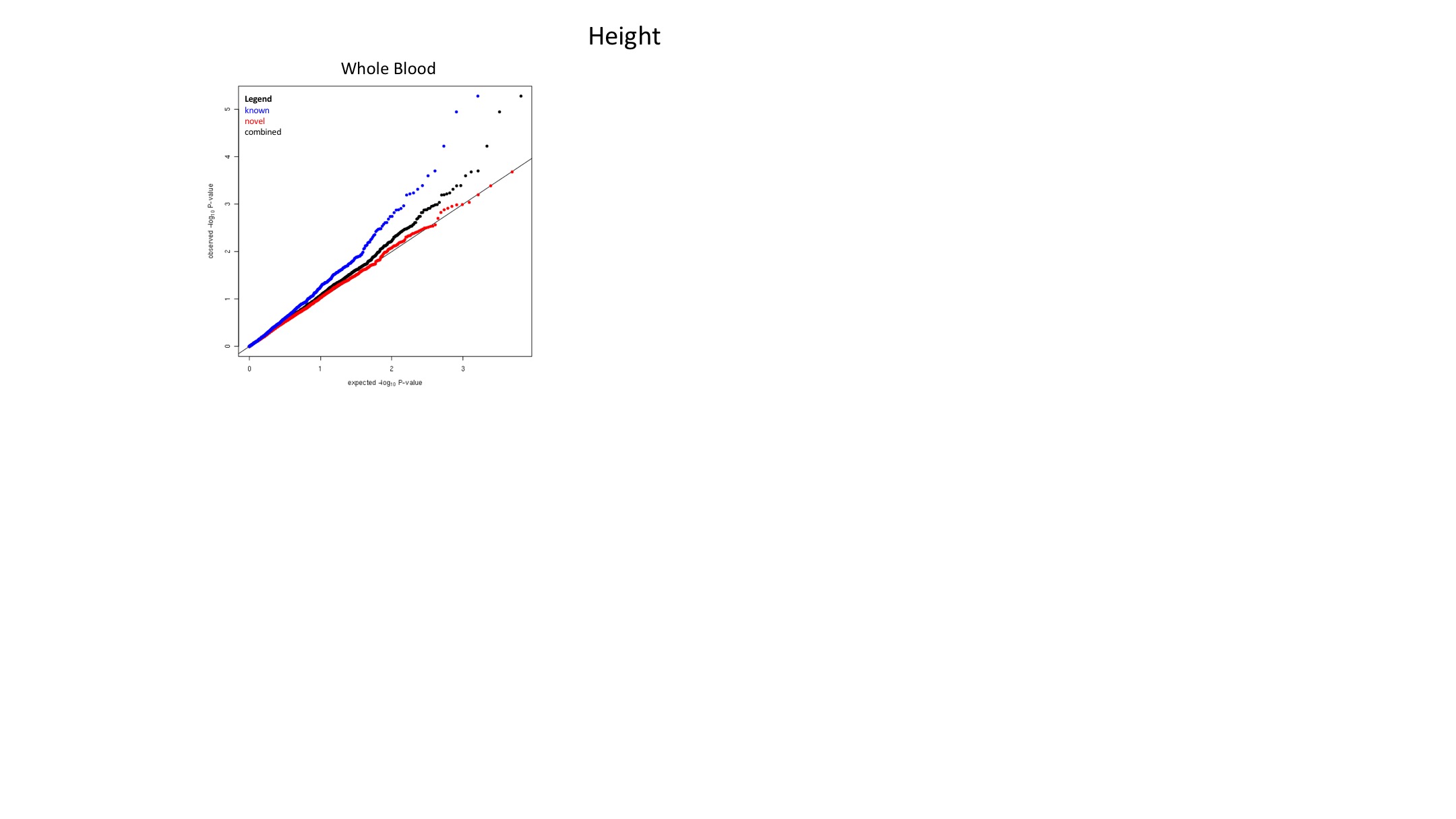


i) Insulin


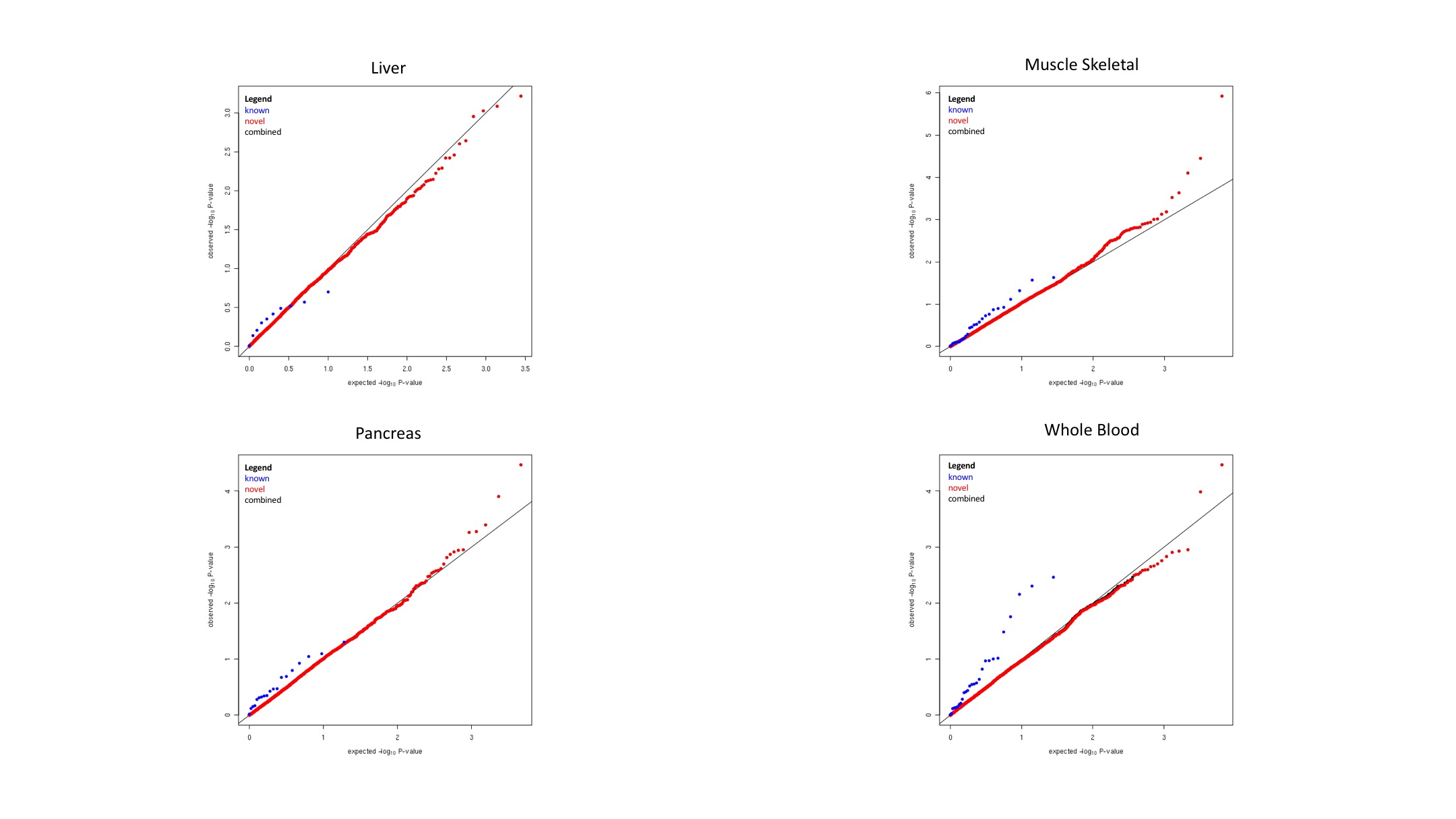

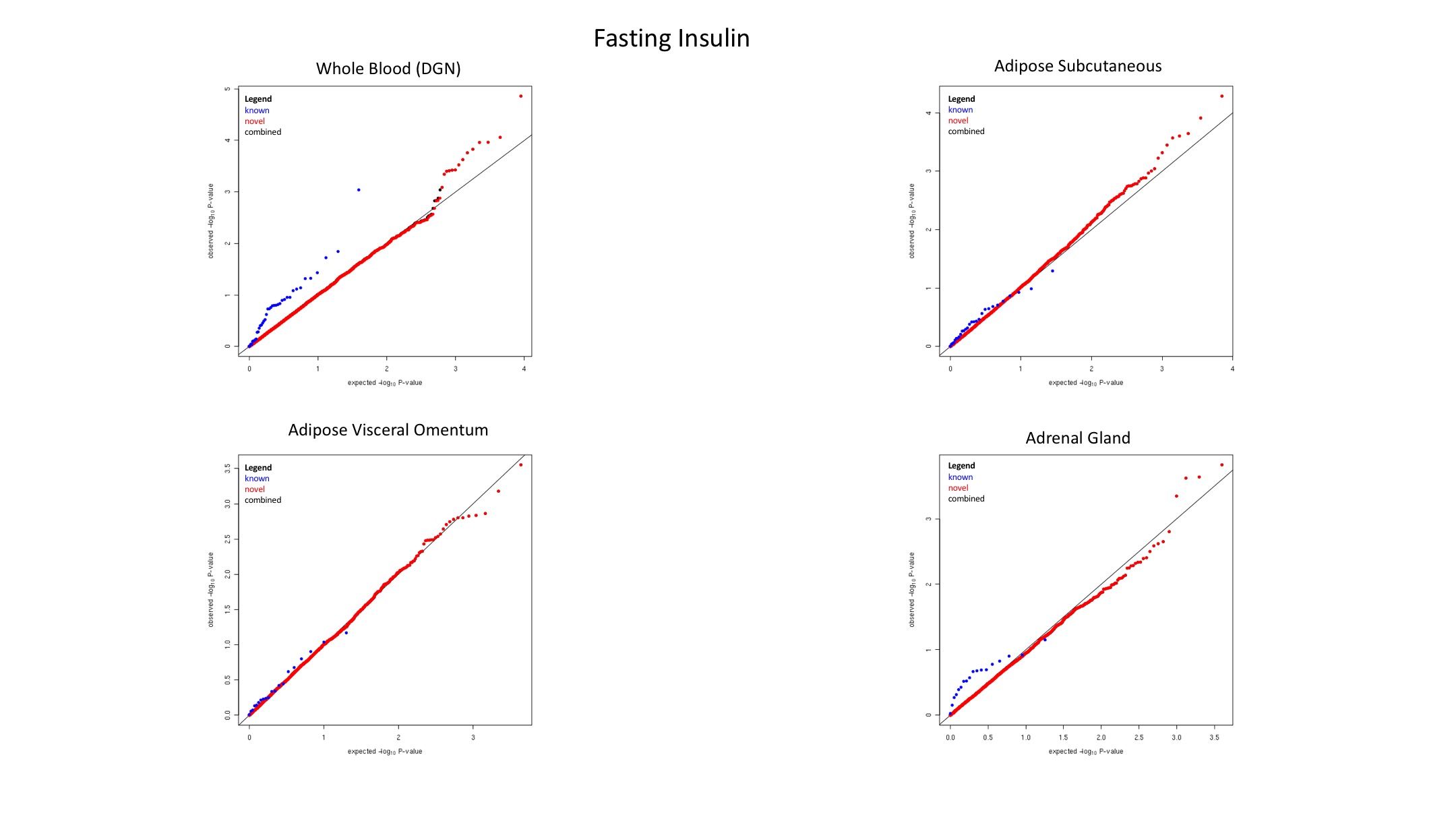


j) LDL


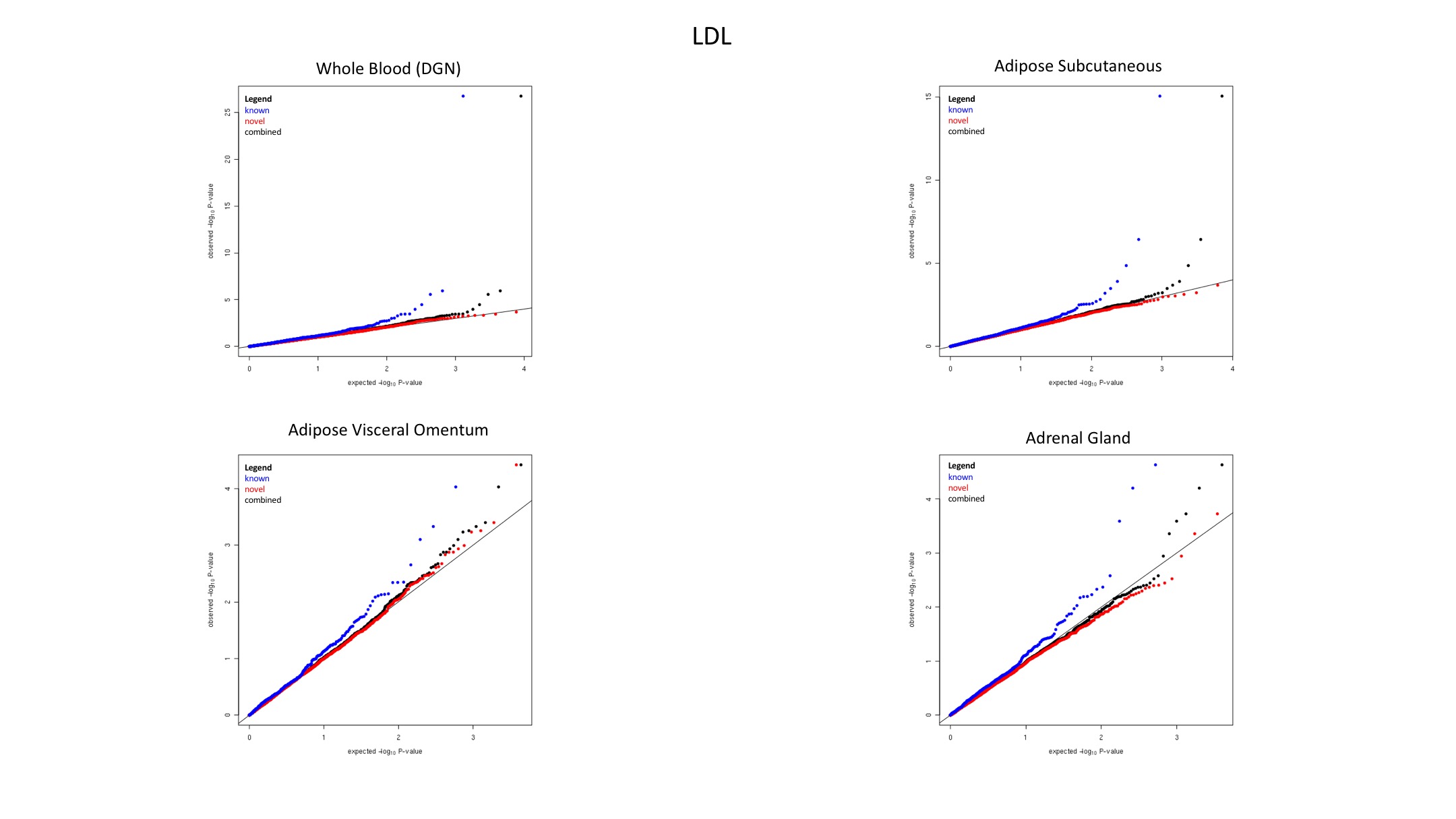

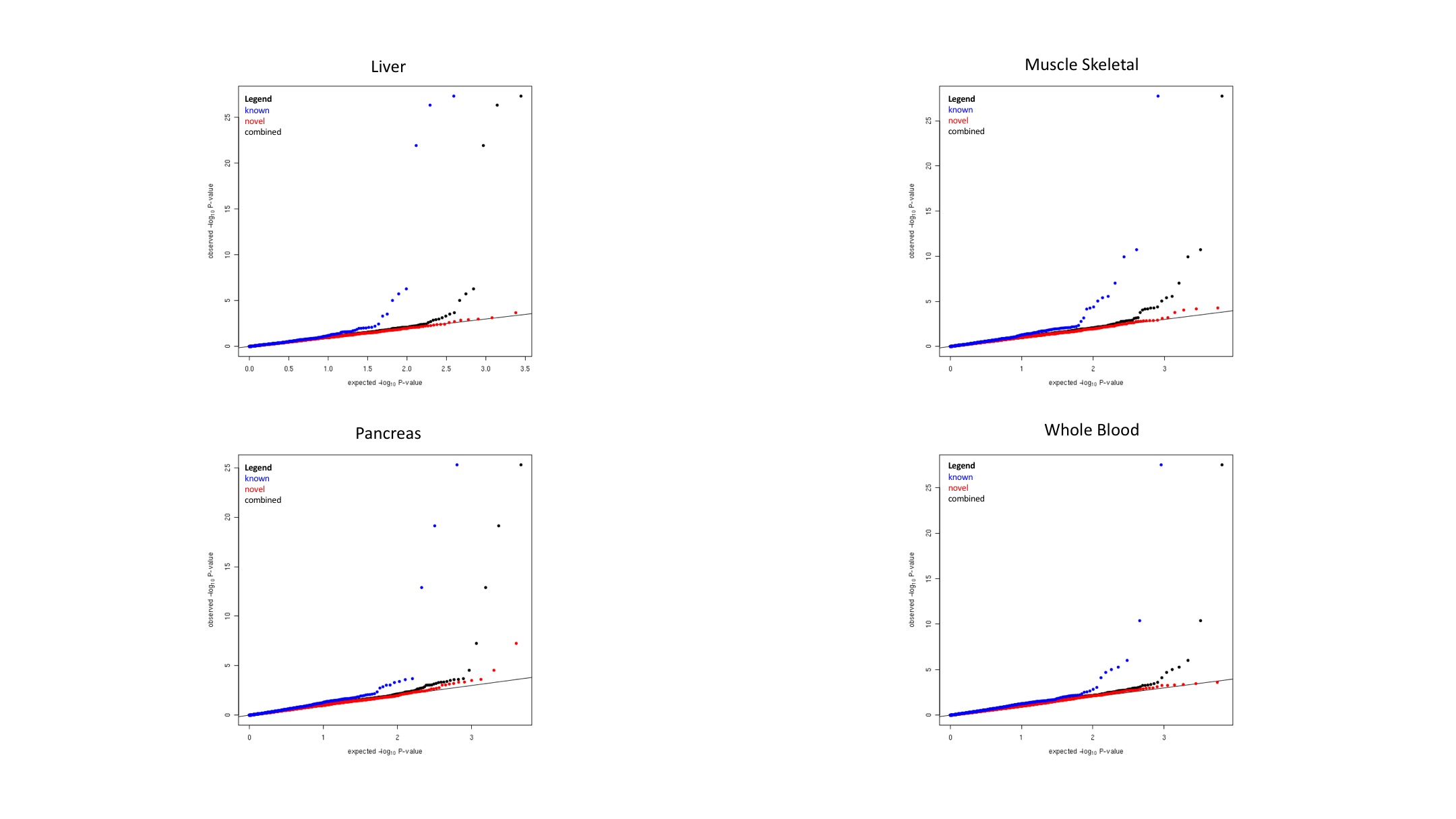


k) Platelet Count


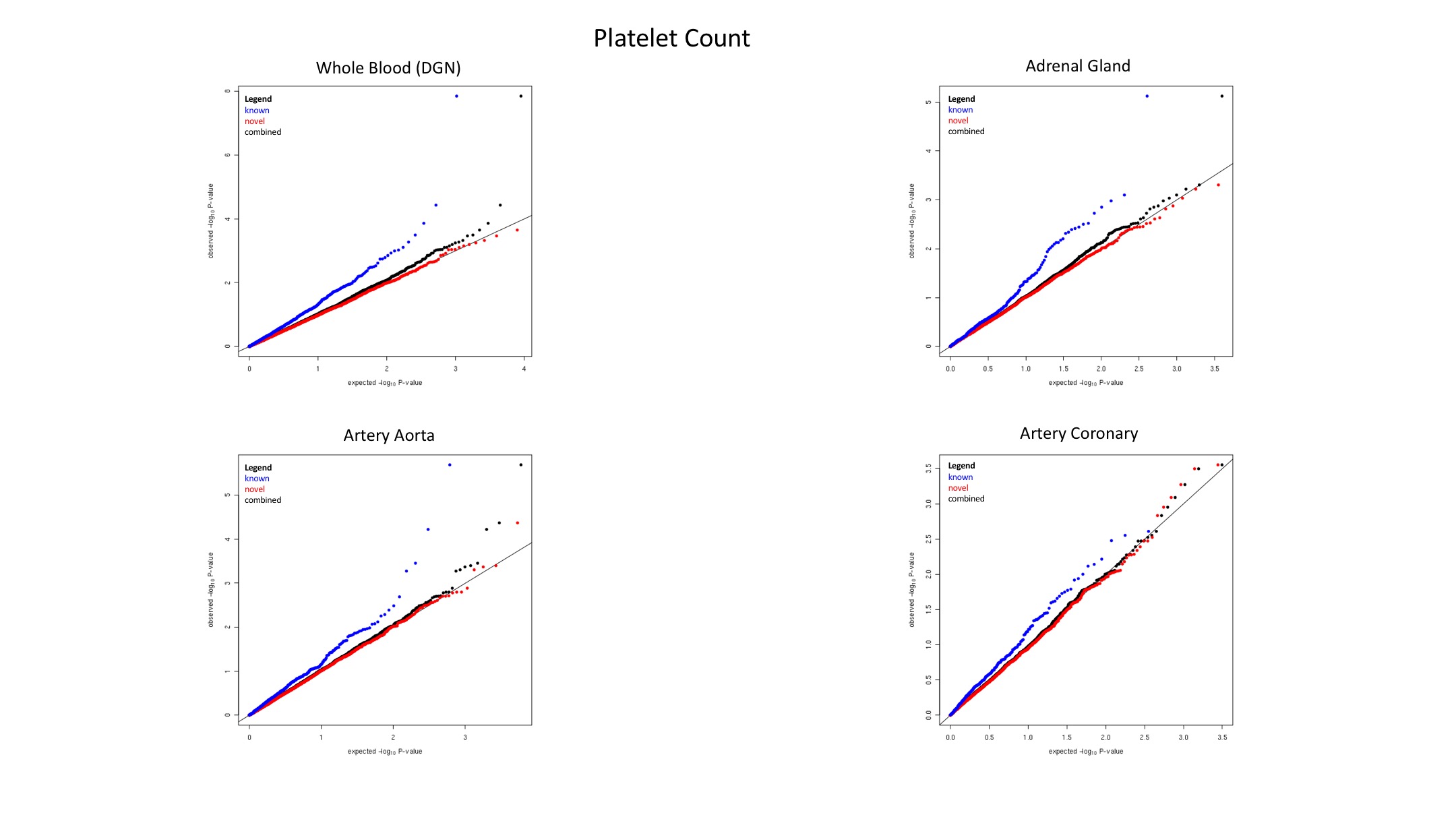


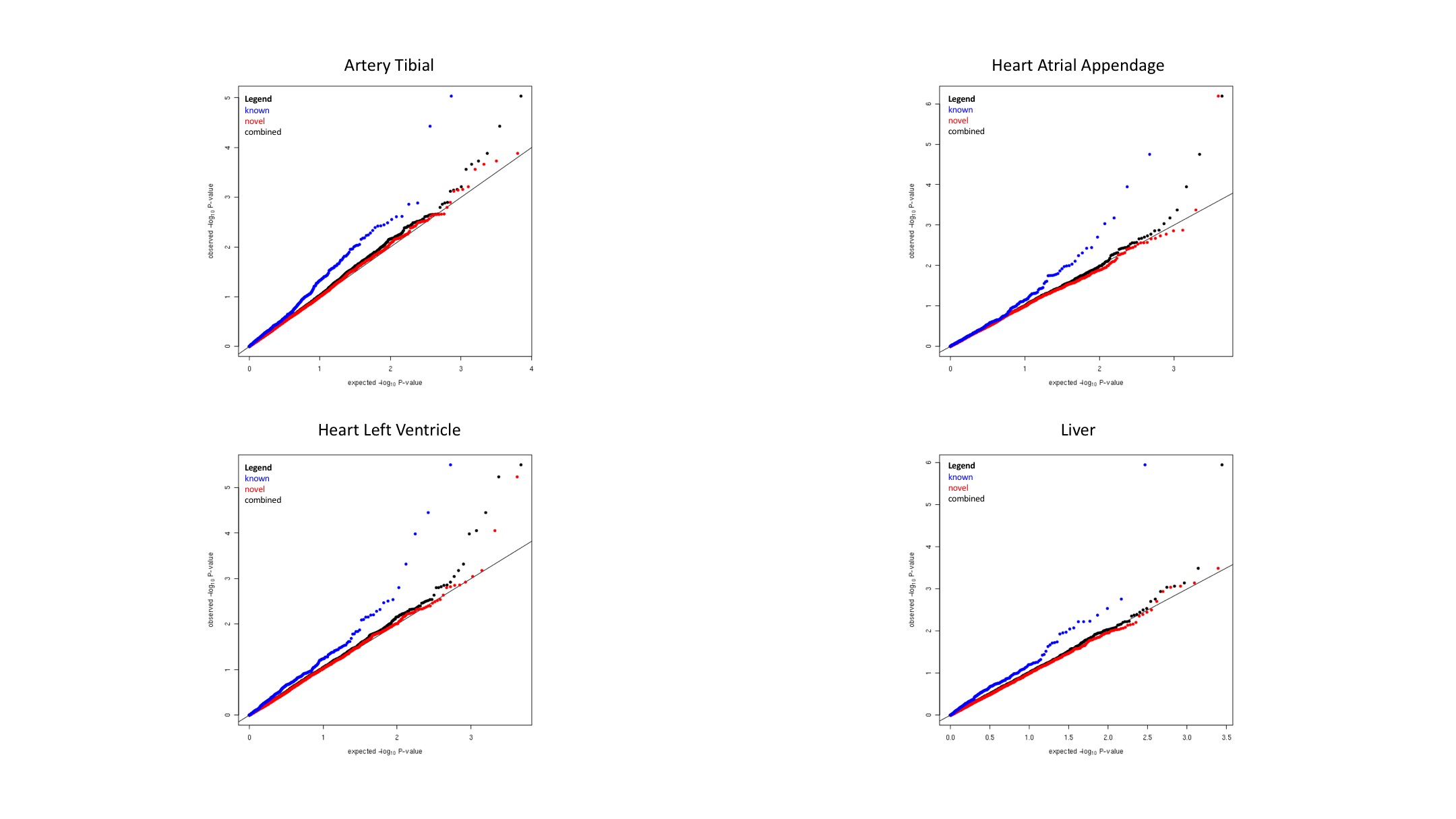


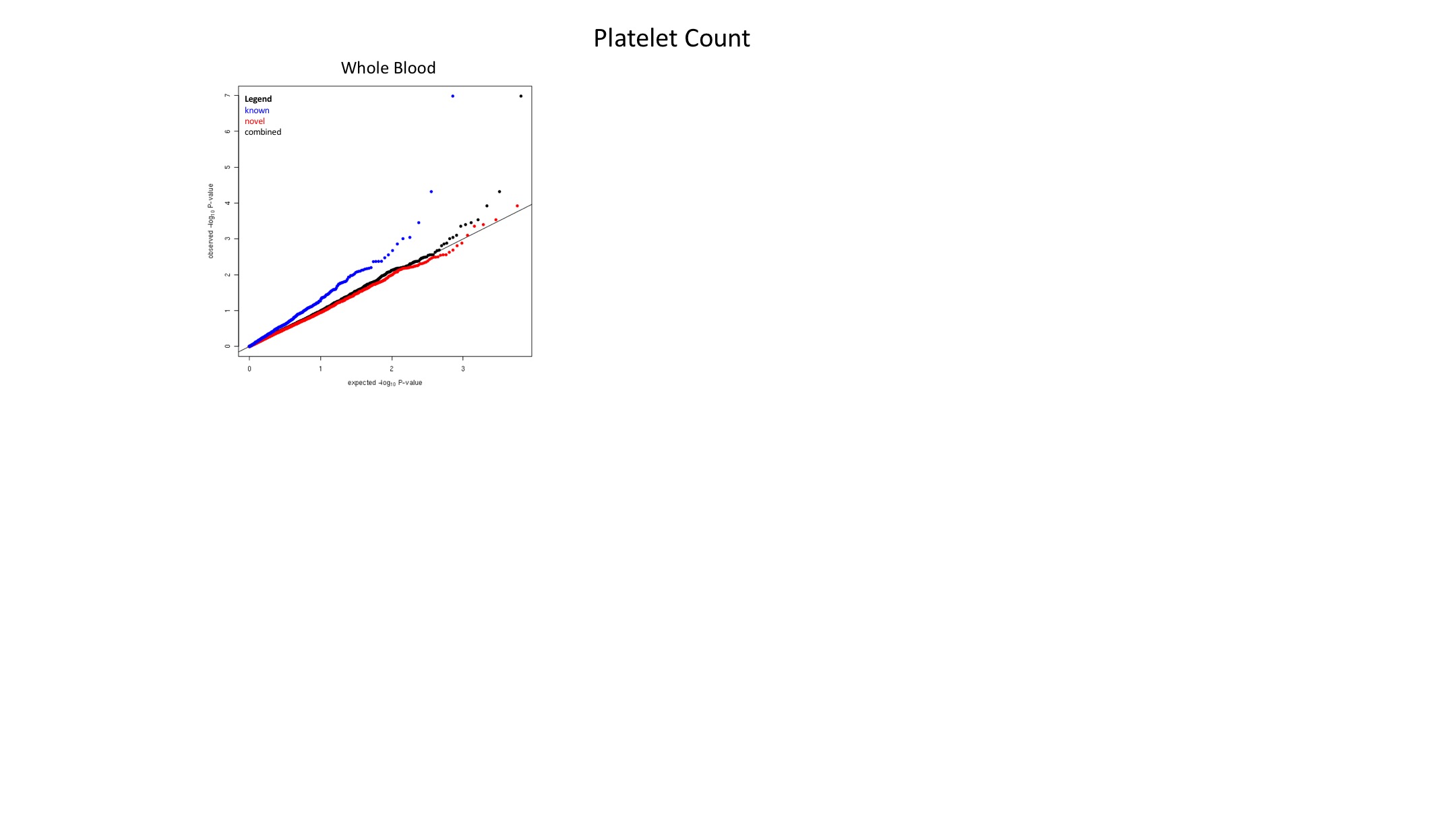


l) RR interval


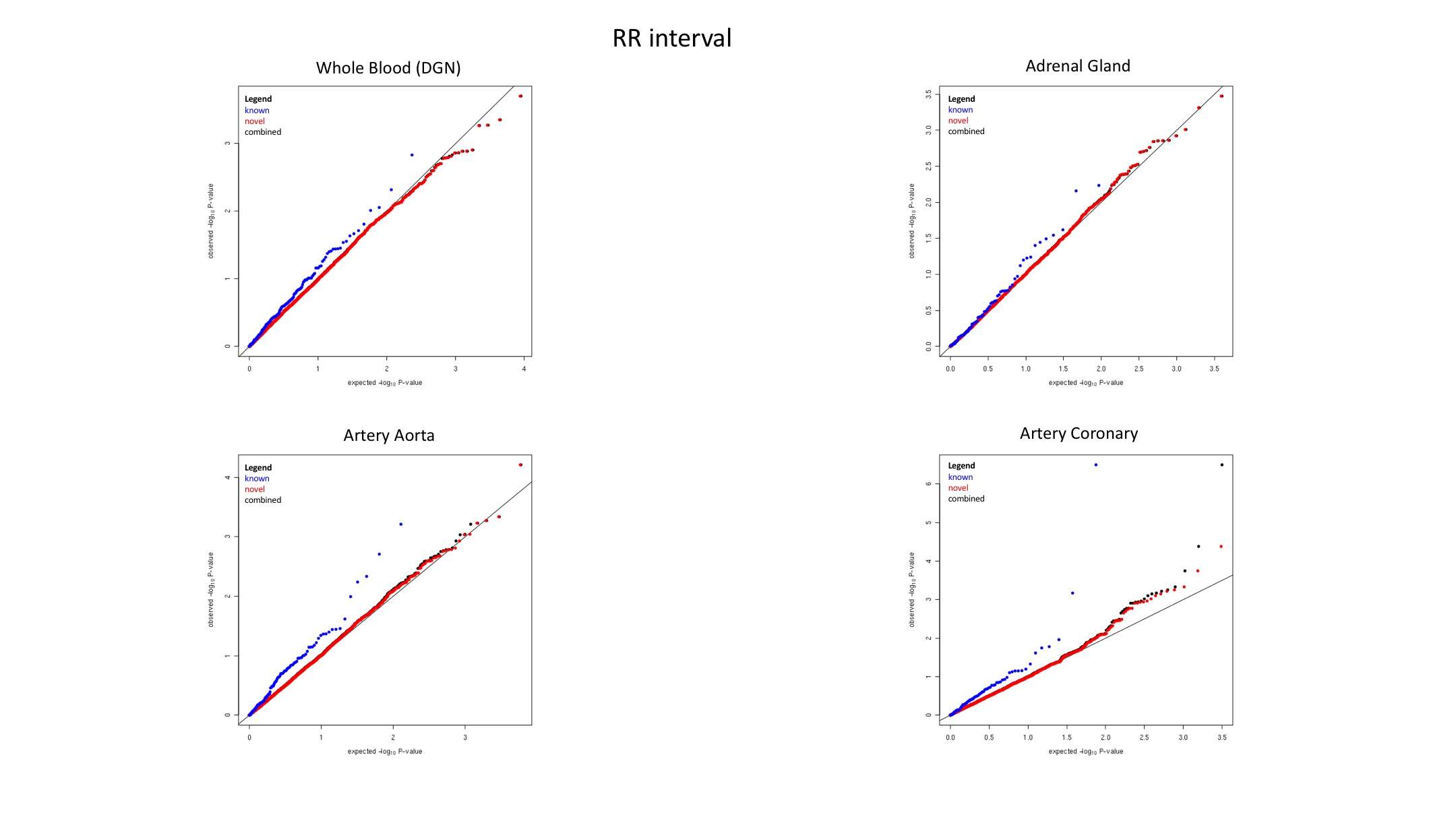


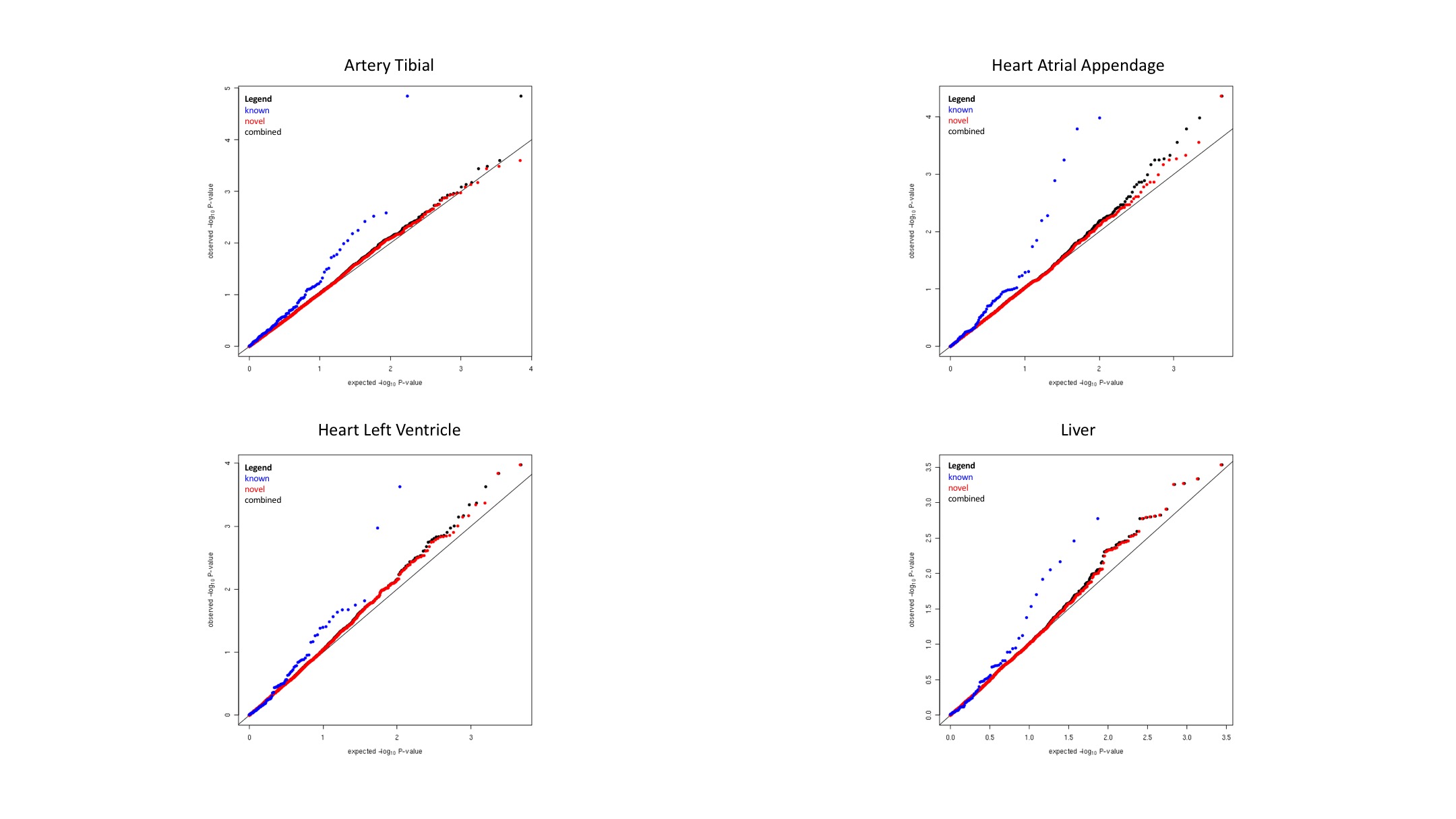


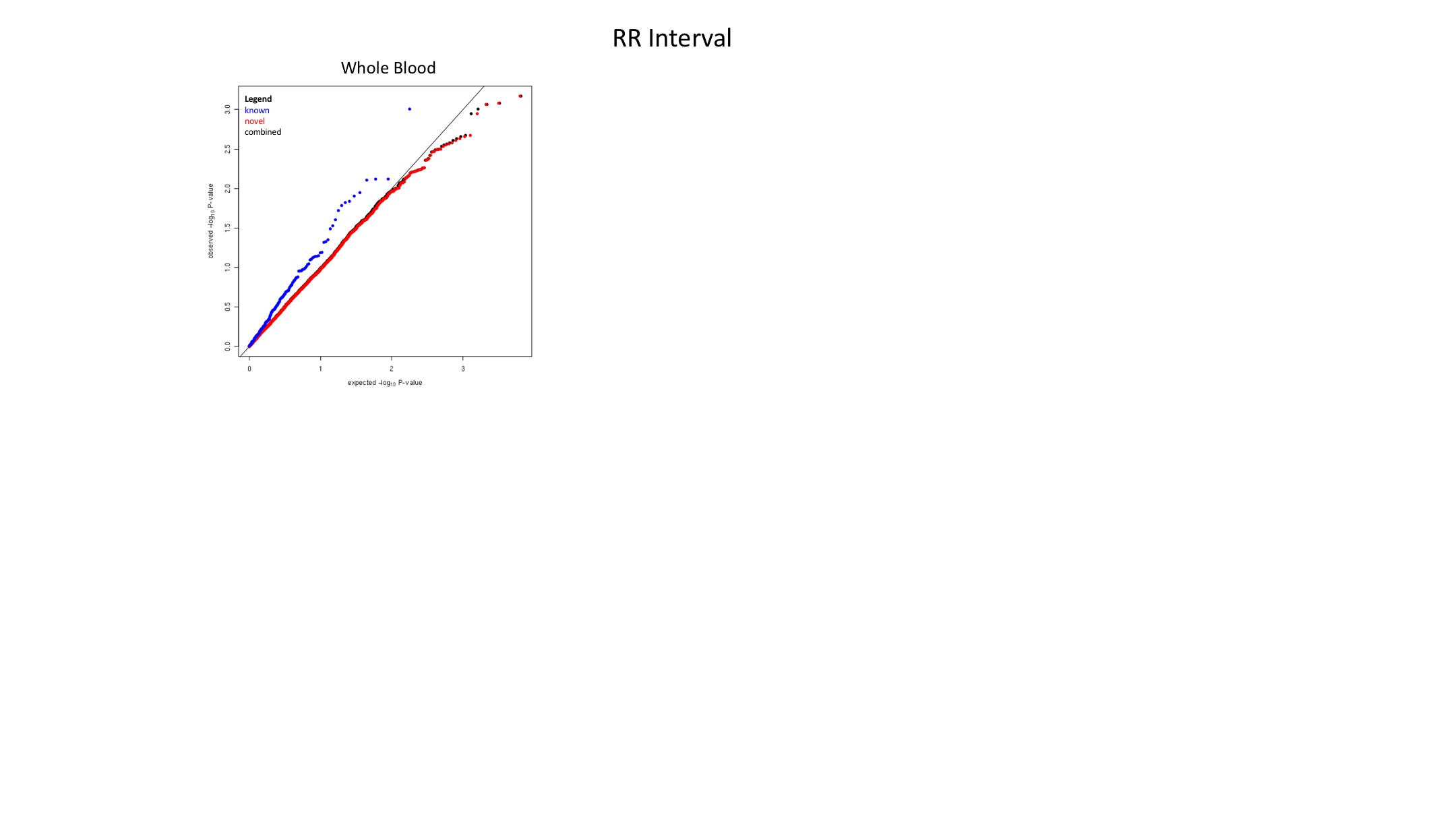


m) Systolic Blood Pressure


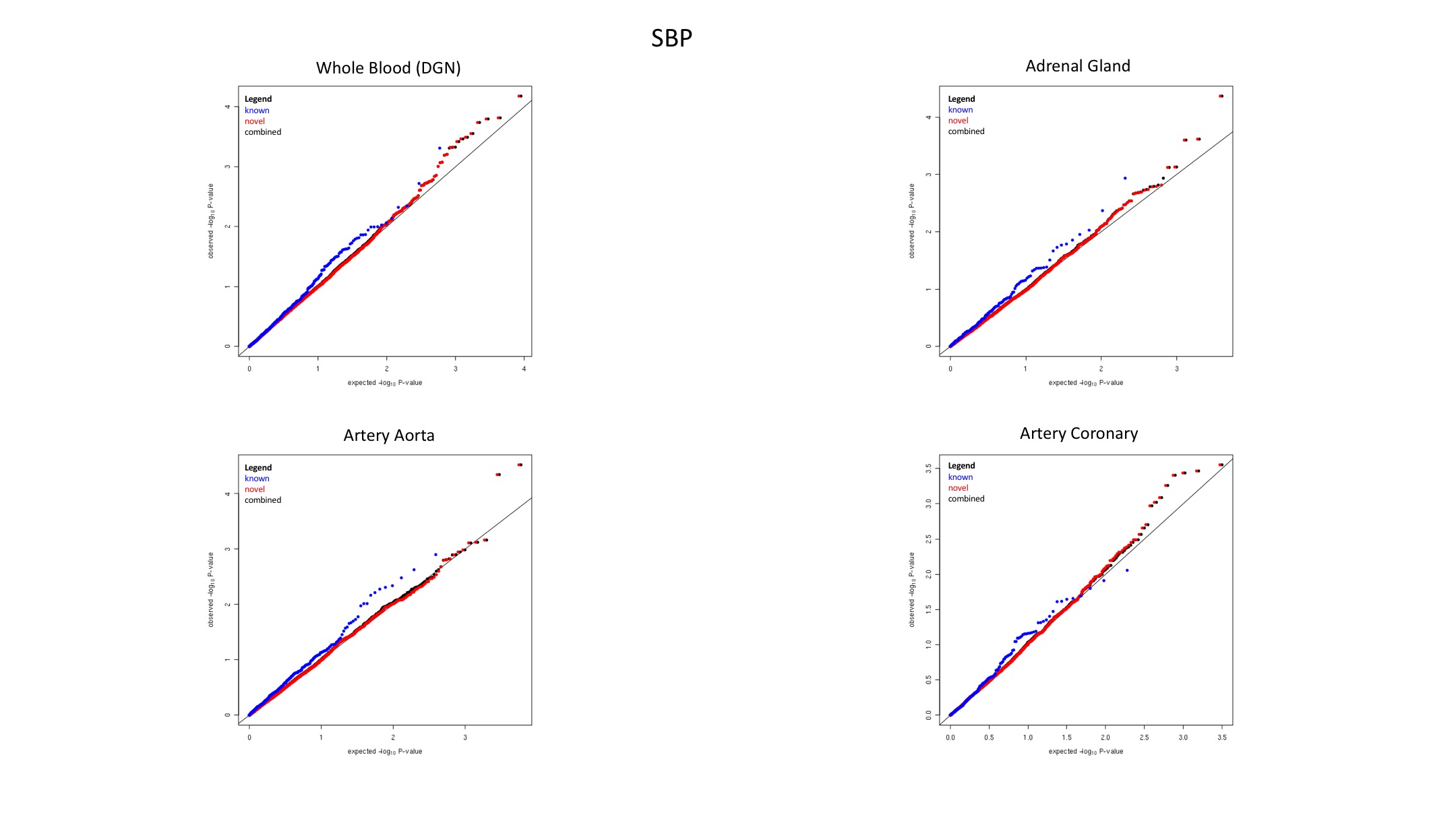


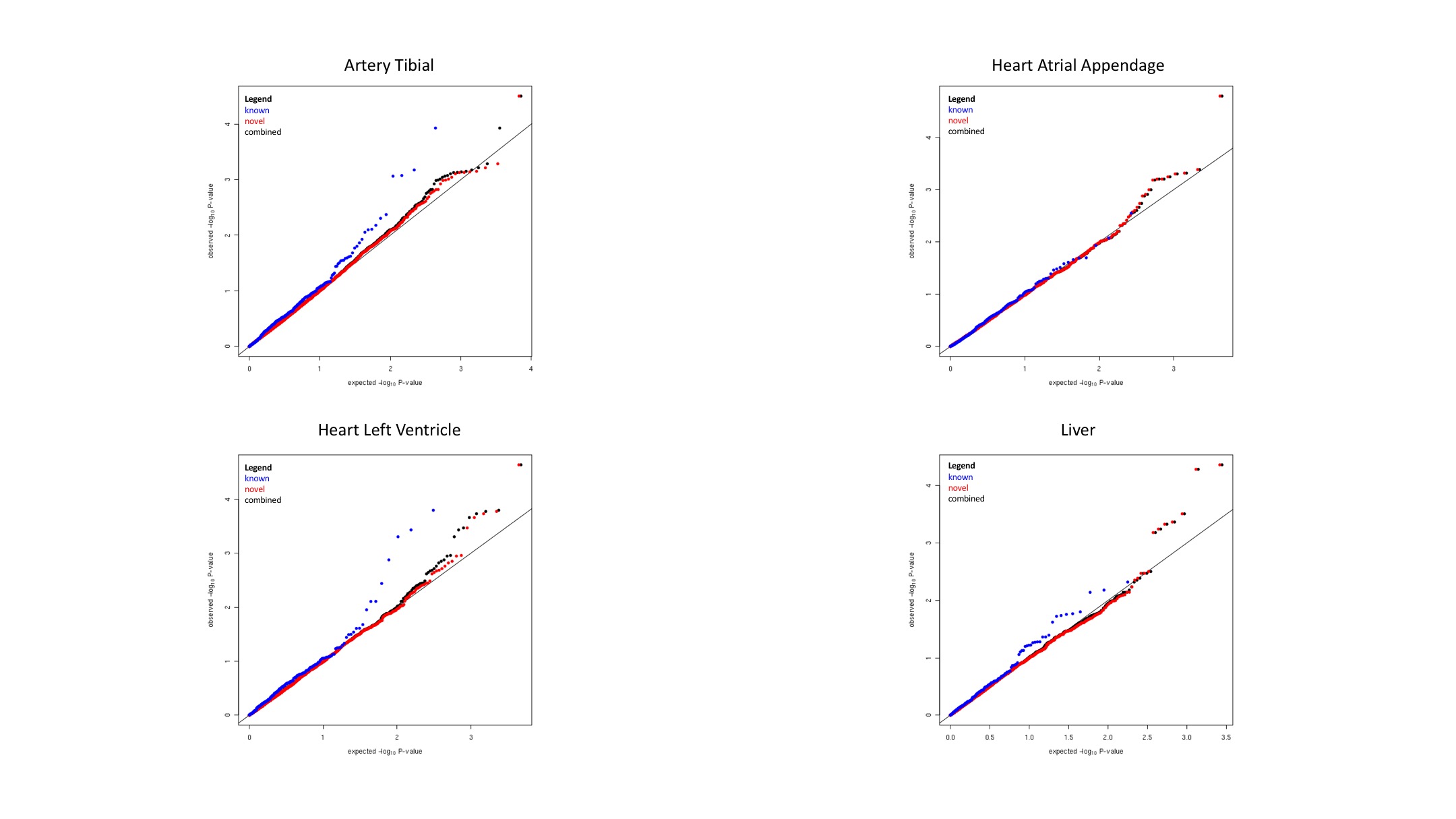


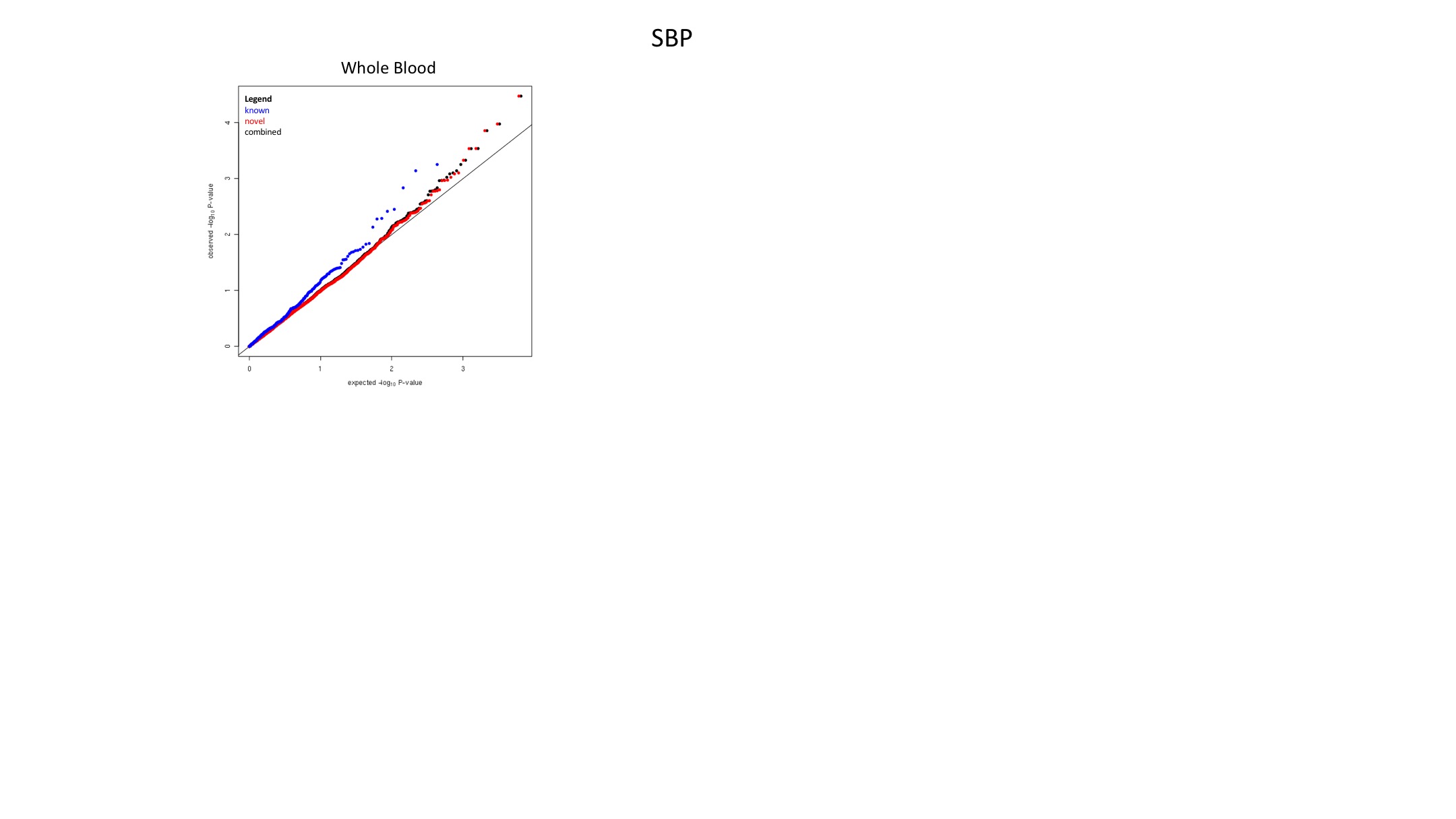


n) Triglycerides


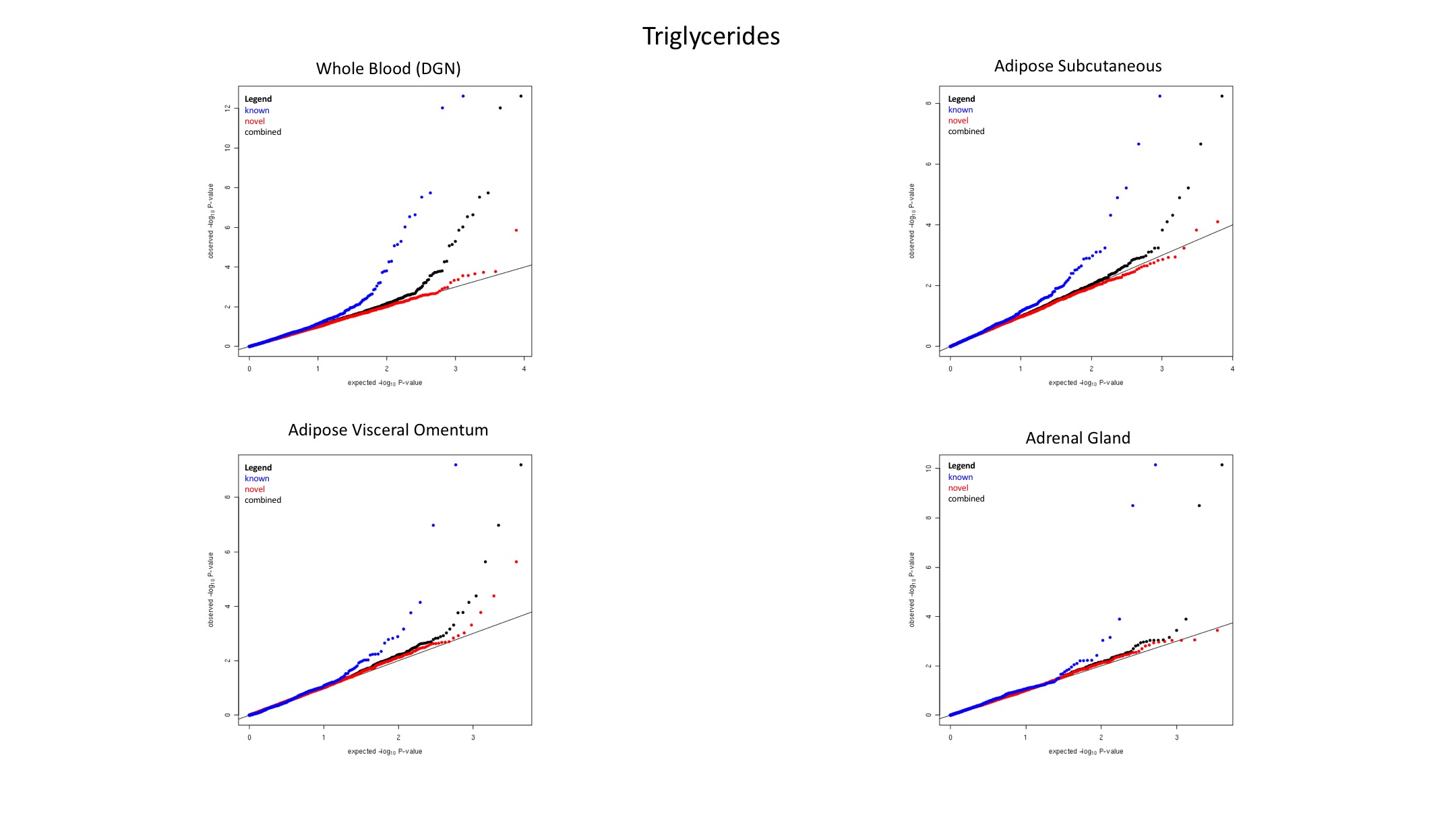

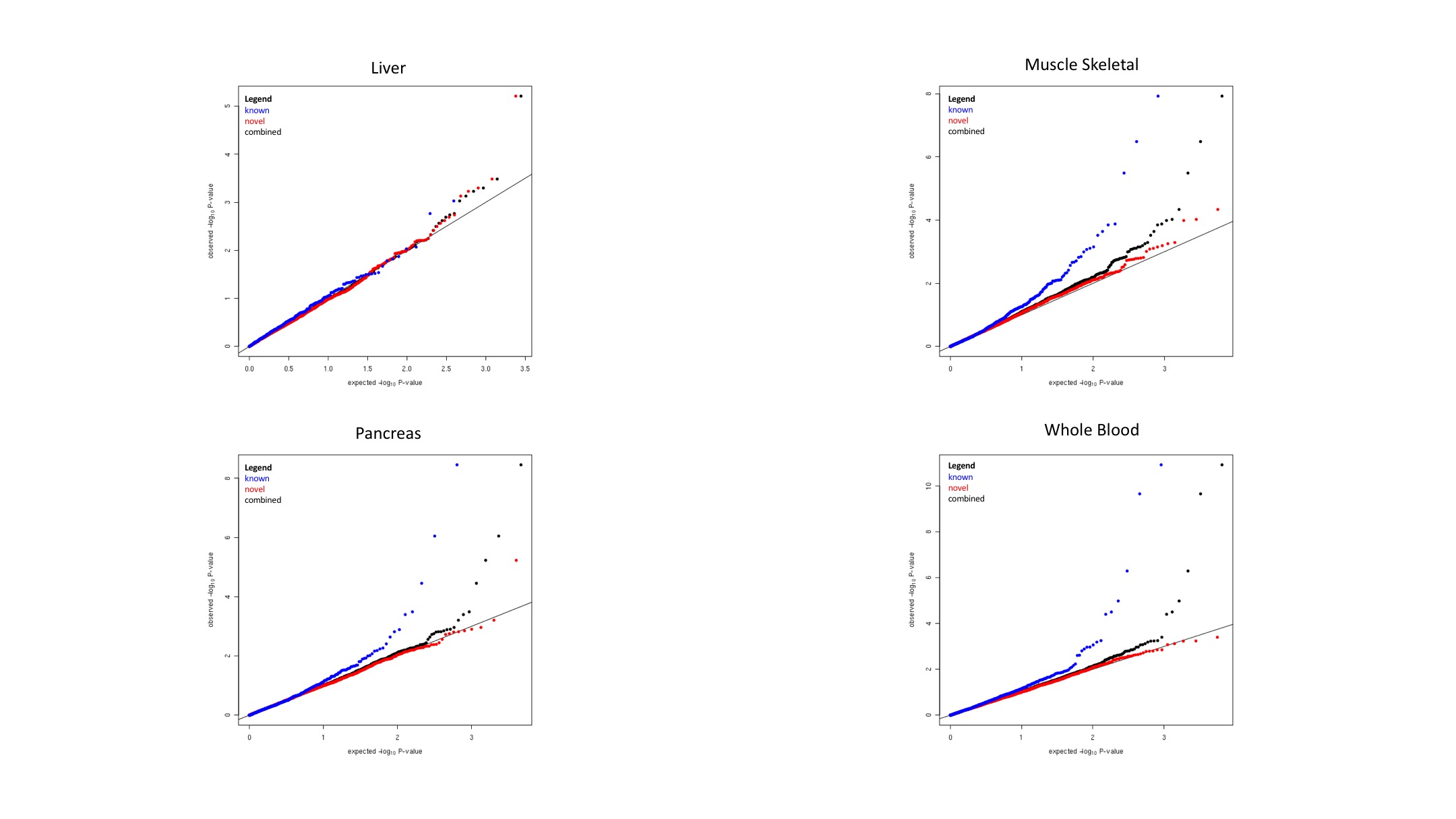


o) White Blood Cell Count


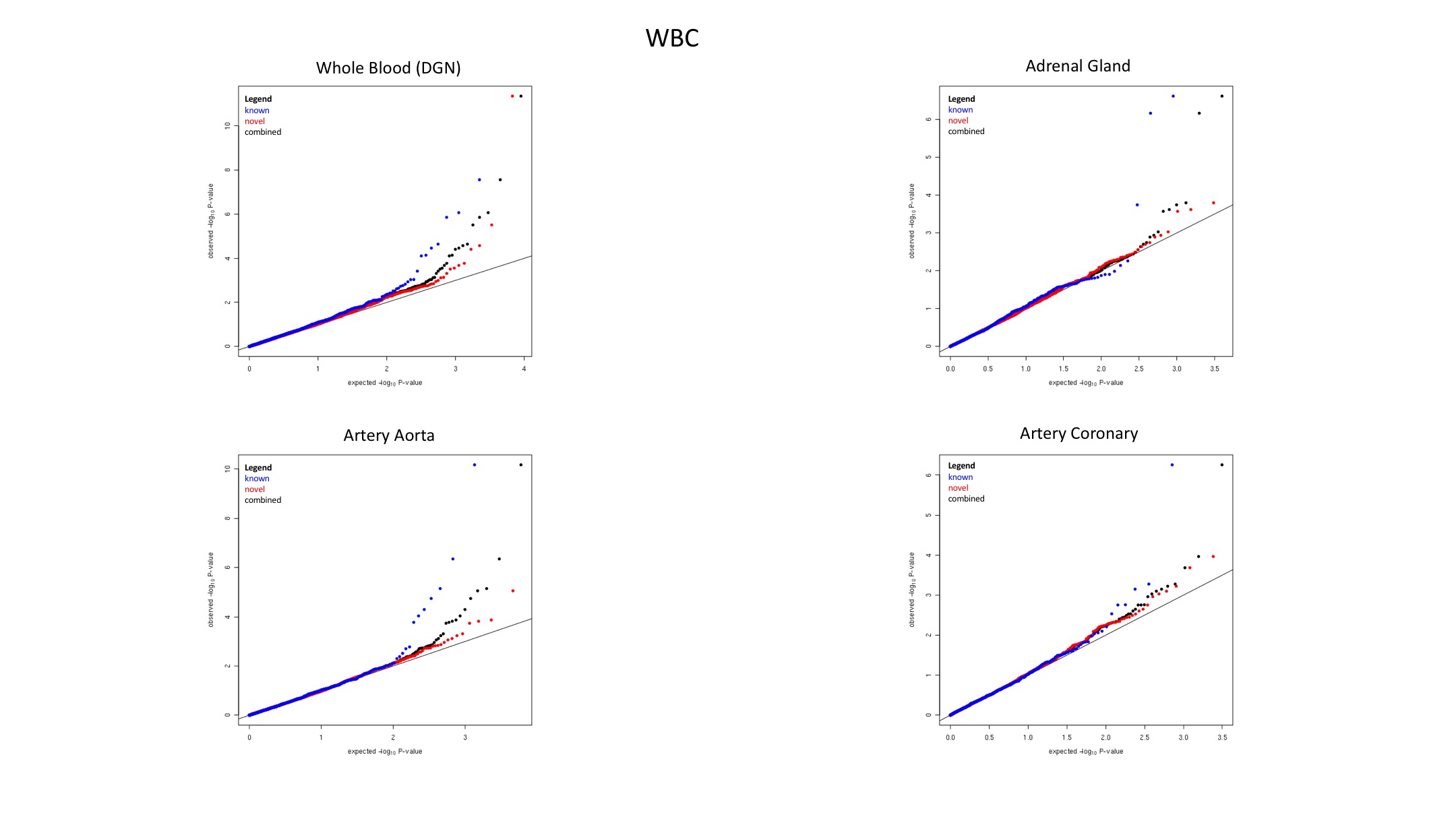

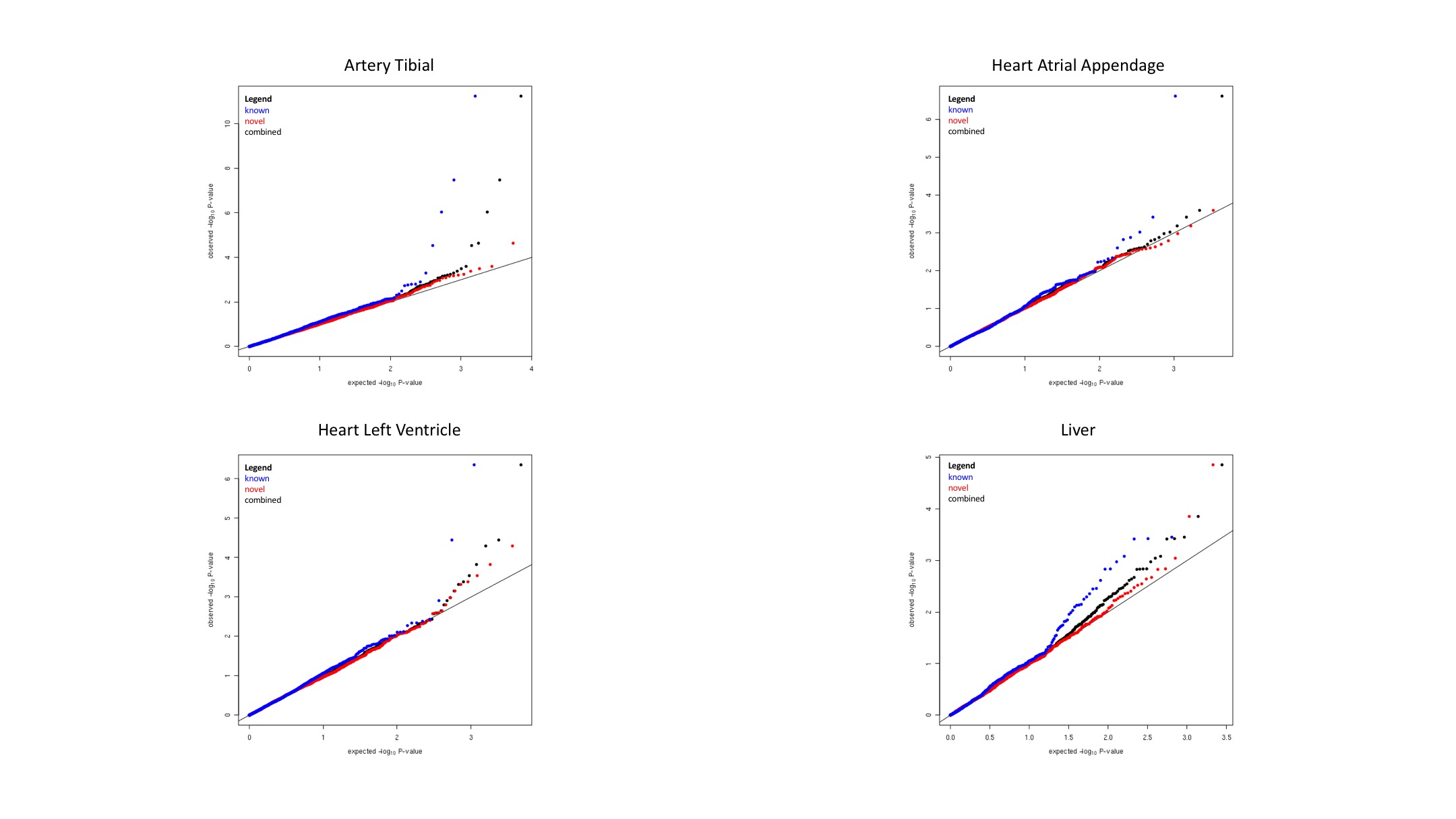


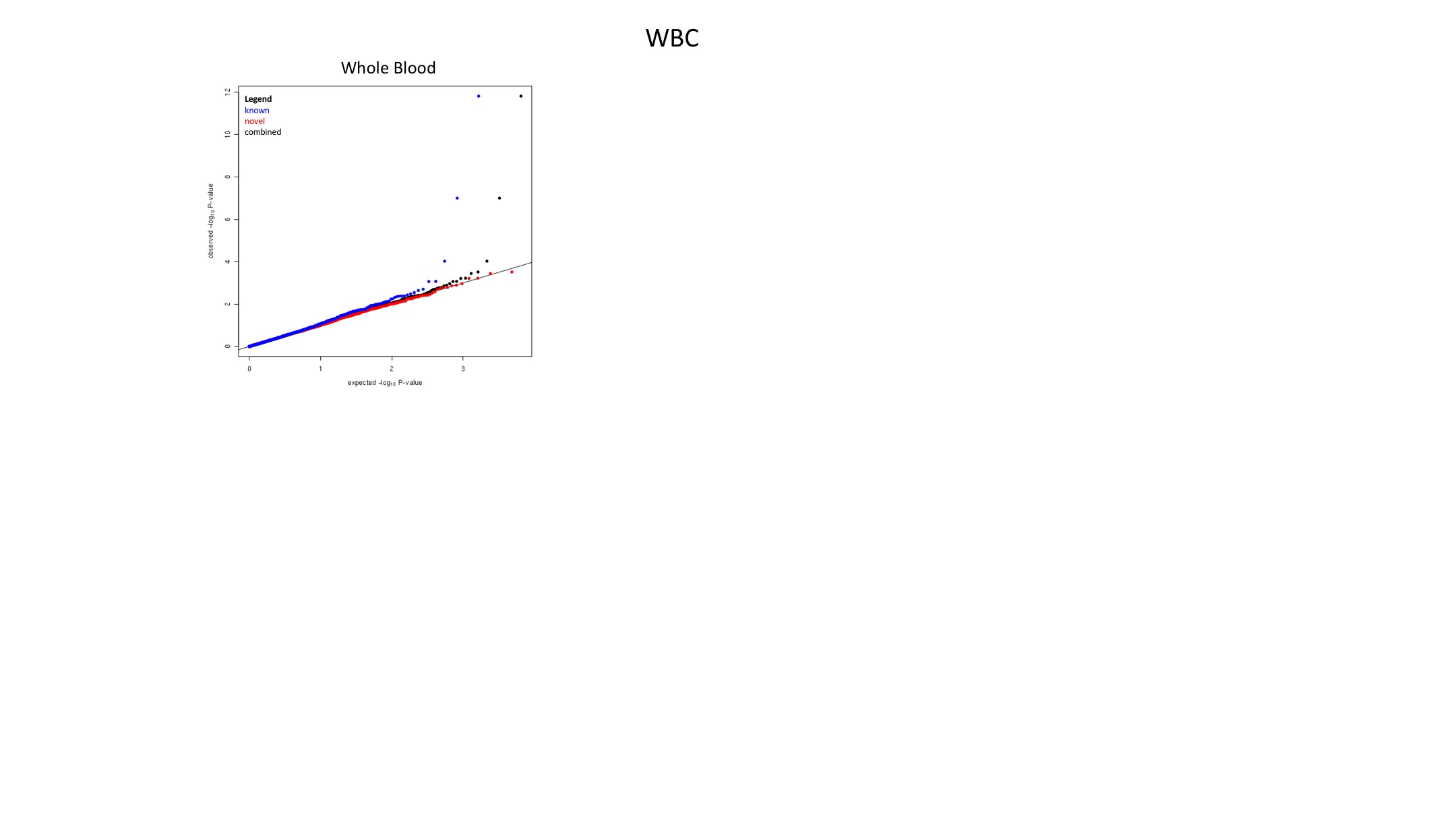


Supplementary Figure 7: Z-scores for novel gene GReX-trait association across relevant tissues for each trait

a) Body mass index

b) Fasting glucose

c) Fasting insulin

d) Height

e) HDL cholesterol

f) LDL cholesterol

g) Total cholesterol

h) Triglycerides

i) Platelet count

j) White blood cell count

k) Diastolic blood pressure

l) Systolic blood pressure

m) Factor VII

n) RR interval

Supplementary Figure 8: QQ plots for each trait

a) Body mass index


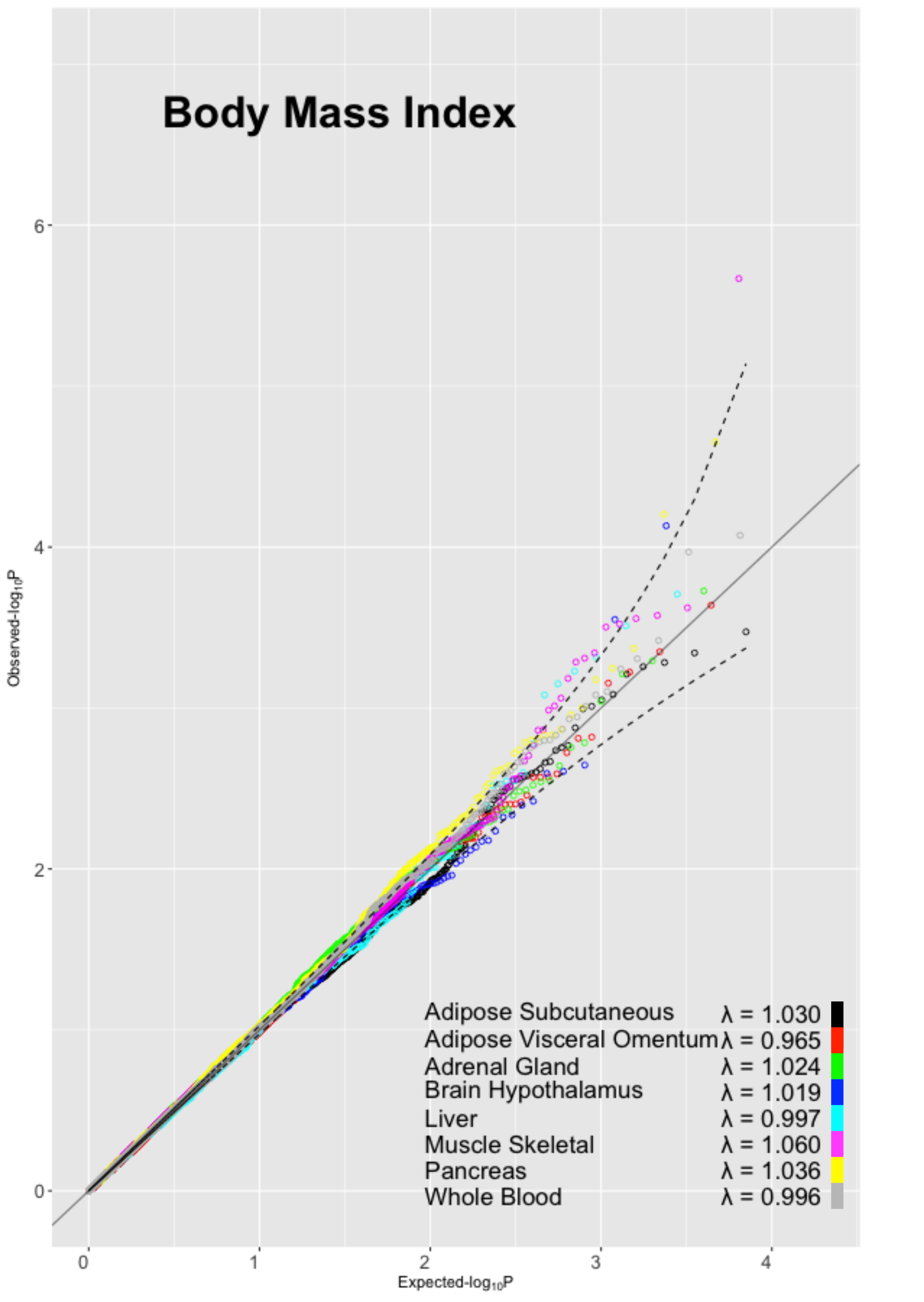


b) Fasting glucose


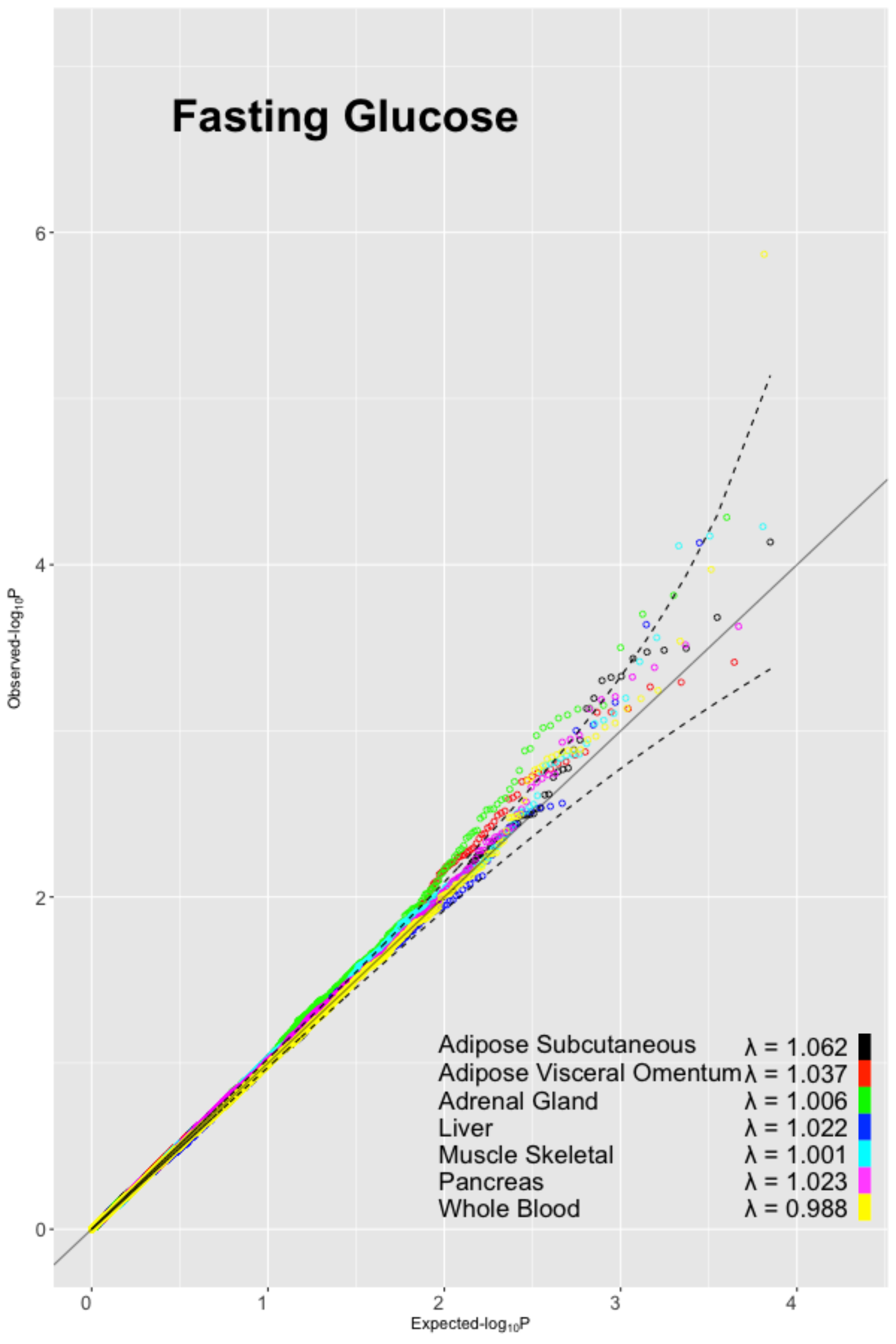


c) Fasting insulin


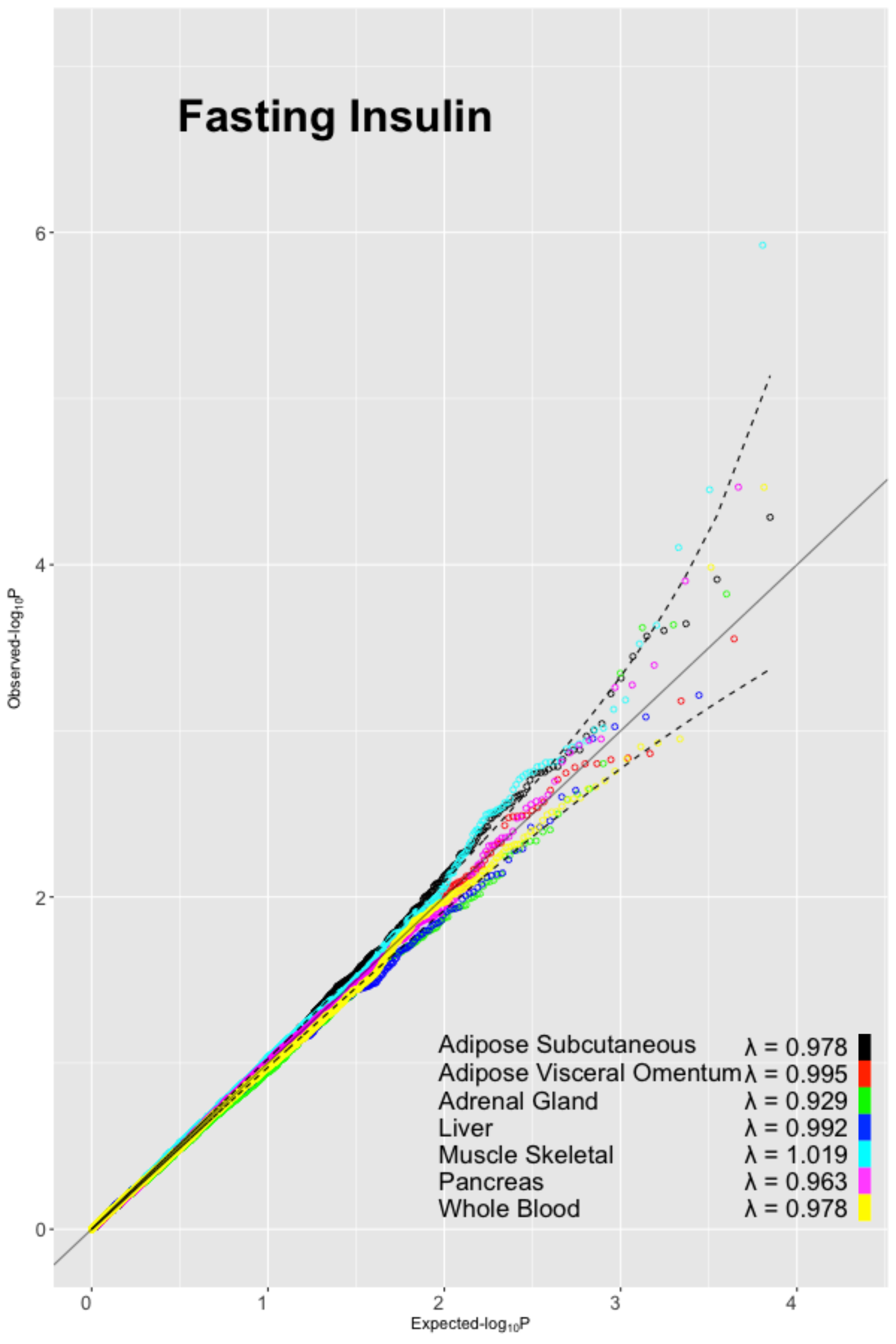


d) Height


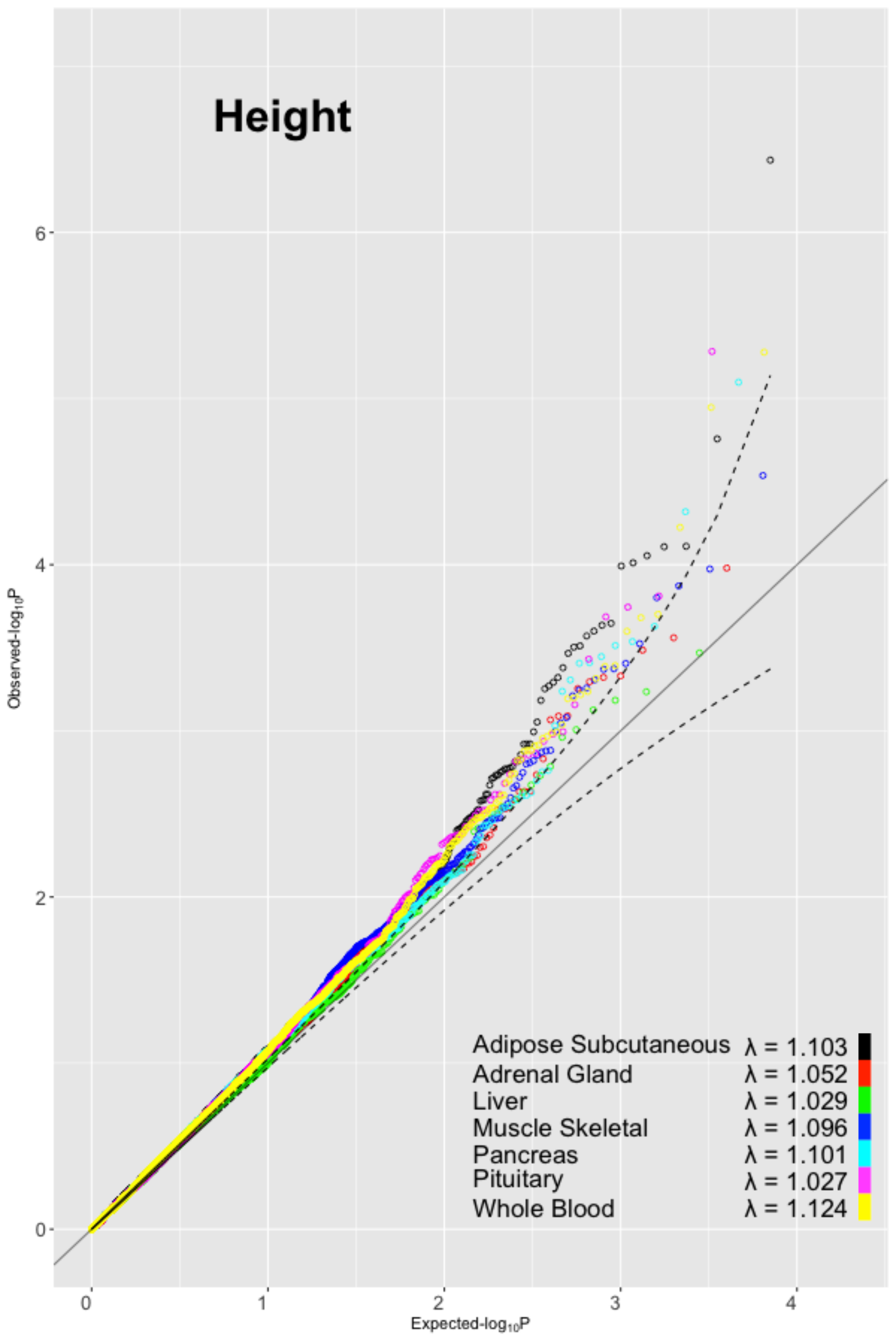


e) HDL cholesterol


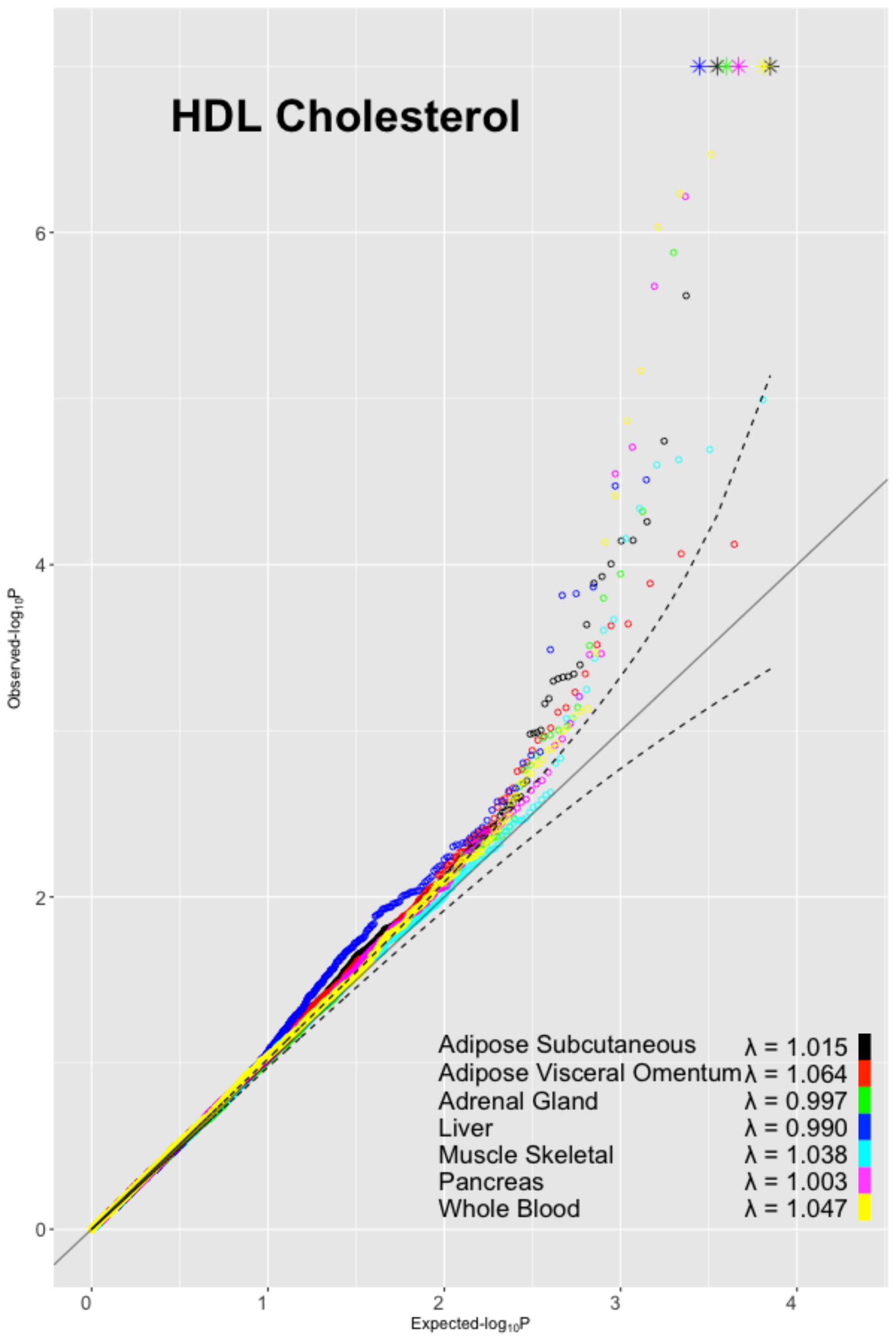


f) LDL cholesterol


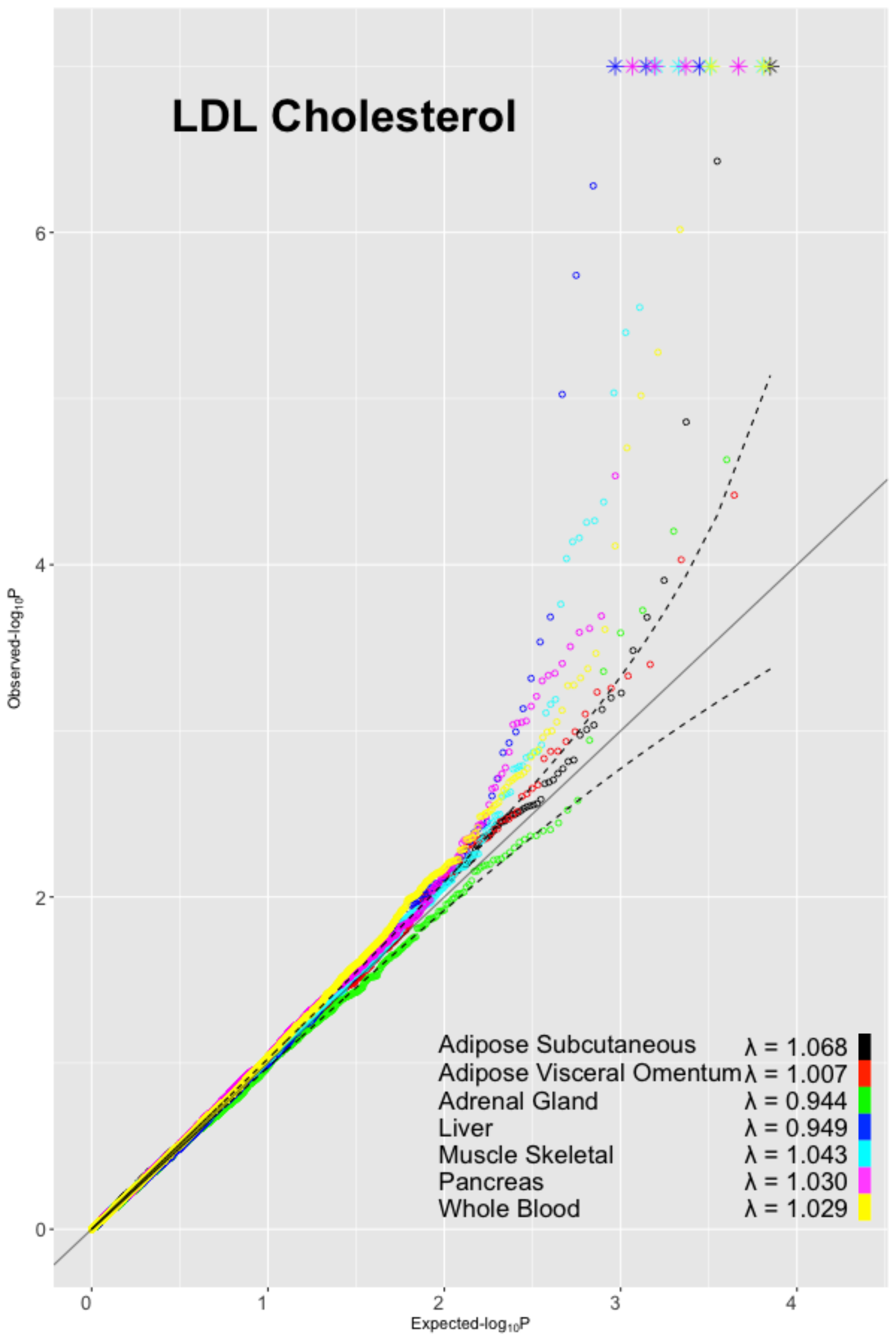


g) Total cholesterol


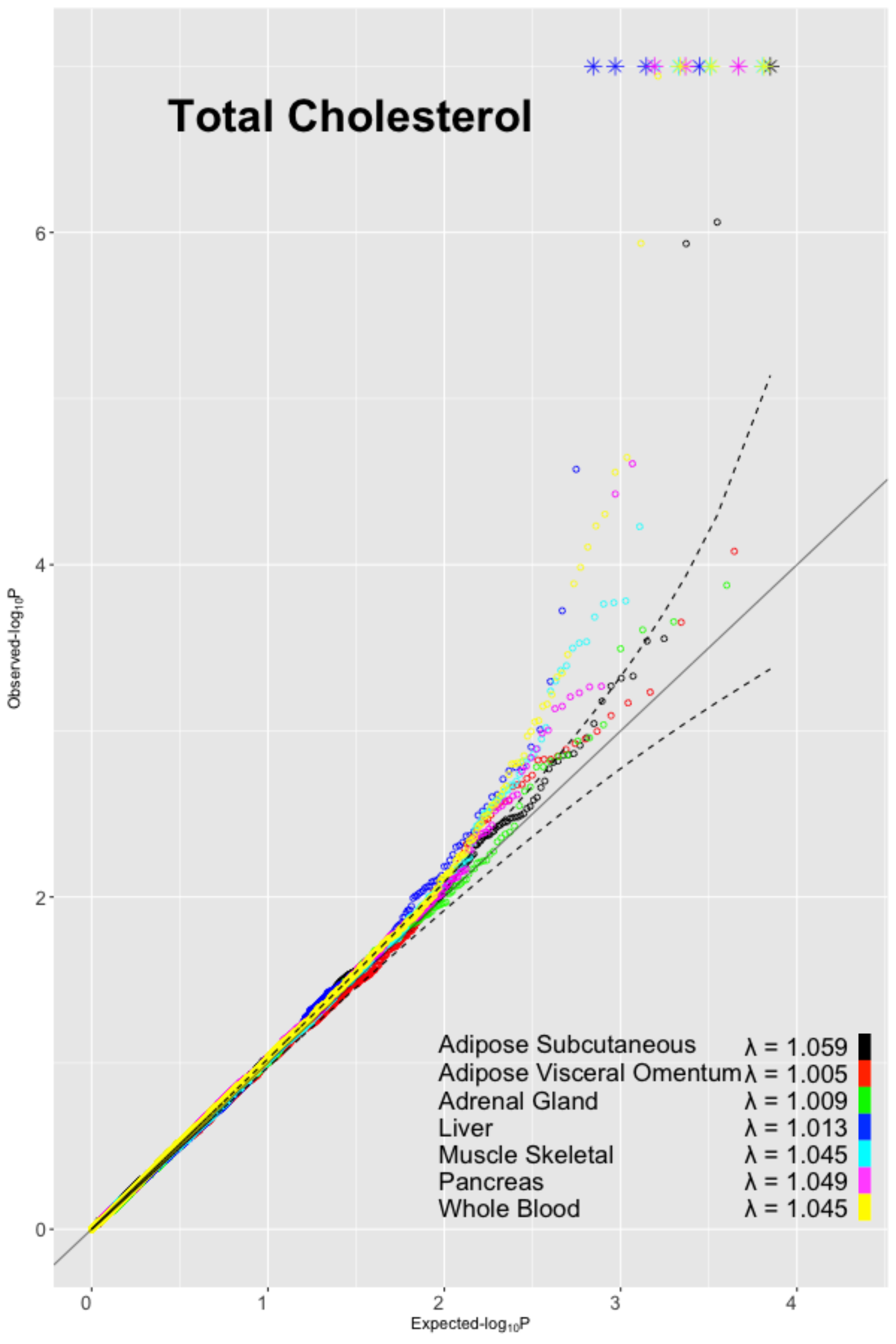


h) Triglycerides


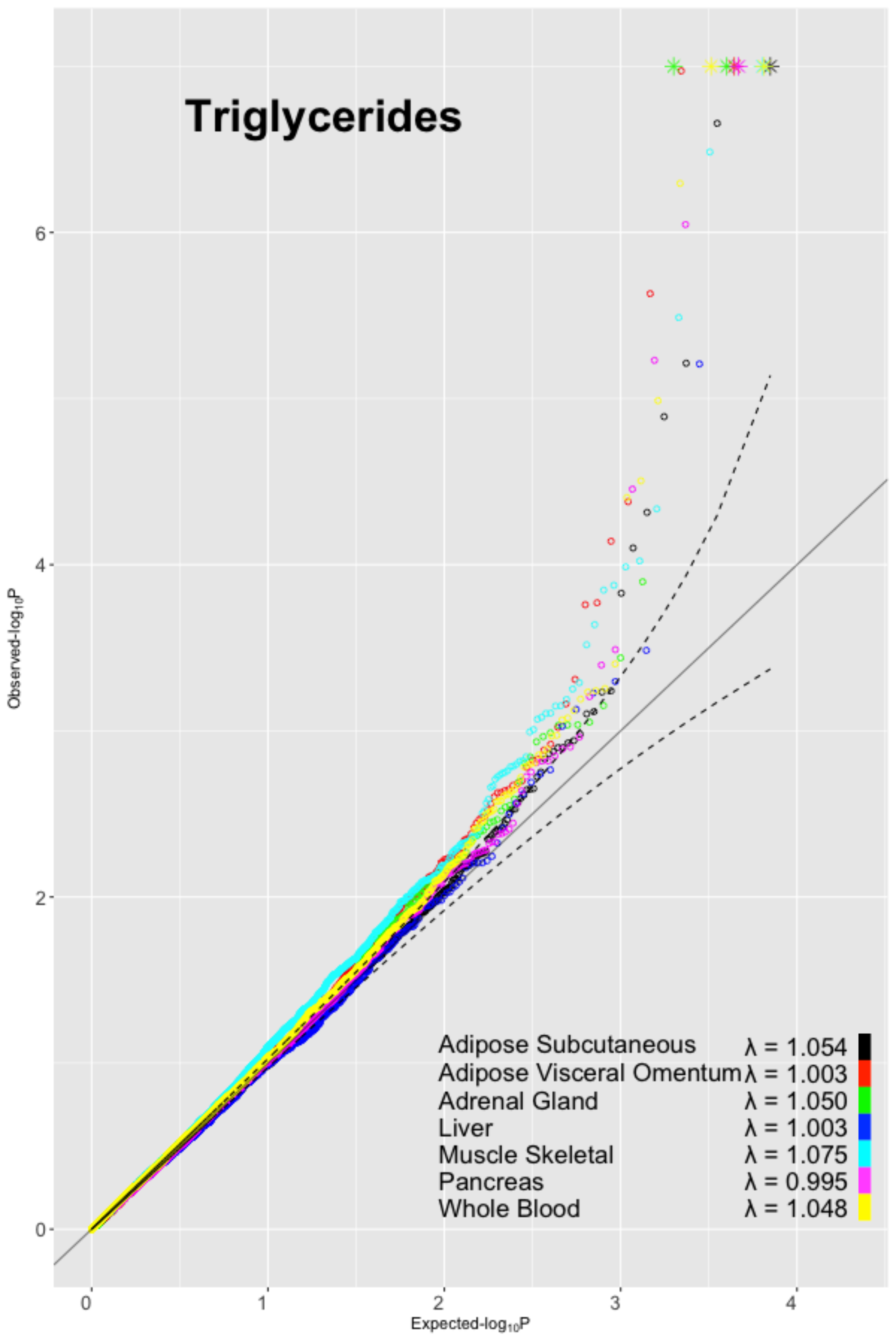


i) Platelet count


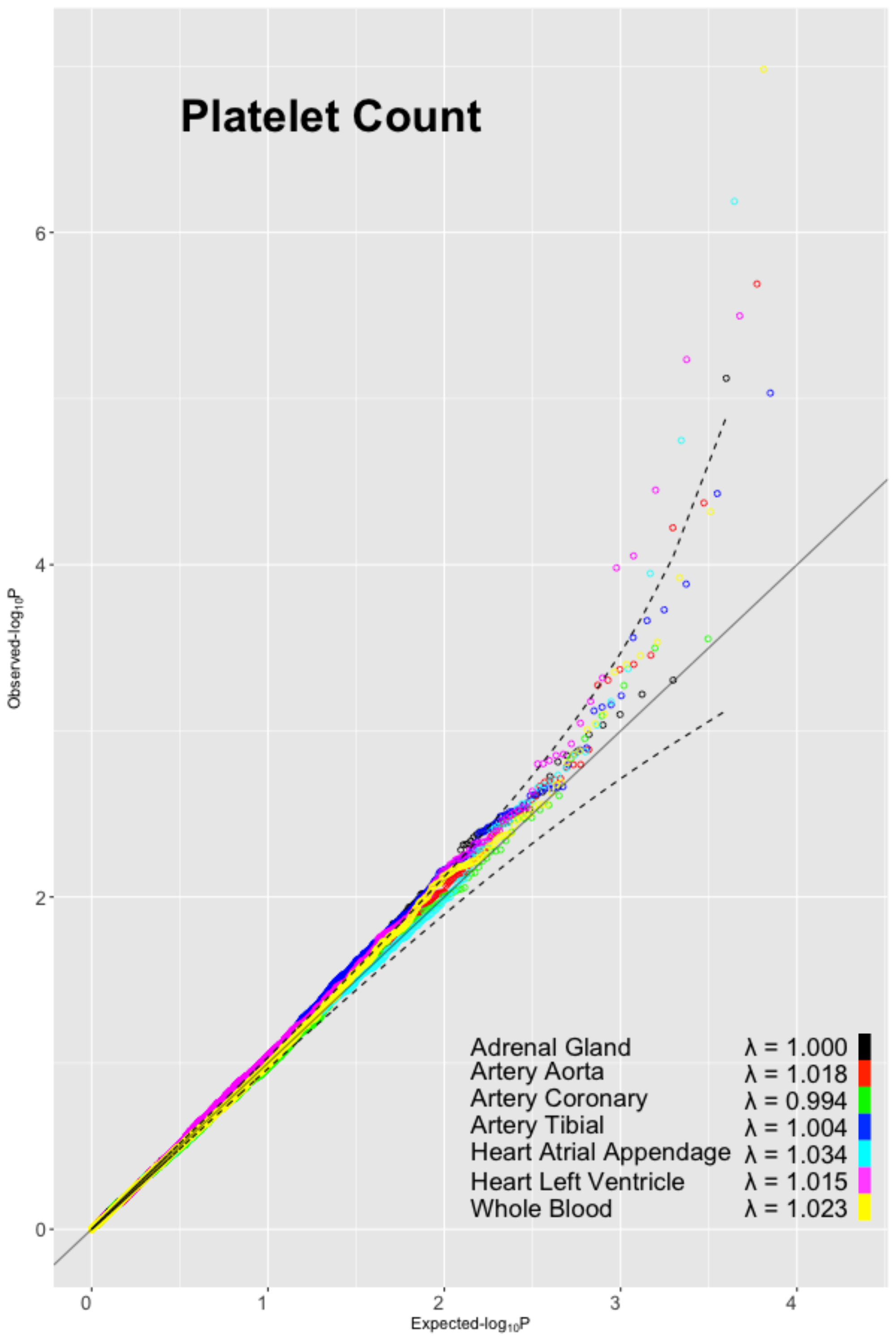


j) White blood cell count


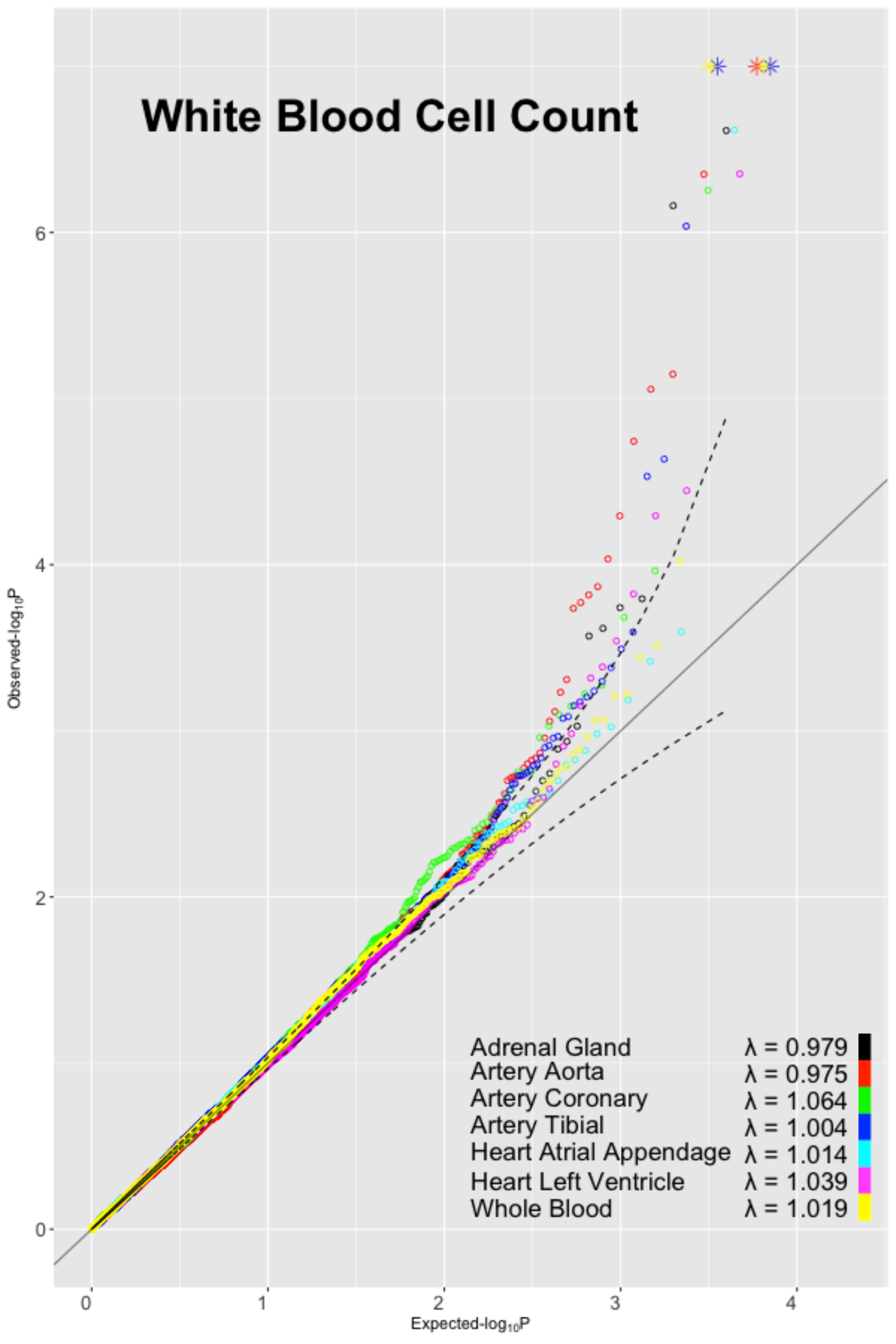


k) Diastolic blood pressure


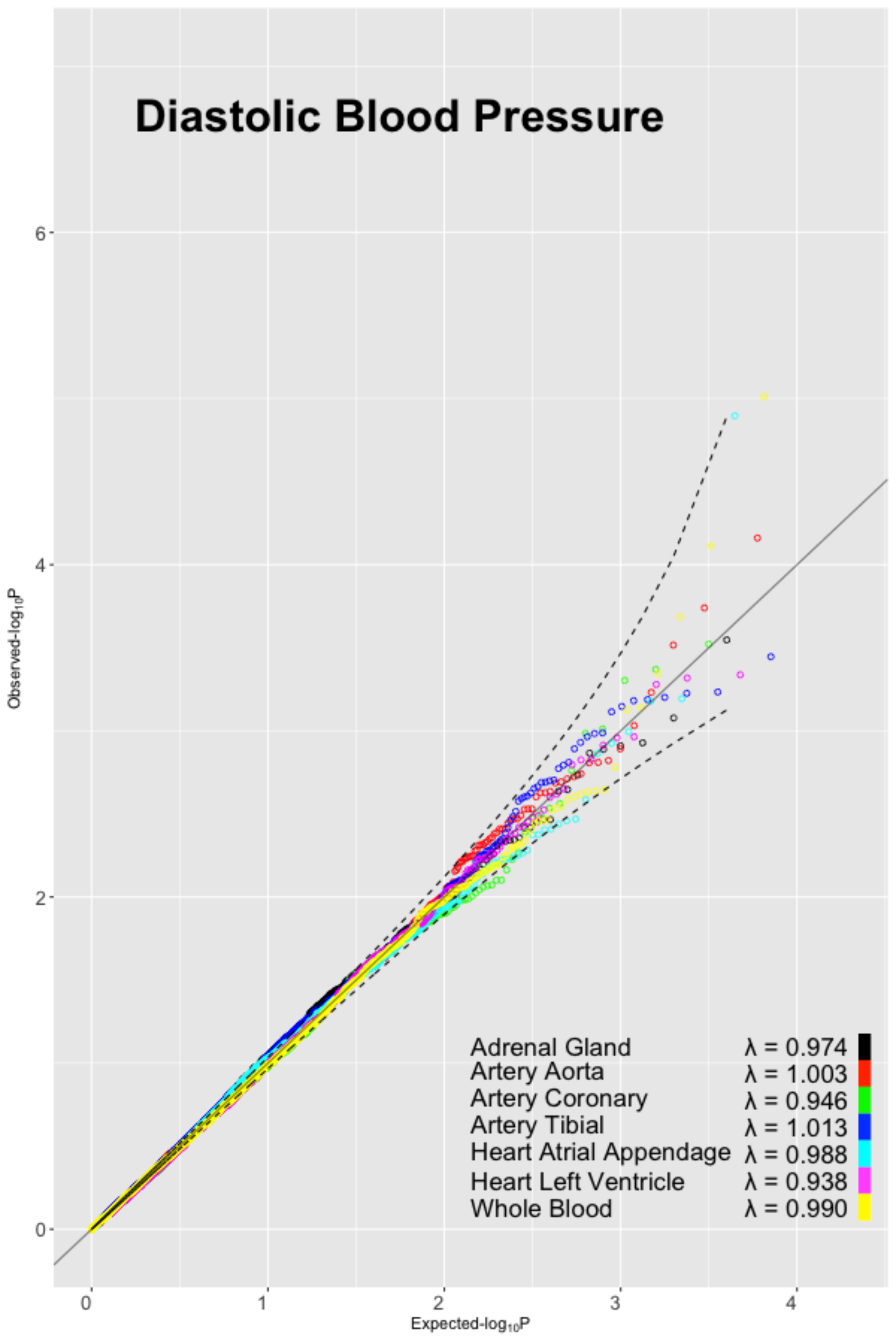


l) Systolic blood pressure


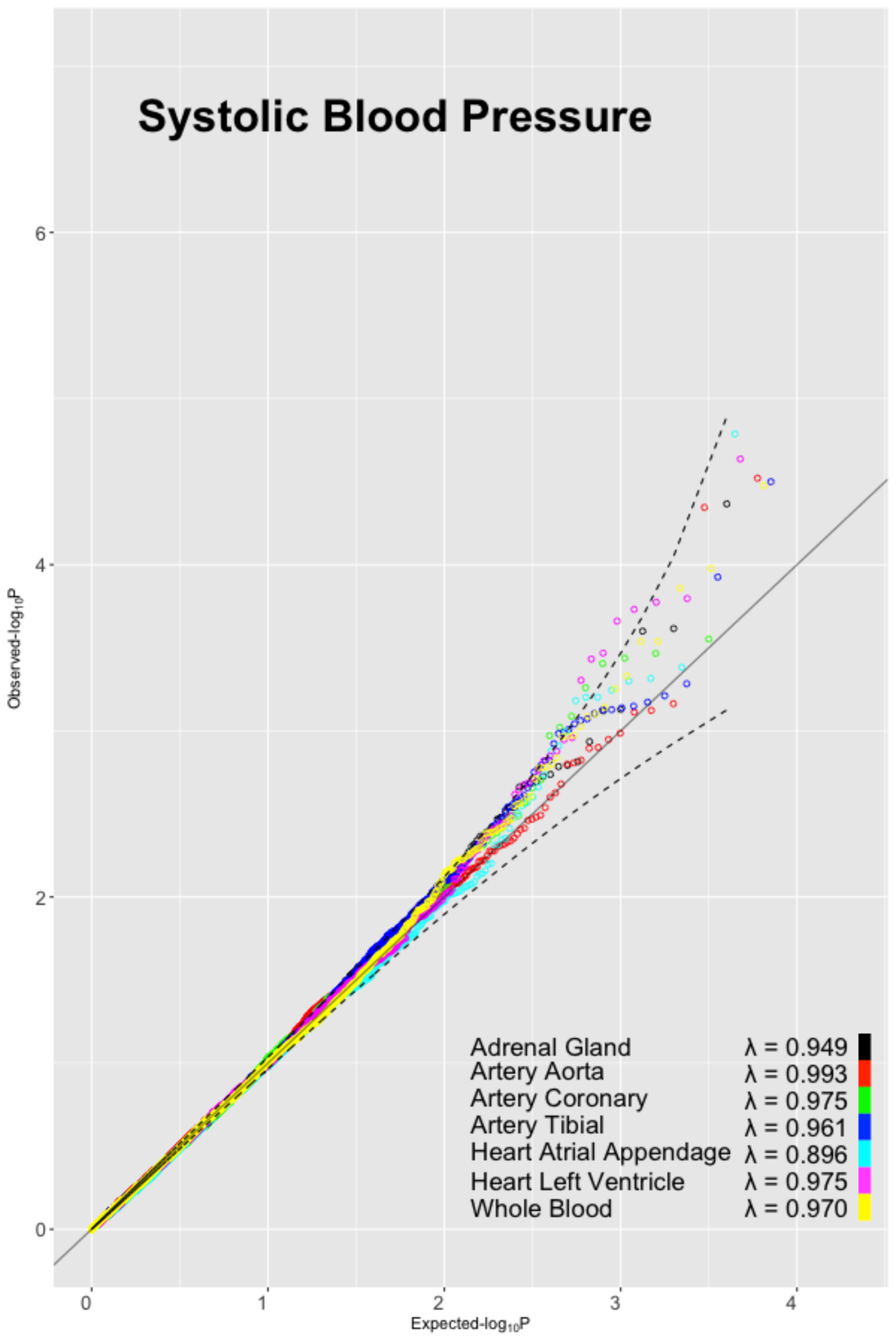


m) Factor VII


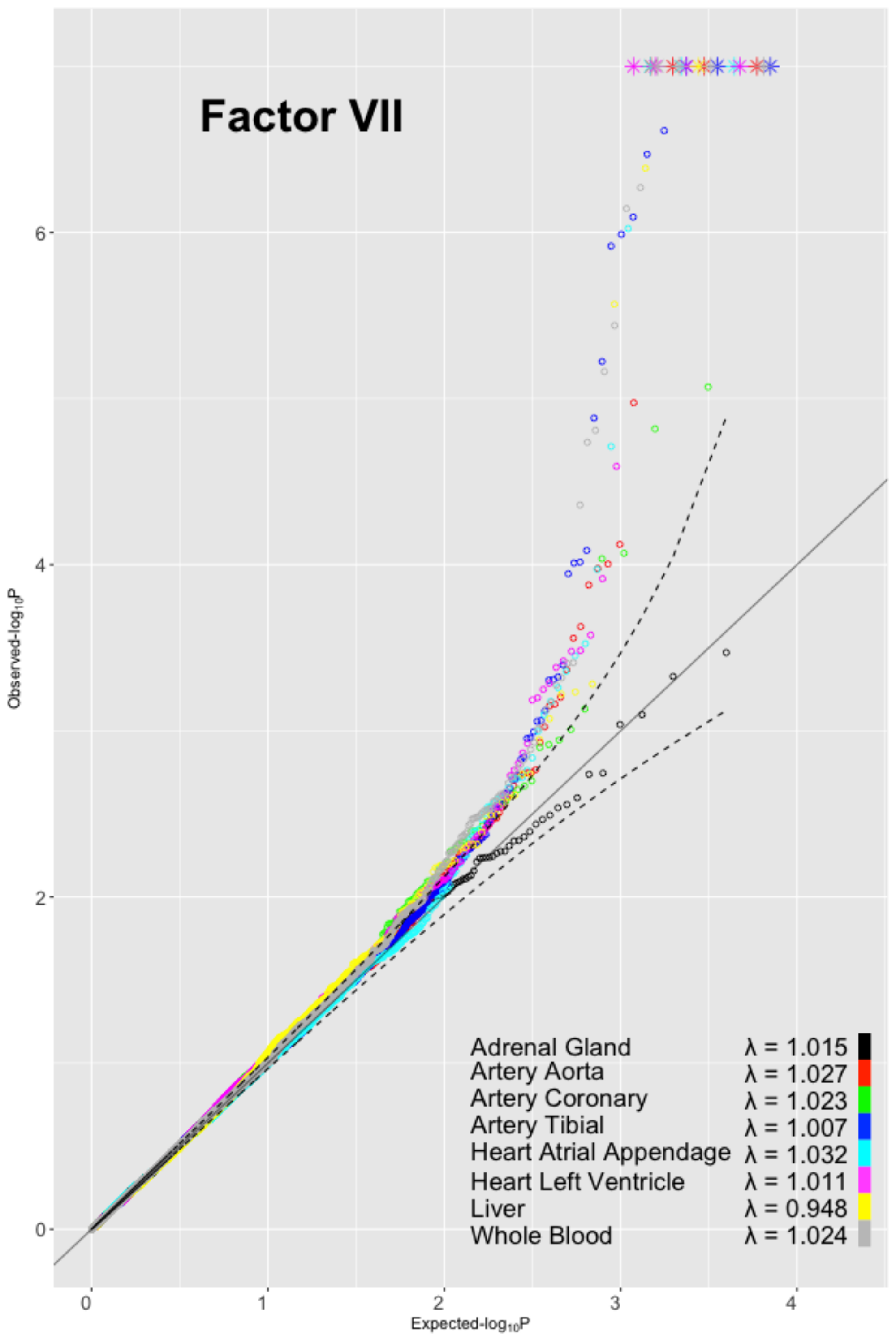


n) RR interval


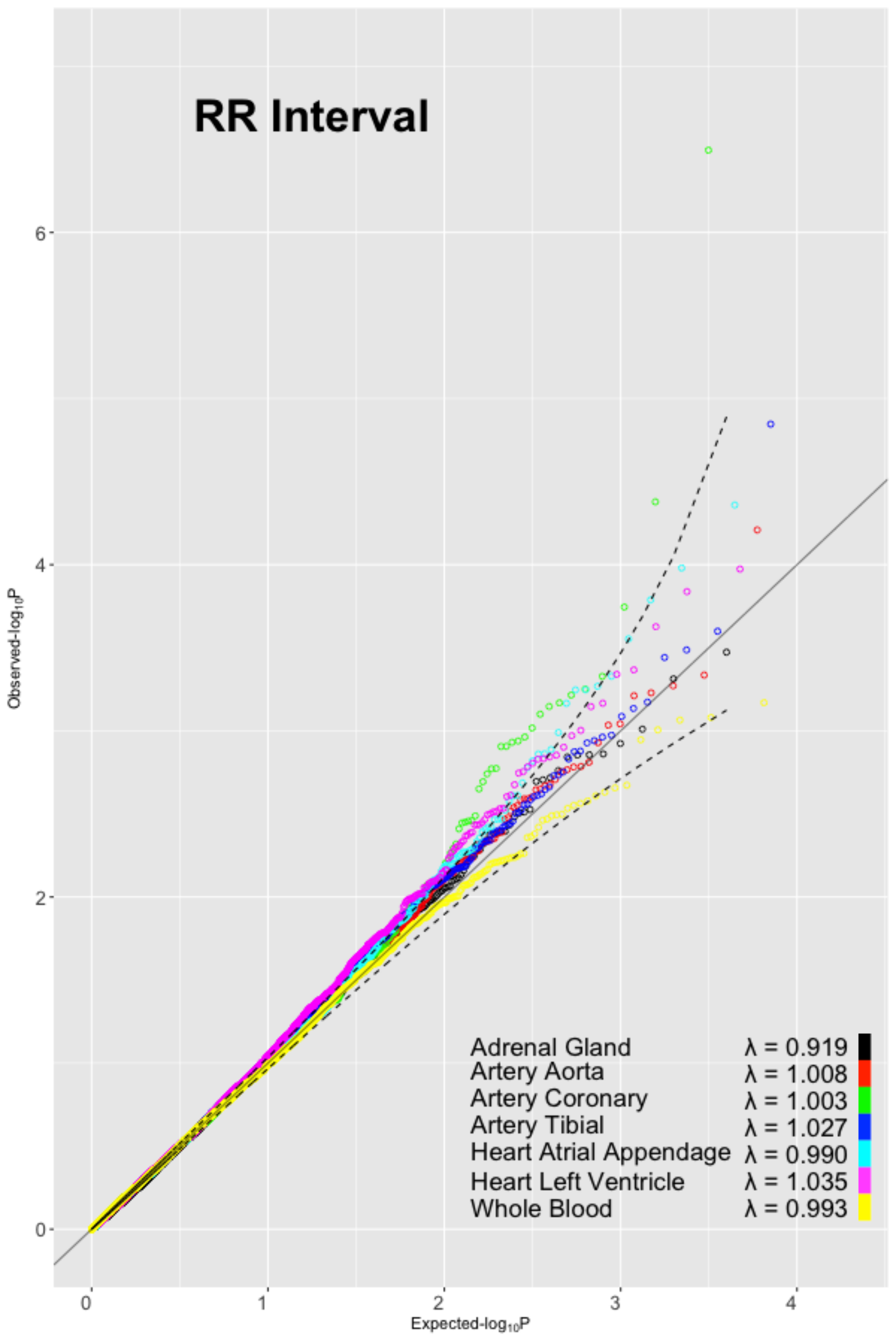

Supplement: Supplementary Data [file ddy435_supp.zip › SupplementaryFigures_final.docx]
