# Supplementary material for: Functionally oriented analysis of cardiometabolic traits in a trans-ethnic sample
Source: Hum Mol Genet. 2019 Jan 8;28(7):1212–24. doi: 10.1093/hmg/ddy435 (PMC6423424; doi:10.1093/hmg/ddy435)
Supplement: Supplementary Data [file ddy435_supp.zip › SupplementaryNote.docx]

ARIC RNA sequencing

RNA was isolated at UTHealth School of Public Health from PAXgene preserved whole blood of ARIC study participants using the MagMAX for Stabilized Blood Tubes RNA Isolation Kit (ThermoFisher Scientific, Waltham, MA). Total RNA isolation was followed by use of the GLOBINclear Kit (ThermoFisher Scientific) for the purpose of reducing >95% of the alpha and beta globin mRNA. RNA sequencing was performed at the Baylor College of Medicine Human Genome Sequencing Center (BCM-HGSC) using a custom strand-specific, poly-A+ RNA-Seq library protocol^1; 2^. First, polyadenylated mRNA was isolated from 1 µg of high quality total RNA (RNA Integrity Number [RIN]≥7) with Oligo(dT)25 Dynabeads. Next, mRNA was fragmented by heating and converted to cDNA using Superscript III reverse transcriptase, which was followed by inclusion of dUTP during a second strand synthesis to introduce strand specificity. For Illumina paired-end library construction, the resultant cDNA was processed through end-repair and A-tailing, ligated with Illumina PE adapters, and then digested with 10 units of Uracil-DNA Glycosylase (New England BioLabs [NEB], Ipswich, MA; Cat. No. M0280L). Libraries were prepared on the Beckman BioMek FX^p^ robots (Beckman Coulter, Inc., Atlanta GA). Amplification of the libraries was performed for 13 PCR cycles using the Phusion High-Fidelity PCR Master Mix (NEB; Cat. No. M0531L), and 6-bp molecular barcodes were incorporated during PCR amplification. Libraries were purified with Agencourt AMPure XP beads (Beckman Coulter, Inc) after each enzymatic reaction, and after PCR amplification was quantified using Caliper GX electrophoresis system (Perkin Elmer, Waltham, MA) and libraries were pooled in equimolar amounts (5 libraries/pool). A set of 92 polyadenylated transcripts developed by the External RNA Controls Consortium (ERCC)^3^ were added at the beginning of library preparation in order to track sample fluctuations and monitor sequence metrics. Sequencing was carried out on the Illumina HiSeq 2000 (Illumina, Inc., San Diego, CA) with five libraries pooled per lane, which produced approximately 74M paired-end reads of 101 base pairs (bp) per sample. A read count matrix was generated with the R package GenomicAlignments^4^ from the reads uniquely aligned to the reference genome (GRCh37) by STAR^5^. DESeq2^6^ was then used to generate normalized expression profiles for each gene (n=123,970).

References for supplementary note:

1. Peters, T.L., Kumar, V., Polikepahad, S., Lin, F.Y., Sarabia, S.F., Liang, Y., Wang, W.L., Lazar, A.J., Doddapaneni, H., Chao, H., et al. (2015). BCOR-CCNB3 fusions are frequent in undifferentiated sarcomas of male children. Mod. Pathol. 28, 575-586.

2. Wang, L., Ni, X., Covington, K.R., Yang, B.Y., Shiu, J., Zhang, X., Xi, L., Meng, Q., Langridge, T., Drummond, J., et al. (2015). Genomic profiling of Sezary syndrome identifies alterations of key T cell signaling and differentiation genes. Nat. Genet. 47, 1426-1434.

3. Lemire, A., Lea, K., Batten, D., Jian Gu, S., Whitley, P., Bramlett, K., and Qu, L. (2011). Development of ERCC RNA Spike-In Control Mixes. Journal of Biomolecular Techniques : JBT 22, S46-S46.

4. Lawrence, M., Huber, W., Pages, H., Aboyoun, P., Carlson, M., Gentleman, R., Morgan, M.T., and Carey, V.J. (2013). Software for computing and annotating genomic ranges. PLoS Comput. Biol. 9, e1003118.

5. Dobin, A., Davis, C.A., Schlesinger, F., Drenkow, J., Zaleski, C., Jha, S., Batut, P., Chaisson, M., and Gingeras, T.R. (2013). STAR: ultrafast universal RNA-seq aligner. Bioinformatics 29, 15-21.

6. Love, M.I., Huber, W., and Anders, S. (2014). Moderated estimation of fold change and dispersion for RNA-seq data with DESeq2. Genome biology 15, 550.
